# Room temperature dinitrogen cleavage and hydrogenation with organometallic complexes of uranium

**DOI:** 10.1039/d5sc07194a

**Published:** 2025-10-14

**Authors:** Angus C. G. Shephard, Lucie Pedussaut, Linda De Marchi, Luca Demonti, Thayalan Rajeshkumar, Nicolas Casaretto, Laurent Maron, Grégory Danoun, Thomas Simler, Grégory Nocton

**Affiliations:** a LCM, CNRS, École Polytechnique, Institut Polytechnique de Paris, Route de Saclay 91120 Palaiseau France thomas.simler@polytechnique.edu gregory.nocton@polytechnique.edu; b LPCNO, Université de Toulouse-CNRS, INSA, UPS UMR 5215 Toulouse France

## Abstract

The conversion of dinitrogen into ammonia plays an important role in sustaining life on Earth and serves as a significant building block for our planet's future. The Haber–Bosch process, although a well-established method for converting hydrogen and nitrogen gases into ammonia using metal-based heterogeneous catalysts, requires an extensive industrial infrastructure, limiting its accessibility and flexibility. Molecular systems, whether supported or unsupported, offer the advantage of allowing fine-tuning of the metal properties and the involved elementary steps, which ultimately leads to a better understanding of the transformations. In this context, we present findings on the reactivity of dinitrogen with an organometallic uranium complex featuring the bulky Cp^ttt^ ligand (Cp^ttt^ = 1,2,4-tris(*tert*-butyl)cyclopentadienyl). This complex demonstrates the ability to cleave and hydrogenate dinitrogen under mild conditions, at ambient temperature and atmospheric pressure. Most notably, the rich redox chemistry of uranium enables the direct reduction of N_2_ into a unique formal U^IV^ dimer featuring an end-on coordinated (N_2_)^4−^ bridging ligand, the cornerstone of the observed reactivity.

## Abstract 2

An organometallic uranium complex enables dinitrogen activation, cleavage, and hydrogenation at ambient temperature and pressure. The ligand's steric hindrance plays an essential role in this rare reactivity.

## Graphical abstract

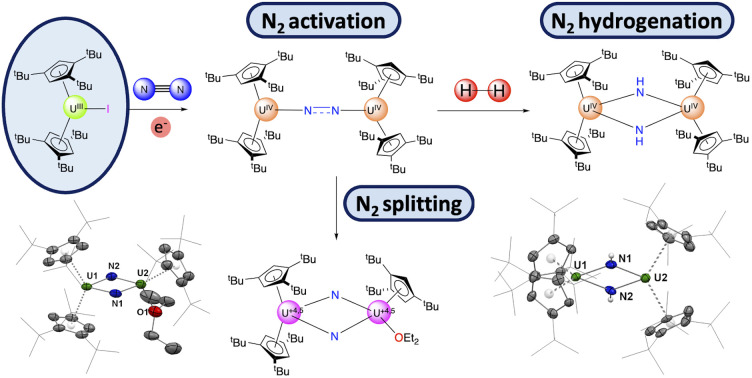

## Introduction

Ammonia is a molecule central to the issues of food and energy sovereignty.^1^ Its derivatives are widely used in agriculture and are also emerging as an alternative method for storing hydrogen, particularly for marine fuels.^2^ The question of how it is produced is central.^3,4^ The Haber–Bosch process, which is the formal hydrogenation of dinitrogen, has undoubtedly revolutionized access to this essential nitrogen source.^5^ Still, this industrial process remains carbon-intensive due to the current methods of hydrogen production.^6^ Blue and green alternatives to ammonia production are emerging, which tend to reduce environmental impact,^7^ while ammonia synthesis from alternative hydrogen sources is also being developed.^8–10^ The source of hydrogen primarily stems from the distinction between industrial and natural processes. Nitrogenases metabolize atmospheric dinitrogen, mobilizing eight protons and eight electrons to break apart one dinitrogen molecule and form two ammonia molecules and one dihydrogen molecule.^11^ In this catalytic cycle, the hydrogen atoms are supplied as protons, making the reaction highly beneficial from an environmental perspective. This strategy has been followed in the past few decades through several significant contributions in homogeneous nitrogen functionalization using early and middle transition metals, as exemplified by contributions from the Schrock,^12,13^ Nishibayashi,^14–16^ Peters,^17–19^ Liddle,^20^ and Mézailles groups.^21,22^ The sequential approach of fixing, activating, protonating, and reducing the metal center has enabled up to 60 000 catalytic cycles.^16^

In contrast, the molecular direct hydrogenation of dinitrogen, which is similar to the industrial Haber–Bosch process, yields substoichiometric amounts of ammonia after N_2_ cleavage.^23–27^ Besides transition metals, actinides are ideal candidates for nitrogen cleavage and functionalization due to their rich redox chemistry and proven ability to fix nitrogen.^28–32^ Several examples of uranium complexes have illustrated the reduction of dinitrogen and its subsequent cleavage, assisted by nearby cations or amido-phosphine ligands, as demonstrated by the Mazzanti^33–36^ and Zhu^25,37,38^ groups. Once formed, the resulting and highly reactive nitrido groups readily engage in reactions with different substrates to form various nitrogen compounds, including ammonium upon protonolysis.^28^ Yet, the formal hydrogenation of uranium nitrogen or nitrido species,^39,40^ especially when obtained from molecular dinitrogen,^25,33,41^ has been rarely witnessed. It should be noted that the formation of amine derivatives from dinitrogen does not always require uranium nitrido intermediates. For example, Arnold and co-workers recently showed that, using a bridged *meta*-xylyl-tetraphenolate U^IV^ dimer, reduction of the complex under N_2_ gave a hydrazido (N_2_H_2_)^2−^ species with concomitant deprotonation of the ligand scaffold. Cleavage of the remaining N–N bond and formation of ammonium occurred upon protonolysis of the complex.^42^

Easy access to U^III^ halide and amido precursors^43^ has significantly contributed to the development of uranium reductive chemistry^44^ with various ligand environments, allowing for the tuning of reductive properties and enabling the activation of diverse small molecules and substrates.^45–47^ Hydrocarbon ligands, *e.g.* cyclopentadienyl (Cp) and cyclooctatetraenyl (Cot), and related motifs are major assets in this field. In particular, U^III^ complexes in tris-Cp environments have led to the formation of rare examples of terminal N_2_ adducts,^48^ while reversible N_2_ coordination has been noted in the case of U^III^ pentalene complexes.^49^ The reactivity of U^III^ metallocene complexes typically relies on the rich redox chemistry of uranium, which can access up to the formal +VI oxidation state. In addition, as coordination saturation increases, steric crowding may also play a role in the reactivity. In highly sterically congested molecules, one extra electron can be provided to the metal system upon departure of one Cp ligand as a radical. Such sterically induced reduction (SIR) has allowed multi-electron reductions of various substrates.^50–52^

However, a balance exists between the bulkiness that either triggers SIR reactivity or kinetically stabilizes low-valent uranium species. The first molecular U^II^ complex was isolated in 2013 as the ion–pair complex [K(2.2.2-cryptand)][(Me_3_SiC_5_H_4_)_3_U],^53^ and the use of the bulky pentaisopropylcyclopentadienyl Cp^iPr5^ ligand allowed access to the first example of a neutral U^II^ organometallic sandwich complex.^54^ Further reduction of the latter led to the formation of a formal U^I^ complex as a charge-separated ion pair.^55^ The tris-substituted Cp^ttt^ ligand (Cp^ttt^ = 1,2,4-tris(*tert*-butyl)cyclopentadienyl), which presents a different steric profile compared to the Cp^iPr5^ ligand, proved effective in stabilizing non-classical divalent rare-earth complexes.^56–59^ Yet, such a ligand environment still provides an accessible coordination site,^60,61^ allowing for the coordination and reduction of small molecules such as CO and N_2_.^62–64^ In this context, our group recently showed that the isolable [LuCp^ttt^_2_] complex readily binds N_2_ to form an end-on (N=N)^2−^ complex. The latter can be hydrogenated under smooth conditions into a Lu–NH_2_ complex through a unique type of reactivity.^64^

Inspired by these findings, herein we present the room-temperature binding and cleavage of dinitrogen using an organometallic uranium complex featuring the Cp^ttt^ ligand. The redox chemistry of uranium contrasts with the single-electron transfer reactivity of divalent lanthanides and enables the isolation of the first example of an end-on uranium dimer with a formally four-electron reduced dinitrogen complex. Redox assistance by electron transfer from the Cp^ttt^ ligand ultimately leads to the six-electron N_2_ cleavage through an original type of reactivity that does not require the addition of further external reducing agents.^34,38,65^ As a result, bridged nitrido complexes are formed and can be hydrogenated at room temperature into uranium imido complexes. This work emphasizes the importance of the fine-tuning of the Cp ligand's steric properties for the generation of low-valent uranium species able to bind and achieve multiple (4 to 6) electron reductions of N_2_.

## Results and discussion

### Syntheses

The known U^III^ sandwich complex [Cp^ttt^_2_UI], 1,^66^ supported by the bulky Cp^ttt^ ligand, readily reacts with potassium graphite (KC_8_) at room temperature under an N_2_ atmosphere in aromatic hydrocarbon solvents, such as toluene and benzene, leading to a color evolution from deep blue to black. The reaction proceeded at various rates depending on the stoichiometry of KC_8_ and the N_2_ pressure. Under low pressures (1 atm or less) and with a high excess of reductant (10 equiv.), the ^1^H NMR spectrum showed a major set of signals within 60 min with no trace of starting material, indicating complete conversion of 1.

**Scheme 1:**
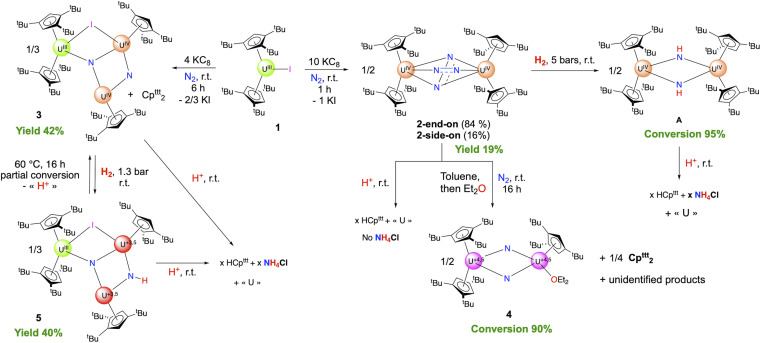
Reactivity scheme for the formation of the compounds described in this article (1–5, and A). *x* varies with the compound (see the text). The +3.5 and +4.5 oxidation states result from charge balance and are given as an average. The extent of delocalization was not determined spectroscopically; Table S27 shows the computed natural charges in 4. The conversions are deduced from ^1^H NMR data (see SI), and the yields correspond to the combined synthesis and crystallization yields.

When a lower excess of KC_8_ was used (4 equiv.), the same set of ^1^H NMR signals was transiently observed while the conversion was not complete after 60 min, as evidenced by the presence of remaining amounts of 1. As the starting material was further consumed, the ^1^H NMR spectrum evolved, over 6 h at room temperature, into a new set of signals. The concentration of both reactions' dark pentane and toluene solutions resulted in dark green and dark brown crystals, respectively, suitable for X-ray diffraction (XRD) studies. The analyses of the structures revealed formation, in the first case, of the dimeric dinitrogen complex [(Cp^ttt^_2_U)_2_(μ-N_2_)] (2) and, in the second case, of a trimeric uranium cluster [Cp^ttt^_2_U(μ-I)(μ_3_-N)(μ-N)(UCp^ttt^)_2_] (3) featuring two nitrido groups (Scheme 1). The ^1^H NMR spectrum of crystals of 3 (see below for its structural characterization) corresponds to the second set of signals appearing with time when a moderate excess of KC_8_ (4 equiv.) was used.

When the reduction of 1 was performed under higher pressure of N_2_ (5 bars) using a large excess of KC_8_ (10 equiv.), the formation of 2 is transiently observed while a new set of signals appears along with significant amounts of an organic by-product identified as (Cp^ttt^)_2_ (Scheme 1). The formation of (Cp^ttt^)_2_ aligns with the departure of one of the Cp^ttt^ ligands as its radical form, which leads to the coupled bis(tris-*tert*-butyl-cyclopentadiene) compound. The loss of one Cp^ttt^ ligand as a radical is associated with the reduction of the metal ion, which has already been witnessed in f-block organometallic chemistry in the attempted synthesis of [(Cp^ttt^)_2_Eu^III^(X)] (X = Cl, F),^67,68^ or upon oxidation of [(Cp^ttt^)_2_U^IV^ =Y] (Y = O, N(*p*-tolyl)) centers.^69,70^ It is worth noting that, in both cases, the electron transfer from the ligand to the metal occurred in the presence of easily reducible metal centers (Eu^III^, U^V/VI^).

In parallel, when a toluene solution of 2 is left at room temperature, the same set of ^1^H NMR signals appear, but more slowly (Fig. S7). The identity of the formed product has been obtained upon re-crystallization from diethyl ether, and XRD studies revealed the formation of the dimeric [Cp^ttt^_2_U(μ-N)_2_{U(Cp^ttt^)(OEt_2_)}] uranium complex 4 (Scheme 1). In this complex, the dinitrogen unit has been cleaved into two nitrido groups, accompanied by the departure of one Cp^ttt^ ligand from one uranium center. The coordination sphere of the latter is completed by one diethyl ether molecule. Although 4 was isolated as XRD-suitable crystals, separation from other co-crystallized materials was tedious and did not produce analytically pure material, precluding full characterization of this complex. However, the conversion from 2 to 4 was followed by ^1^H NMR spectroscopy.

### Solution and solid-state structure of 2

The ^1^H NMR spectrum of analytically pure crystals of 2 matches the signals observed in the first reaction (Scheme 1, middle) using 1 atm. of N_2_ and 10 equiv. of KC_8_. A detailed spectrum analysis revealed two sets of signals, which can hardly be distinguished at room temperature and are mostly evidenced as shoulders. A variable temperature (VT) ^1^H NMR experiment led to a better resolution of these shoulders as a set of signals with the same symmetry and similar chemical shifts (Fig. S3 and S4). At −40 °C, the two species can be clearly identified and attributed to the end-on [(Cp^ttt^_2_U)_2_(μ-η^1^:η^1^-N_2_)] (2-end-on) and side-on [(Cp^ttt^_2_U)_2_(μ-η^2^:η^2^-N_2_)] (2-side-on) species in a 0.83 : 0.17 ratio, respectively. This ratio did not change significantly over the temperature range of −80 °C to +40 °C. This behaviour precludes performing a van't Hoff analysis to assess the thermodynamic data of a possible equilibrium between the two species. Although end-on N_2_ coordination has already been witnessed in uranium chemistry,^48,71–74^2-end-on is the first example of an end-on dinitrogen adduct between two uranium metal centers.

**Fig. 1:**
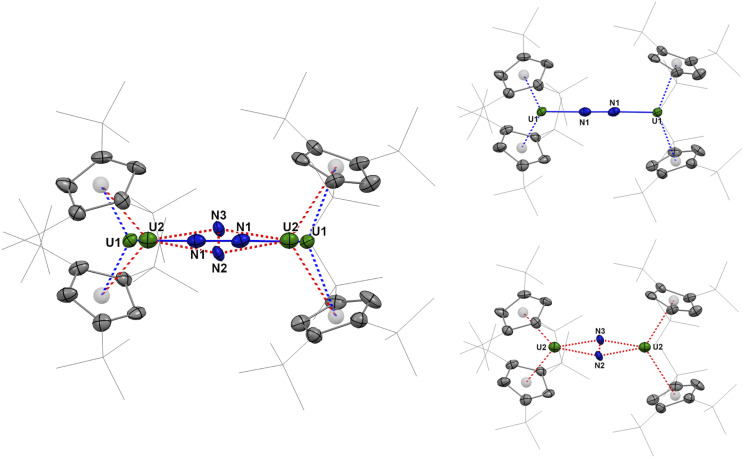
Molecular structure of 2 in the solid state, solved with a refined ratio of 74% for 2-end-on and 26% for 2-side-on. Hydrogen atoms are omitted for clarity. Thermal ellipsoids are shown at the 50% probability level.

The molecular structure of 2 in the solid state reveals two substructures with different coordination modes for the reduced dinitrogen ligand (Fig. 1 and Table S4), specifically the end-on (μ-η^1^:η^1^-N_2_) and side-on (μ-η^2^:η^2^-N_2_) motifs. In the XRD solid-state structure collected at 150 K, the respective ratio was refined to 0.74 : 0.26, which is in fair agreement with the ratio of 0.84 : 0.16 in solution at −40 °C obtained by ^1^H NMR spectroscopy. This ratio did not evolve either when the data were collected over the 150–230 K temperature range, while higher measurement temperatures resulted in degradation of the crystal.

For the minor component, 2-side-on, the U2–N average distance is 2.20(2) Å, the average U2-Cp(Ctr) distance 2.77 Å, and the N–N distance 1.37(6) Å. The U2–N distances observed in 2-side-on are within the range of those in the uranium side-on dinitrogen complexes reported in the literature, from 2.146 Å^33^ to 2.465 Å,^75^ with U⋯U separations ranging from 3.372 to 4.797 Å. The overall bond distances in 2-side-on are in agreement with a 4-electron reduction of dinitrogen and the formation of a side-on coordinated (N_2_)^4−^ bridging ligand, which has already been witnessed in uranium chemistry (see Table S6).^33,34,36,76^

For the major component, 2-end-on, the U1–N1 distance is 1.958(12) Å, the average U1-Cp(Ctr) distance is 2.51 Å, and the N–N distance is 1.43(2) Å. In addition to these altered metrics, the change in the coordination mode of the bridging dinitrogen ligand implies significant differences in the U⋯U separations (5.342(6) Å *vs.* 4.185(16) Å) as well as Cp(Ctr)-U-Cp(Ctr) angles (139° *vs.* 117°) for 2-end-on and 2-side-on, respectively. Only a few end-on coordinated heterodinuclear U-(μ-η^1^:η^1^-N_2_)-M (M = Fe, Re, Mo, Li) complexes have been reported in the literature,^71–74^ with U–N and N–N bond distances lying in the ranges of 2.221–2.606 Å and 1.139–1.232 Å, respectively. In these complexes, the bridging dinitrogen ligands have undergone a 2-electron reduction into (N_2_)^2−^ moieties. In comparison, the U–N bond distance in 2-end-on (1.958(12) Å) is significantly shorter, while the N–N separation of 1.43(2) Å is much larger. Relatively short U–N bond distances are typically found in U–N imido complexes, in which the distances vary with the oxidation state of the uranium center.^77–84^ The different metrics in 2-end-on compared to the abovementioned end-on coordinated uranium dinitrogen complexes account for a different reduction state of the bridging ligand, more precisely a 4-electron reduction of N_2_ to an end-on coordinated (N_2_)^4−^ ligand. Thus, the molecular structure of 2 is consistent with both end-on and side-on (N_2_)^4−^ ligands bridging two U^IV^ metal centers.

It may be noted that U^IV^ and Lu^III^ have similar crystal radii, which makes a comparison with lanthanide complexes relevant.^85^ Analysis of the metrics between end-on *vs.* side-on N_2_ coordination in lanthanide complexes is shown in the SI (Fig. S59 and Table S7). It reveals that, within identical ligand environments, the Ln–N bond distance is *ca.* 0.21 Å shorter in the end-on complexes while the Ln⋯Ln separation is *ca.* 1.1 Å longer.^62,86^ A similar trend is observed in our case with the U–N bond distance in 2-end-on being 0.24 Å shorter than in 2-side-on while the U⋯U separation is 1.2 Å longer.

The Raman spectrum was not informative, with only a broad feature and no difference with 2 prepared from ^15^N_2_, which contrasts with the well-defined Raman signal detected for the diazenido (N_2_)^2−^ ligand in the [(Cp^ttt^_2_Ln)_2_(μ-η^1^:η^1^-N_2_)] complexes.^62,64^ The different reduction state of the dinitrogen ligand as N_2_^4−^ in 2 could rationalize this observation, with a significant shift in the energy of the bond stretch and difference in oscillator strength.^33,42^ The elongated distance of the N–N moiety agrees with this statement,^87^ which accordingly lies in the range of hydrazido complexes.^42,88^

**Fig. 2:**
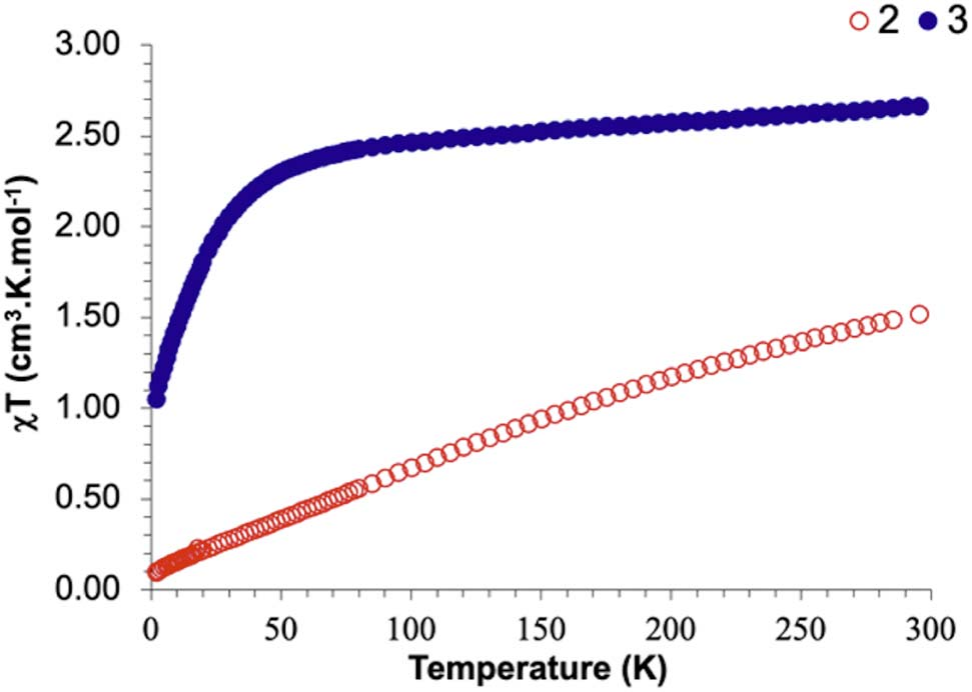
Solid-state temperature-dependent magnetic data for 2 (red unfilled dots) and 3 (blue filled dots). An aberrant point has been removed at 290 K for 2 (see Fig. S56).

The temperature-dependent solid-state magnetic data of 2 were recorded and feature a *χT* value of 1.53 cm^3^ K mol^−1^, *e.g.* 0.765 cm^3^ K mol^−1^ (2.47 μ_B_) for each U center (Fig. 2, S56 and S57). The *χT* value decreases monotonically with temperature to reach 0.09 *χT* cm^3^ K mol^−1^ at low temperature. This behavior indicates the presence of a non-magnetic ground state with low-lying magnetic crystal field states. Indeed, the absence of a maximum Curie temperature and the nearly linear evolution of *χT* until 70 K is typical for van Vleck paramagnetism arising from a second-order Zeeman effect, which is independent of the temperature (*χT* is linear with *T*). Van Vleck paramagnetism (TIP)^89–91^ is typically reported for U^IV^ complexes and thus agrees with a [(Cp^ttt^_2_U^IV^)_2_(μ-η^1^:η^1^-N_2_^4−^)] formula for 2.^92–96^

**Fig. 3:**
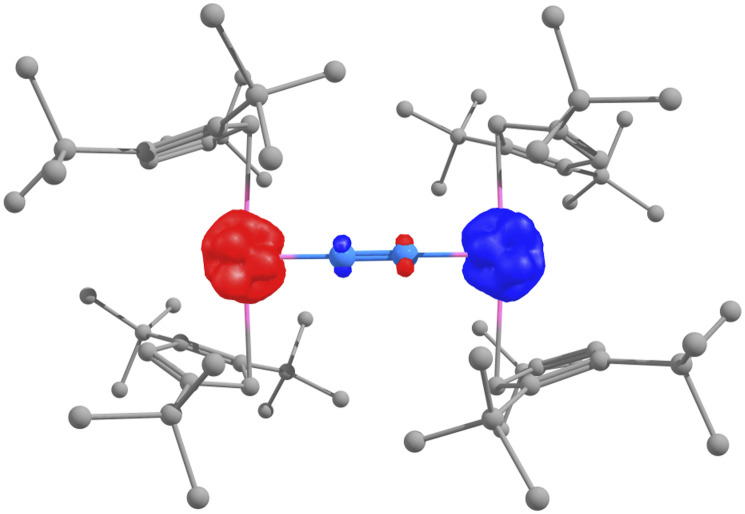
Unpaired spin density plot of the open-shell singlet ground state of 2-end-on.

DFT calculations (B3PW91 functional) were carried out to determine the electronic structure of 2 and the energy difference between the two N_2_ coordination modes. Computationally, both the side-on and end-on coordinations proved stable, in line with the experiment, with the end-on coordination appearing to be more favorable than the side-on by 9.8 kcal mol^−1^. Three different spin states were considered in the end-on case (septet, quintet, and open-shell singlet). The open-shell singlet appears to be the ground state (Table S8), as evidenced by the unpaired spin density plot (Fig. 3), and an (N_2_)^4−^ bridging ligand. Still, the quintet state is only lying slightly higher in energy (2.0 kcal mol^−1^ in enthalpy and 0.8 kcal mol^−1^ in Gibbs Free energy).

For both the 2-end-on and the 2-side-on complexes, the optimized structures are in fairly good agreement with the experimental ones. For 2-end-on, the structure is symmetrical, and the two U–N distances are 2.03 Å long for the open-shell singlet and 1.99 Å in the quintet (1.96 Å experimentally) with an N–N bond distance of 1.31 Å and 1.33 Å, respectively (1.43 Å experimentally). For the 2-side-on complex, the optimized structure is unsymmetrical with the shortest U–N bond distance of 2.21 Å (2.20 Å experimentally) with an N–N bond distance of 1.36 Å (1.37 Å experimentally). Although complexes bearing an (N_2_)^4−^ ligand are known in uranium chemistry,^33,34,36,76^ they have always exhibited side-on coordination since it allows a better overlap between the two N–N π* and the 5f orbital on the uranium center.

The situation is somewhat different here, and the bonding situation in 2-end-on was analyzed using Molecular Orbitals (MO), Nature Bonding Orbitals (NBO), and Quantum Theory of Atoms in Molecules (QTAIM) methods (Tables S10–S19 and Fig. S66). The HOMO and HOMO-1 are doubly occupied, where the alpha and beta parts are occupied, and are displaying two U–N π interactions, meaning an overlap between the 5f-6d hybrid orbital of U and the two π* of N_2_. This is also observed at the NBO level, where two formal U–N triple bonds are found. These bonds are polarized toward N(80%) and involve overlap between hybrid 5f–6d orbitals on U and either an sp hybrid on N (for the σ bond) or pure 2p orbitals (for the two π). A single N–N bond remains, being highly covalent (50–50 at the NBO) and of σ-type (overlap between two sp orbitals). The nature of the bonds in the system is finally confirmed by a QTAIM plot and an analysis of the critical points of the bond (BCP). Two U–N BCP are found with a large density in line with an iono-covalent bond, and the ellipticity value indicates the presence of a π character. On the other hand, the N–N BCP with a large density, a negative Laplacian, and almost zero ellipticity is indicative of a σ-type single bond. Therefore, all these analyses align with an N_2_^4−^ ligand. The preference for the end-on coordination over the side-on one for the four-electron reduction of N_2_ in 2 is due to the steric hindrance induced by the Cp^ttt^ ligand. Indeed, with a side-on coordination, the U⋯U distance is short so that the repulsion between the *tert*-butyl substituents is significant, while in an end-on coordination, the U⋯U distance remains long, decreasing the steric repulsion between the *tert*-butyl ligands. This repulsion is responsible for the unsymmetrical coordination in the 2-side-on complex (Tables S20–S25). The formation of both the 2-end-on and 2-side-on complexes was computed to be thermodynamically favorable by, respectively, 17.8 and 8.0 kcal.mol^−1^ in enthalpy in line with the experimental observation.

### Solution and solid-state structure of 3

The ^1^H NMR spectrum of 3 shows 18 identifiable signals (Fig. S19–S22), 12 of them with an integration of *ca.* 9H corresponding to the *tert*-butyl groups. The six remaining signals are lower in intensity, and three of them show significant isotropic shifts in agreement with the short distance to the uranium center of the aromatic protons of the Cp^ttt^ rings. The ^1^H NMR spectra at various temperatures secured this assignment. The number of signals aligns with a C_1_ symmetry, where the *tert*-butyl groups are magnetically independent, while two protons of the Cp^ttt^ rings could not be detected due to significant broadening.

**Fig. 4:**
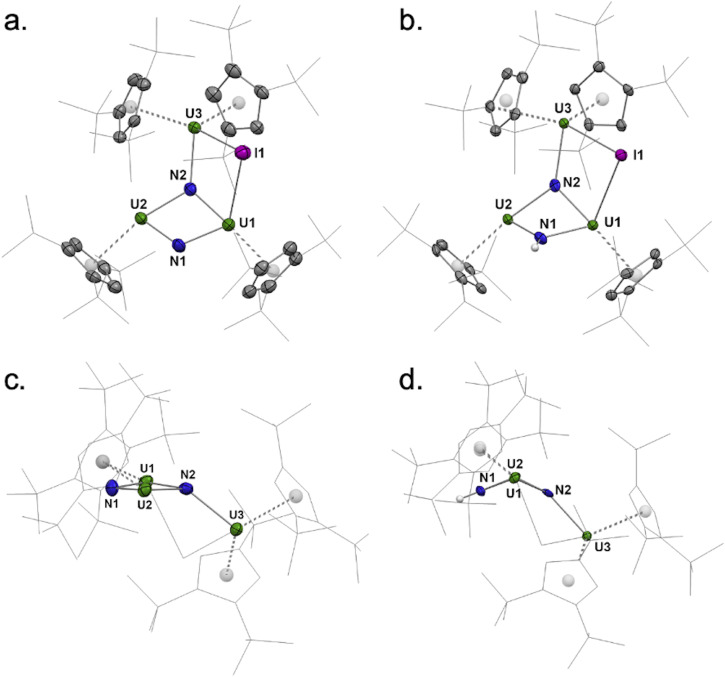
Molecular structures of 3 (a) and 5 (b) in the solid state. A different view of 3 (c) and 5 (d) showing the U1–N1–U2–N2 core is given. Hydrogen atoms (except for the NH unit) are omitted for clarity. Thermal ellipsoids are shown at the 50% probability level.

The XRD structure of 3 spans three uranium centers, two having one Cp^ttt^ ligand η^5^-coordinated (U1 and U2) and the last one (U3) coordinated to two Cp^ttt^ rings (Fig. 4 and Table S4). The latter uranium center coordinates to one iodine atom, bridging with the U1 center, with the U1–I1 and U3–I1 bond distances of 3.1065(3) and 3.2293(3) Å, respectively. One μ_3_-nitrido ligand (N2) is centered between the three uranium centers. One μ-nitrido ligand (N1) bridges the two uranium centers, which only bear one Cp^ttt^ ligand (U1 and U2). The U3-Cp(Ctr) average distance is 2.63 Å, and the Cp^ttt^-U3-Cp^ttt^ angle is 134°. The U1-Cp(Ctr) and U2-Cp(Ctr) distances are comparatively shorter, 2.51 Å and 2.55 Å, respectively, which may partly account for a lower coordination number compared to U3. The μ_3_-nitrido (N2) distances to the uranium centers are 2.221(3), 2.170(3), and 2.284(3) Å for U1–U3, respectively, comparable to those found in other μ_3_-nitrido uranium complexes, and are consistent with U–N single bonds.^37,97^ In contrast, the μ-nitrido (N1) distances are 2.027(3) and 2.033(3) Å for U1 and U2, respectively, in agreement with U–N multiple bond character.^34^ The differences in the U–I, U-Cp(Ctr), and U–N distances are globally in agreement with a different oxidation state for U3 compared to U1 and U2. From the observed bond distances and to balance the 11 negative charges, a tentative assignment of the oxidation states for the different uranium centers in 3 leads to formal U^IV^ centers for U1 and U2, while U3 corresponds to a formal U^III^ center. However, in the literature, similar distances have been reported for both U^III^ and U^IV^ complexes, and this sole metric should be taken cautiously.^66,69,98^

The temperature-dependent solid-state magnetic data of 3 present a *χT* value of 2.67 cm^3^ K mol^−1^ (Fig. 2). With decreasing temperature, the *χT* value decreases to 2.42 cm^3^ K mol^−1^ at 75 K and then rapidly to 0.53 cm^3^ K mol^−1^ at 2 K. The low-temperature value agrees with the presence of one U^III^ center, while the two other uranium centers are U^IV^ with nearly zero *χT* as observed for 2, which overall agrees with a formula of [Cp^ttt^_2_U^III^(μ-I)(μ_3_-N)(μ-N)(U^IV^Cp^ttt^)_2_] for 3.

### Solution and solid-state structure of 4

When left under 1 atm. of N_2_, the symmetrical ^1^H NMR spectrum of 2 evolves over several hours into a new set of numerous signals, indicative of a loss of symmetry in the product and the formation of multiple species. Among them, the formation of the (Cp^ttt^)_2_ dimer was evidenced, with characteristic signals at 1.30, 1.34, and 6.4 ppm.^70,99^ Degradation of 2 appears slower when the NMR tube is left under static vacuum, while it is significantly faster under higher N_2_ pressure (5 bars) (Fig. S7–S9 and Table S1). X-ray-suitable crystals of the degradation product, 4, were obtained from diethyl ether (Scheme 1). The ^1^H NMR spectrum of the crystallized product matches that of the principal degradation product of 2 (Fig. S35). However, it remains unclear whether 4 is obtained in a pure form or as a co-crystallization of several minor products. Once formed, 4 is thermally stable in solution, and its ^1^H NMR spectrum recorded at 80 °C reveals better-defined resonances, which are consistent with a non-symmetric species in solution (Fig. S31–S34).

**Fig. 5:**
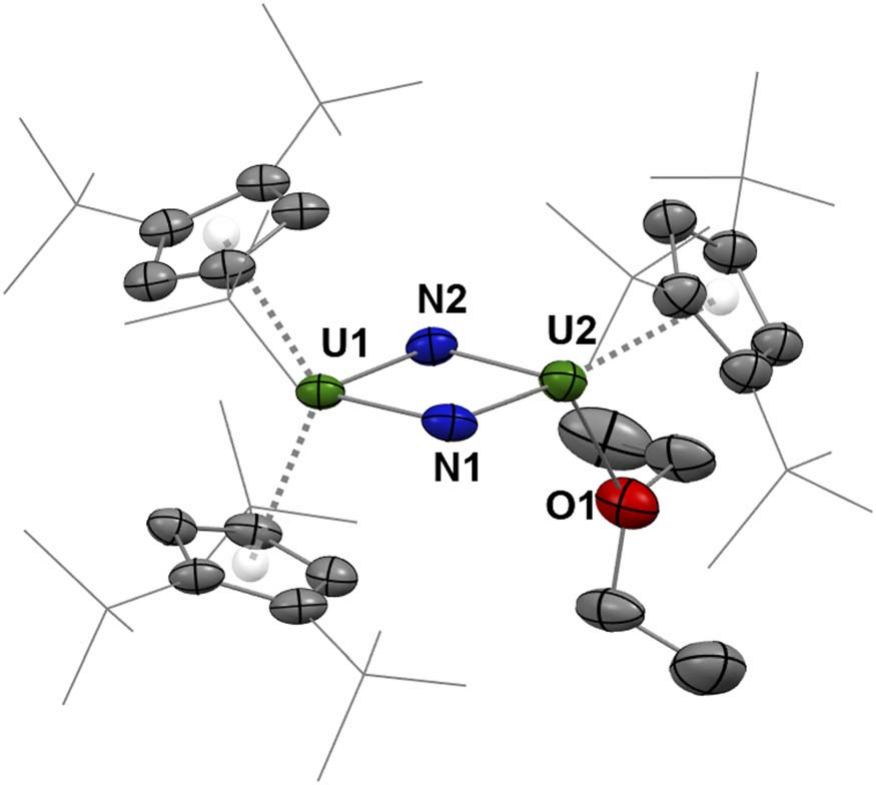
Molecular structure of one of the disordered positions of 4 in the solid state. Hydrogen atoms are omitted for clarity. Thermal ellipsoids are shown at the 50% probability level.

The molecular structure of 4 in the solid state reveals a dinuclear uranium complex bearing two bridging nitrido groups (Fig. 5 and Table S4). The first uranium ion, U1, is coordinated by two Cp^ttt^ ligands, and the second one, U2, by one Cp^ttt^ ligand–disordered over two positions–and one diethyl ether molecule. Complex 4, [Cp^ttt^_2_U(μ-N)_2_{U(Cp^ttt^)(OEt_2_)}], is a rare example of a molecular uranium bridged bis-nitrido complex, which is formed upon direct cleavage of dinitrogen.^35–37,65,97,100^ The short and almost equivalent U–N bond distances (2.031(7)–2.094(7) Å) are consistent with multiple bond character and agree with μ-nitrido groups,^28^ resulting in a planar and slightly distorted U1–N1–U2–N2 diamond core. The U1-Cp(Ctr) distances are 2.59 Å and 2.61 Å, which are intermediate between the U-Cp(Ctr) distances in 2-end-on and 2-side-on. The U2-Cp(Ctr) separations for both disordered components have similar distances (2.52 and 2.61 Å). From the charge balance in 4, with two N^3−^ nitrido groups and three Cp^ttt^ ligands, an average mixed valent +4.5 oxidation state is noted. However, the metrics alone do not allow for the assignment of the extent of the delocalization of the spin density over the two uranium centers.

Two spin states were computed for 4 (a doublet and a quartet), and both are very close in energy (0.6 kcal mol^−1^ in enthalpy favoring the quartet). However, in both cases, the system is a mixed-valence complex, U^IV^–U^V^, with either ferromagnetic coupling (quartet) or antiferromagnetic coupling (doublet) between the two uranium centers (Table S26–S32 and Fig. S68). The slight energy difference between the two situations aligns with a weak coupling.

### Hydrogenation and protonolysis experiments

**Scheme 2:**
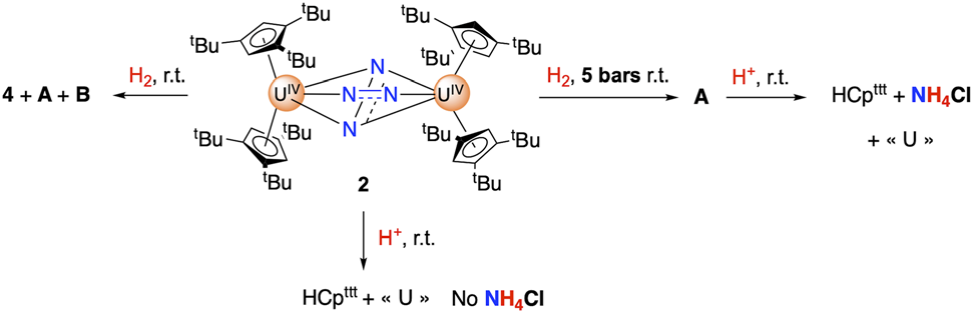
Hydrogenation and protonolysis reactions from 2.

The hydrogenation of 2 was investigated under different H_2_ pressures at room temperature, and monitored by ^1^H NMR spectroscopy (Scheme 2). Under 1 bar of H_2_, the reaction is slow and, as 2 naturally degrades at room temperature over *ca.* 24 h, the presence of new sets of signals is noted (Fig. S15). Under higher H_2_ pressures, the reaction is faster, with complete conversion of 2 observed within 12 h. Performing the reaction under 5 bars of H_2_ leads to the formation of one major species (A), while an additional product is observed under 3 bars (Fig. S12 and S14). Both species are symmetrical in solution with proton signals at *δ* 31.5, 12.7, −0.1, −35.9 in a 1 : 9 : 9 : 9 ratio for the first one (A), and two principal features at *δ* −6.5, −20.4 in a 1 : 2 ratio for the second one (B). Although A appears stable over time, we could not isolate an analytically pure material. Despite numerous crystallization attempts, crystals suitable for X-ray diffraction studies could only be obtained once.

**Fig. 6:**
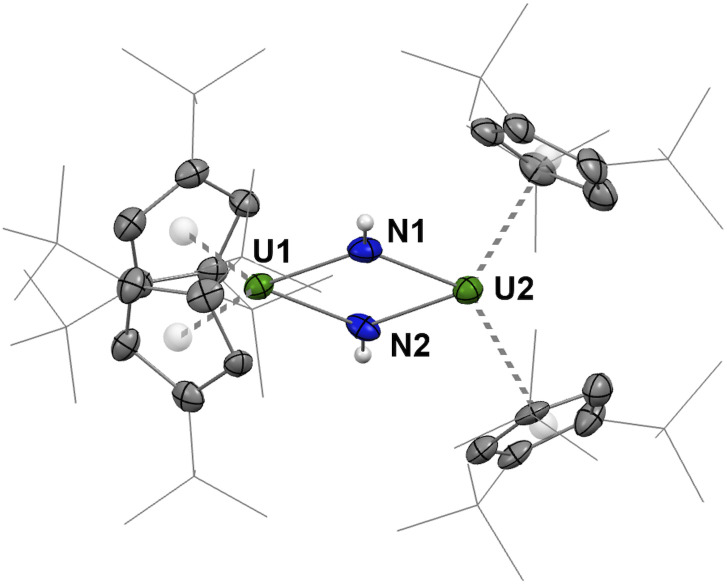
Molecular structure of one of the two independent molecules of A in the solid state. Hydrogen atoms (except for the NH units) are omitted for clarity. Thermal ellipsoids are shown at the 50% probability level.

The molecular structure of A (Fig. 6) unambiguously reveals the formation of the dinuclear bis-imido U^IV^ complex [(Cp^ttt^_2_U^IV^)_2_(μ-NH)_2_]. In particular, the hydrogen atoms on all imido units could be successfully located in the electron density map. Two independent molecules of A featuring very similar metrical data are found in the asymmetric unit (Fig. S63–S65). The U–N bond distances in A, from 2.188(6) to 2.214(6) Å, are longer than those in 4, in agreement with U–N single bonds, which supports hydrogenation of 2 and formation of a bridged bis-imido uranium complex. Accordingly, the U⋯U separation of *ca.* 3.62 Å in A is considerably longer than that in 4 (3.27 Å) and the U–N–U angles are *ca*. 5° larger in A. Interestingly, the U⋯C separations involving the Cp^ttt^ ligands in A span an unusually large range of distances, from 2.695(6) to 3.229(7) Å, which is more consistent with η^3^- rather than η^5^-coordination for the cyclopentadienyl ligands. Accordingly, relatively long U–Cp(Ctr) distances (2.671–2.705 Å) can be noted.

Protonolysis of 2 by the addition of excess HCl(Et_2_O) on 2 led to an immediate color change and the formation of HCp^ttt^ but no NH_4_Cl was detected in the ^1^H NMR spectrum (Fig. S11). However, the same protonolysis experiment performed either after hydrogenation of 2 into A or after letting a toluene solution of 2 degrade at room temperature led to the formation of NH_4_Cl (Fig. S13). In the corresponding ^1^H NMR spectra, the characteristic deshielded 1 : 1 : 1 triplet of NH_4_Cl with ^1^*J*(^14^N–H) = 48 Hz was observed (^14^N, *I* = 1, 99.6% natural abundance) and easily quantified by quantitative NMR spectroscopy (see the SI for details). Accordingly, when the isotopically labelled 2-^15^N was prepared from ^15^N_2_, its hydrogenation under a pressure of 5 bars, to form A-^15^N, and subsequent protonolysis led to the formation of ^15^NH_4_Cl featuring a deshielded doublet with ^1^*J*(^15^N–H) = 71 Hz (Fig. S16).

**Scheme 3:**
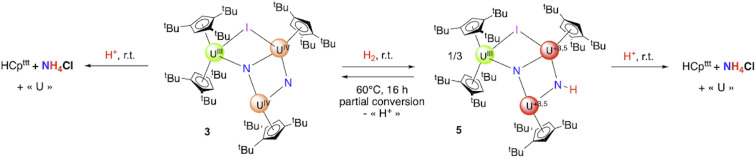
Hydrogenation and hydrolysis reaction from 3. The +3.5 oxidation state results from charge balance and is given as an average. The extent of delocalization was not determined.

Similar hydrogenation and protonolysis experiments were performed on 3. The hydrogenation under 1.2 bar H_2_ proceeds smoothly over 16 h to yield a clean compound crystallized from toluene as the hydrogenated uranium trimer 5 (Scheme 3). The XRD structure presents similar coordination environments for the three uranium centers as in the parent complex 3, with similar U–Cp(ctr) distances (2.51, 2.53, and 2.63 Å, for U1–U3, respectively, in 5, compared to 2.51, 2.55, and 2.63 Å in 3) (Fig. 5 and Table S4). The principal difference is that the U1–N1–U2–N2 core is slightly bent in agreement with a pyramidalization of the protonated nitrogen (N1) (Fig. 4c and d.).

Additionally, several U–N distances are significantly elongated in 5 compared to those in 3, particularly the U1–N1 and U2–N1 bond distances of 2.173(4) and 2.158(4) Å, respectively (compared to 2.027(3) Å and 2.033(3) Å in 3), consistent with a bridging imido (μ-NH).^39^ In contrast, the U–N bond distances involving the μ_3_-nitrido ligand (N2) are less altered: while the U3–N2 distance in 5 (2.315(3) Å) is slightly elongated compared to that in 3 (2.284(3) Å), the U1–N2 and U2–N2 distances (2.186(4) and 2.141(4) Å, respectively) are slightly shortened (2.221(3) and 2.170(3) Å in 3, respectively). The only minor changes in bond distances around N2 tend to indicate that the N2 atom is not protonated upon hydrogenation. However, a clear-cut conclusion cannot be formed from mere analysis of the XRD structures.

The ^1^H NMR spectrum of 5 shows 15 identifiable and paramagnetically shifted signals (Fig. S37–S40), two of them being broad with tentative integration, ten of them accounting for the twelve *tert*-butyl groups (two overlapping signals), and five signals for the aromatic protons of the Cp ring (instead of 8). Considering the significant isotropic shifts of some protons, several may likely experience too fast relaxation to be easily observed. The synthesis of 5 from D_2_ allowed assignment of the protonated nitrogen at *δ* 156.4 ppm (Fig. S46). Interestingly, in the solid-state, 3 also readily reacts with H_2_ at 60 °C to lead to 5 (Fig. S30). Compound 5 appears stable in the solid state at room temperature for several days. Yet, if 5 is left to stand in solution for one week at room temperature, the ^1^H NMR spectrum evolves with partial conversion back to 3 and formation of HCp^ttt^ (Fig. S44). The formation of 3 indicates that either the H_2_ addition is reversible or that the acidity of the imido proton is sufficient to protonate the Cp^ttt^ ligand. No H_2_ was detected in the solution by ^1^H NMR studies or by analyzing the volatiles by gas chromatography. Upon heating a toluene solution of 5 at 60 °C, a 90% conversion was observed over 16 h, leading to the formation of 3 and HCp^ttt^ (Fig. S45). This observation suggests that the degradation of 5 is triggered by the protonation of Cp^ttt^ ligands by the NH group,^23^ leading to partial conversion back into 3.

The controlled protonolysis of 3 was performed to evaluate the basicity of the N^3−^ ligands compared to that of the Cp^ttt^ ligands. The addition of 0.5 equiv. of pyridinium triflate or water led to partial consumption of 3 with the formation of several new species and only small amounts of HCp^ttt^ (Fig. S27 and S28). The stoichiometric addition of 1 equiv. of acidic protons led to the disappearance of 3 with pyridinium triflate but not with water. In both cases, adding 2 equiv. of H^+^ led to complete consumption of 3 with formation of new species, different for pyridinium triflate and water, along with minor amounts of HCp^ttt^. The formation of the new species, rather than HCp^ttt^, contrary to what was observed for 2, indicates preferential protonation of the basic N^3−^ ligands, which can explain the partial thermal reversibility of the hydrogenation of 3. Complex 5 may engage in acid–base reactions with itself, leading to partial regeneration of 3 and formation of by-products which degrade with loss of HCp^ttt^. Protonolysis of 3 and 5 with excess HCl(Et_2_O) led to the generation of NH_4_Cl in 69 and 44% yields, respectively, with the formation of the characteristic 1 : 1 : 1 triplet (^1^*J*_NH_ = 51 Hz) in the corresponding ^1^H NMR spectra (Fig. S29 and S47). All attempts to detect ammonia from the hydrogenation reactions of 2–5 were unsuccessful. As observed with the protonation of Cp^ttt^ ligands by the imido NH^2−^ group, the acidity of ammonia may lead to similar protonation events whenever formed.^23^

## Discussion

The reduction of [Cp^ttt^_2_UI] (1) with excess KC_8_ in toluene leads to two different products depending on the amount of reductant used. While using a small excess principally yields the cluster 3, formally featuring two U^IV^ and one U^III^ centers along with two nitrido ligands, the use of a larger excess of KC_8_ results in the formation of 2, best described as a dinuclear U^IV^ complex with two (N_2_)^4−^ ligands. The kinetics of the reduction might explain this difference. Indeed, under a significant excess of KC_8_, 1 is rapidly and quantitatively reduced into a transient [U(Cp^ttt^)_2_] divalent complex, which immediately reacts with N_2_. A similar behavior was observed for the non-classical divalent lanthanide [Ln(Cp^ttt^)_2_] (Ln = Gd, Tb, Dy, Lu) analogues.^62,64^ Contrary to the resulting [Ln(Cp^ttt^)_2_(μ-N_2_)] complexes, in which the thermodynamically favored +III oxidation state for the metals imposes the formation of an (N_2_)^2−^ ligand, the +III oxidation state in uranium is still highly reducing and allows further reduction into a bridging (N_2_)^4−^ ligand. The unique steric properties of the Cp^ttt^ ligand lead, for the reduced dinitrogen ligand, to both end-on (μ-η^1^:η^1^-N_2_)^4−^ and side-on (μ-η^2^:η^2^-N_2_)^4−^ coordination modes, the former being predominant (83% in solution and 74% in the solid state). Although four-electron reduction of dinitrogen has already been witnessed in uranium chemistry,^32^2 corresponds to the first example of an end-on coordinated (N_2_)^4−^ ligand between two uranium or f-element centers.

**Scheme 4:**
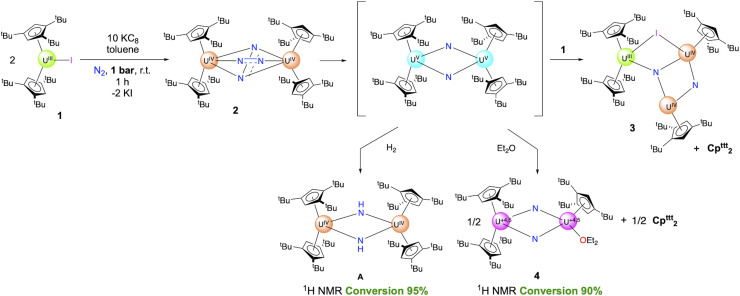
Summary scheme of the observed reactivity and proposed pathway.

Examples of similar linear M=N–N=M bis-imido complexes have been previously observed in early transition metal (group IV (Ti), group V (Nb, Ta), and group VI (W)) chemistry.^30^ In particular, a mixed cyclopentadienyl/amidate Ta^IV^=N–N=Ta^IV^ complex has been obtained upon reduction of the corresponding trichloride precursor under an N_2_ atmosphere and found to convert, above 0 °C, to the corresponding bis(μ-nitrido) Ta^V^ dinuclear complex.^101^ A similar pathway might be accessible in the case of 2, leading to the generation of the putative [(Cp^ttt^_2_U^V^)_2_(μ-N)_2_] complex featuring two bridging nitrido units (Scheme 4).

Indeed, the formation of the nitrido fragment “(Cp^ttt^_2_U^V^)(N)” from 2 was computed to be thermodynamically favorable with a weak N–N bond dissociation energy in 2 (17.0 kcal mol^−1^), resulting in an exergonic transformation (−1.3 kcal mol^−1^ in favor of the nitrido fragment). The DFT calculations also support thermodynamically driven transformations of the “(Cp^ttt^_2_U^V^)(N)” nitrido fragment into complexes A and 4 (Scheme S1). The hydrogenation of 2 under 5 bars H_2_, leading to the bis(imido) complex A provides further support for the transient generation of the bis(μ-nitrido) [(Cp^ttt^_2_U^V^)_2_(μ-N)_2_] complex. A similar hydrogenation reactivity on a dinuclear nitrido-bridged U^V^ complex supported by siloxide ligands has been reported by Mazzanti and co-workers.^102^

Owing to the oxidizing propensities of U^V^ centers, the loss of one Cp^ttt^ ligand as a radical from the putative [(Cp^ttt^_2_U^V^)_2_(μ-N)_2_] dimer, *via* an intramolecular single-electron transfer from one (Cp^ttt^)^−^ ligand to one uranium center, results in the formation of 4 along with the (Cp^ttt^)_2_ dimer (Scheme 4). Related single-electron transfer reactions involving the Cp^ttt^ ligand have already been witnessed in f-block organometallic chemistry, in association with easily reducible metal centers (Eu^III^, U^V/VI^).^67–70^ This step, which is accompanied by a release of steric pressure, can also be seen as an SIR.^51,52^ Indeed, as observed in the XRD structure of A, the strong steric crowding induced by the four Cp^ttt^ ligands results in an unusual η^3^-coordination mode for the cyclopentadienyl rings, with long U–Cp(Ctr) separations.

Similarly, the loss of a second Cp^ttt^ radical from 4 would result in the putative [(Cp^ttt^U^IV^)_2_(μ-N)_2_] complex, which upon coordination to one molecule of 1 yields complex 3. This possible pathway is consistent with the formation of 3 when a lower amount of KC_8_ is used, *i.e.* when the kinetics of the reduction of 1 enter in competition with those of the degradation of 2 into nitrido species.

Complexes 3 and 4 are rare examples of direct 6-electron cleavage of N_2_ by well-defined uranium dinitrogen complexes, without further addition of an external reducing agent.^32^ One system has been reported by the Zhu group, involving the assistance of the phosphine ligand side-arm on a dinuclear U^III^ complex.^38^ Besides, Mazzanti and co-workers have shown that reducing dinuclear U^III^ nitrido or oxo complexes under N_2_ can lead to cleavage of N_2_ and formation of bis-nitrido complexes.^34,36,65^ Here, we show that this challenging reactivity in uranium chemistry is not restricted to coordination complexes supported by N- or O-donor ligands but can be extended to organometallic uranium complexes featuring Cp-type ligands. In addition to their ability to stabilize low-valent species in f-block chemistry,^47^ substituted Cp ligands can provide possible redox assistance *via* single-electron transfer reactions, sterically induced^51^ or not, leading to a rich chemistry. Overall, the 6-electron cleavage of N_2_, resulting in the formation of 4 from the transient divalent [Cp^ttt^_2_U^II^], formally involves 5 electrons from the two uranium centers and one additional electron from an intramolecular ligand-to-metal electron transfer with the loss of one Cp^ttt^ radical. The loss of another Cp^ttt^ radical, leading to 3, provides one additional electron to the system.

It is noteworthy that the hydrogenation reactivity of 2-5 differs from that of the lanthanide complex [(Cp^ttt^_2_Lu)_2_(μ-η^1^:η^1^-N_2_)], for which formation of [Cp^ttt^_2_Lu(NH_2_)] occurred *via* direct hydrogenation of the reduced dinitrogen ligand.^64^ The rich redox chemistry of uranium, in which high-valent +V or +VI oxidation states are accessible, allows direct splitting of N_2_ into nitrido groups before hydrogenation. The other possible pathways to N_2_ hydrogenation, *i.e.*, by activation of H_2_ and reaction of the resulting hydride complexes with N_2_,^26^ or by direct synergistic H_2_/N_2_ activation,^27^ may be possible by tuning the system and are currently being investigated.

## Conclusions

The 1,2,4-tris-*tert*-butylcyclopentadienyl ligand is particularly well-suited for uranium in its low redox states, facilitating the smooth coordination and reduction of dinitrogen when combined with a reductant such as KC_8_. Depending on the amount of reductant used, two different complexes were isolated from the reduction of [Cp^ttt^_2_UI] under an N_2_ atmosphere. On the one hand, complex 2, [(Cp^ttt^_2_U)_2_(μ-N_2_)], is best described as a dinuclear U^IV^ complex in which the four-electron reduction of N_2_ to N_2_^4−^ is supported by XRD and solid-state magnetic data, as well as DFT computations. The unique steric profile of the Cp^ttt^ ligand, together with the size of uranium, allows for a bi-modal coordination of dinitrogen, featuring both side-on and end-on coordination modes. Notably, this complex features the first occurrence of an end-on (μ-η^1^:η^1^-N_2_)^4−^ ligand in f-block chemistry. At room temperature, 2 evolves into a new complex, 4, in which one Cp^ttt^ ligand has been removed and the reduced dinitrogen ligand has been cleaved into two N^3−^ nitride ions. This degradation is associated with the formation of (Cp^ttt^)_2_ and concomitant reduction of one uranium center. On the other hand, the trinuclear uranium complex 3 has been isolated by adjusting the amount of reductant used. In this complex, two Cp^ttt^ ligands have been removed as (Cp^ttt^)_2_, and one N_2_ molecule has been cleaved to form two N^3−^ nitride ions. Hydrogenation of the nitrido complexes occurs smoothly at room temperature in solution and in the solid state. Overall, this study highlights the rich reactivity of low-valent uranium organometallic complexes for N_2_ activation and cleavage.

## Author contributions

ACGS, LP, LDM, and LD performed the experimental work. NC solved the crystal structures. IDR and LM did the computational work. GD, TS, and GN analyzed and interpreted all the data. GN and TS wrote the manuscript with the help of GD. All authors contributed to the preparation of the SI. TS and GN managed the overall project.

## Conflicts of interest

There are no conflicts to declare.

## Supplementary Material

SC-OLF-D5SC07194A-s001

SC-OLF-D5SC07194A-s002

SC-OLF-D5SC07194A-s003

## Data Availability

CCDC 2464092 (2), 2464093 (3), 2464096 (4), 2464097 (5), and 2464099 (A) contain the supplementary crystallographic data for this paper.^103^ The data supporting this article have been included as part of the supplementary information (SI). Supplementary information: full experimental details, ^1^H NMR spectra, IR spectra, X-ray crystallographic details, magnetic analysis, and DFT calculations. See DOI: https://doi.org/10.1039/d5sc07194a.
