# Supplementary material for: Room temperature dinitrogen cleavage and hydrogenation with organometallic complexes of uranium
Source: Chem Sci. 2025 Oct 14;16(45):21334–45. doi: 10.1039/d5sc07194a (PMC12570077; doi:10.1039/d5sc07194a)
Supplement: SC-016-D5SC07194A-s002 [file SC-016-D5SC07194A-s002.pdf]

Supplementary Information (SI) for Chemical Science.
This journal is © The Royal Society of Chemistry 2025

# SUPPLEMENTARY INFORMATION
## For the manuscript

# Room temperature dinitrogen cleavage and hydrogenation with organometallic complexes of uranium

Angus C. G. Shephard,[#1] Lucie Pedussaut,[#1] Linda De Marchi,[1] Luca Demonti,[1] Thayalan Rajeshkumar,[2] Nicolas Casaretto,[1] Laurent Maron,[2] Grégory Danoun,[1] Thomas Simler*[1] and Grégory Nocton*[1]

[1] LCM, CNRS, Ecole Polytechnique, Institut Polytechnique de Paris, Route de Saclay, 91120 Palaiseau, France.

[2] LPCNO, UMR 5215, Université de Toulouse-CNRS, INSA, UPS, Toulouse, France

*Corresponding author: thomas.simler@polytechnique.edu, gregory.nocton@polytechnique.edu

# These authors made equal contributions.

## Table of content


1. Synthesis and characterization S3
   - 1.a. Materials and general procedures S3
   - 1.b. Synthesis of the complexes S4
   - 1.c. Protonolysis experiments S6
2. $^1$H NMR spectroscopy S8
   - 2.a. NMR spectra of $[(Cp^{ttt}{}_2U)_2(\mu\text{-}N_2)]$ (**2**) S8
   - 2.b. Temperature dependency of chemical shift signals for NMR signals of **2-end-on** S11
   - 2.c. Temperature dependency of chemical shift signals for NMR signals of **2-side-on** S12
   - 2.d. Degradation experiments for $[(Cp^{ttt}{}_2U)_2(\mu\text{-}N_2)]$ (**2**) S13
   - 2.e. NMR spectra for the protonolysis of $[(Cp^{ttt}{}_2U)_2(\mu\text{-}N_2)]$ (**2**) S15
   - 2.f. Hydrogenation of $[(Cp^{ttt}{}_2U)_2(\mu\text{-}N_2)]$ (**2**) and protonolysis S17
   - 2.g. NMR spectra of $[Cp^{ttt}{}_2U(\mu\text{-}I)(\mu_3\text{-}N)(\mu\text{-}N)(UCp^{ttt})_2]$ (**3**) S24
   - 2.h. Temperature dependency of chemical shift signals for NMR signals of **3** S27
   - 2.i. NMR spectra for the hydrogenation and protonolysis of **3** S29
   - 2.j. NMR spectra of $[Cp^{ttt}{}_2U(\mu\text{-}N)_2\{U(Cp^{ttt})(OEt_2)\}]$ (**4**) S33
   - 2.k. NMR spectra for the hydrogenation of $[Cp^{ttt}{}_2U(\mu\text{-}N)_2\{U(Cp^{ttt})(OEt_2)\}]$ (**4**) S36
   - 2.l. NMR spectra of $[Cp^{ttt}{}_2U(\mu\text{-}I)(\mu_3\text{-}N)(\mu\text{-}NH)(UCp^{ttt})_2]$ (**5**) S37
   - 2.m. Temperature dependency of chemical shift signals for NMR signals of **5** S39
   - 2.n. Dehydrogenation and deuteration of $[Cp^{ttt}{}_2U(\mu\text{-}I)(\mu_3\text{-}N)(\mu\text{-}NH)(UCp^{ttt})_2]$ (**5**) S42
   - 2.o. Protonolysis of $[Cp^{ttt}{}_2U(\mu\text{-}I)(\mu_3\text{-}N)(\mu\text{-}NH)(UCp^{ttt})_2]$ (**5**) S45
3. Infrared spectra S46
4. Raman spectra S50
5. UV/Visible Spectra S51
6. Magnetic measurements S52
7. X-ray crystallography S54
   - 7.a. General methods S54
   - 7.b. Summary of crystal data S55
   - 7.c. Crystal structure of $[(Cp^{ttt}{}_2U)_2(\mu\text{-}N_2)]$ (**2**) S57
   - 7.d. Analysis of the bond distance in Ln-$N_2$ complexes S57
   - 7.e. Crystal structure of $[Cp^{ttt}{}_2U(\mu\text{-}I)(\mu_3\text{-}N)(\mu\text{-}N)(UCp^{ttt})_2]$ (**3**) S61
   - 7.f. Crystal structure of $[Cp^{ttt}{}_2U(\mu\text{-}N)_2\{U(Cp^{ttt})(OEt_2)\}]$ (**4**) S61
   - 7.g. Crystal structure of $[Cp^{ttt}{}_2U(\mu\text{-}I)(\mu_3\text{-}N)(\mu\text{-}NH)(UCp^{ttt})_2]$ (**5**) S62
   - 7.h. Crystal structure of $[(Cp^{ttt}{}_2U)_2(\mu\text{-}NH)_2]$ (**A**) S63
8. Computational details S65
9. References S99

# 1. Synthesis and characterization

## 1.a. Materials and general procedures

All manipulations were performed under nitrogen or argon atmosphere, using standard Schlenk techniques or in gloveboxes (MBraun, < 1 ppm $O_2$, < 1 ppm $H_2O$). Toluene and pentane were dried using an MBraun solvent purification system (SPS-800) and degassed by three freeze-pump-thaw cycles before use. Other solvents (THF, THF-$d_8$, tol-$d_8$ and $C_6D_6$) were dried over sodium benzophenone, degassed and transferred under reduced pressure in a cold J-Young flask before use. $HCp^{ttt}$,[1] $KC_8$,[2] and $[UI_3(dioxane)_{1.5}]$[3] were prepared using literature procedures. $KCp^{ttt}$ was prepared by deprotonation of $HCp^{ttt}$ with $KN(SiMe_3)_2$ in $Et_2O$. All other chemicals where obtained from commercial sources and used without further purification.

High purity $H_2$ gas (Alphagaz 2, $H_2 \geq 99.9999$ %, < 0.1 ppm $CO_2$, < 0.1 ppm $O_2$, < 0.5 ppm $H_2O$) was purchased form Air Liquide. Isotopically enriched $D_2$ (99.9% enrichment) and $^{15}N_2$ (99.8% enrichment) were purchased form Eurisotop. For reactions involving gasses, the indicated pressures correspond to absolute gas pressures.

$^1H$ NMR spectra were recorded on a Bruker Avance III-300 MHz spectrometer. The chemical shifts were referenced to residual solvent signals. Data were processed using MestReNova software. Infrared spectra (4000 – 400 $cm^{-1}$) were obtained by ATR with a Thermo Scientific Nicolet iS5 FTIR spectrometer equipped with an iD7 ATR-Diamond unit. UV-Vis-NIR absorption spectra were recorded on an Agilent Cary 60 UV-Vis spectrometer in quartz cuvettes of 20 mm or 100 mm width adapted with J. Young valves. Raman spectra were recorded on solid samples in sealed glass capillaries on a LabRAM HR Evolution Raman spectrometer (Horiba Scientific) using a 473 nm laser at 50% power irradiation, with 10 second acquisitions and an 1800 $mm^{-1}$ grating. Magnetic measurements were recorded on solid samples sealed in 3 mm quartz tubes with a SQUID magnetometer (MPMS Quantum Design). Elemental analyses were obtained from Mikroanalytisches Labor Pascher (Remagen, Germany).

**Caution:** Depleted uranium (primary isotope $^{238}U$) is a weak α-emitter (4.197 MeV) with a half-life of 4.47 x 109 years. Manipulations and reactions should be carried out in monitored fume hoods or in an inert glovebox in a radiation laboratory equipped with α- and β-counting equipment.

## 1.b. Synthesis of the complexes

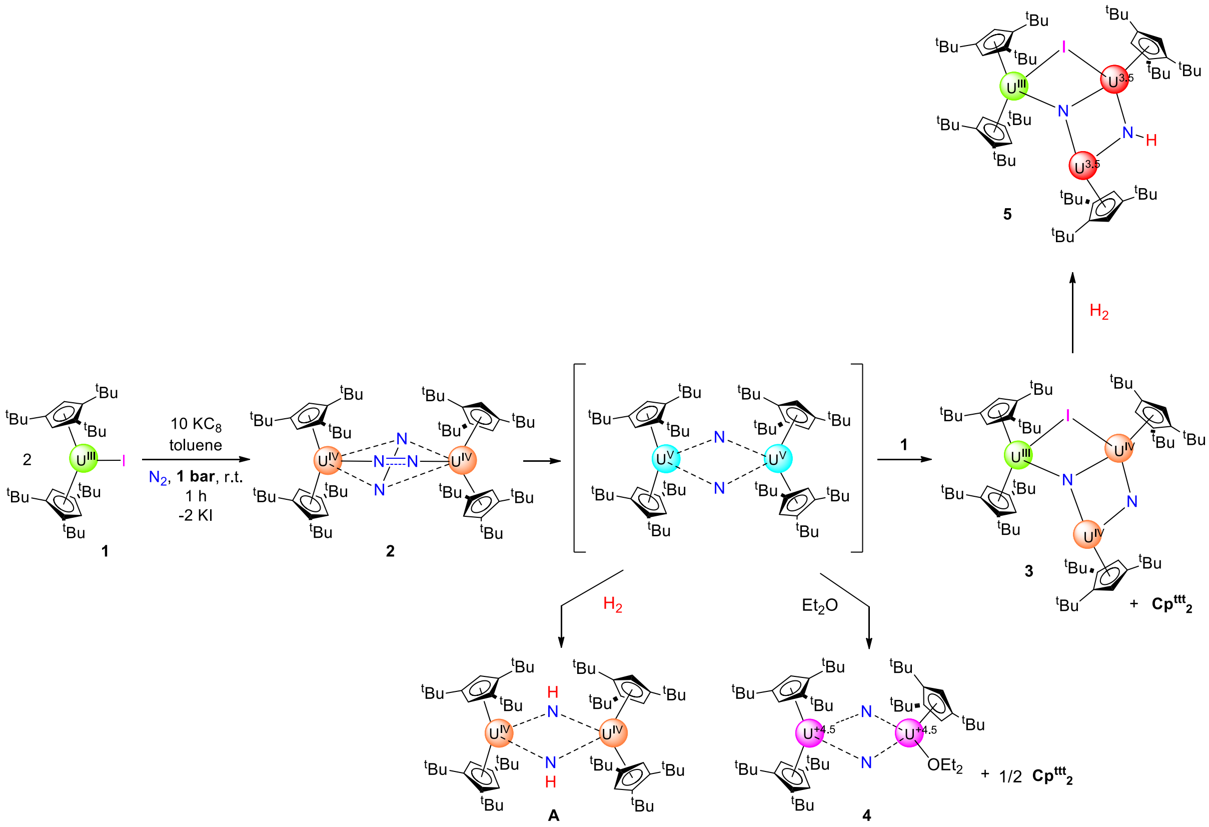


**Figure S1.** Overall reactivity scheme and numbering of the compounds **1-5**.

### Synthesis of [Cp$^{ttt}{}_2$UI] (1).

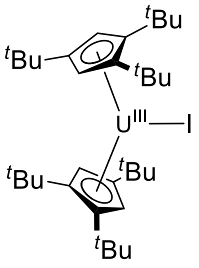

A J. Young Schlenk flask equipped with a magnetic stirrer bar was charged with [$UI_3$(dioxane)$_{1.5}$] (0.152 g, 0.20 mmol), KCp$^{ttt}$ (0.108 g, 0.40 mmol), and anhydrous toluene (5 mL). The reaction mixture was stirred at 110 °C for three days. The resulting deep blue solution was dried under reduced pressure, and the solids extracted with pentane (5 x 5 mL), concentrated under reduced pressure, and stored at -40 °C, yielding dark blue crystals of [Cp$^{ttt}{}_2$UI], **1**, (0.124 g, 74%).

$^1$H NMR spectral data is in agreement with that described in the literature.[4]

### Synthesis of [(Cp$^{ttt}{}_2$U)$_2$(μ-η$^1$:η$^1$-$N_2$)] (2)

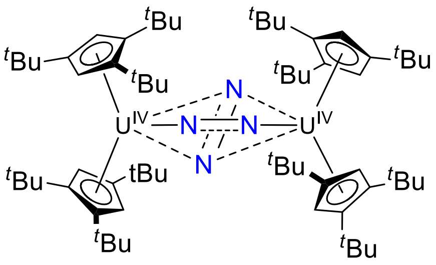

Under a nitrogen atmosphere, a vial equipped with a magnetic stirrer bar was charged with [Cp$^{ttt}{}_2$UI], **1**, (0.052 g, 0.063 mmol) and $KC_8$ (0.087 g, 0.63 mmol, 10 equiv.) and anhydrous toluene (1.7 mL). The reaction mixture immediately darkened from blue to black. The mixture was allowed to stir for 1 hour before filtering the mixture and removing the solvent under reduced pressure. The solid residue was washed with a minimal amount of pentane (2 x 2 mL), affording **2** as a black powder (8.4 mg, 5.8 μmol,19%).

Crystals suitable for XRD were obtained by dissolving the powder in pentane. The pentane solution was concentrated and stored at -40 °C, affording crystals of **2** as dark green needles.

$^1$H NMR (300 MHz, $C_6D_6$, 25 °C): δ 39.64 (br s, 4 H, Cp-*H*), 25.13 (br s, 4 H, Cp-*H*), 11.71 (br s, 36 H, $^t$Bu), -9.35 (br s, 36 H, $^t$Bu), -26.27 (br s, 36 H, $^t$Bu).

IR (ATR, cm$^{-1}$): 2954 m, 2905 m, 2863 m, 1478 w, 1454 m, 1385 s, 1355 s, 1239 s, 1209 m, 1184 m, 1165 m, 1114 m, 1025 w, 999 s, 956 s, 892 s, 670 s.

UV-vis-NIR (toluene, 298 K, C = 7.27 x $10^{-4}$ M) $\lambda_{max}$/nm: 394, 472, 595, 657

Elemental Analysis Calcd. for $C_{68}H_{116}N_2U_2$ (1437.72 g·mol$^{-1}$): C, 56.81; H, 8.13; N, 1.95%. Found C, 57.67; H, 8.09; N, 1.88%.

**<u>Synthesis of [$Cp^{ttt}_2U(\mu\text{-}I)(\mu_3\text{-}N)(\mu\text{-}N)(UCp^{ttt})_2$] (3).</u>**

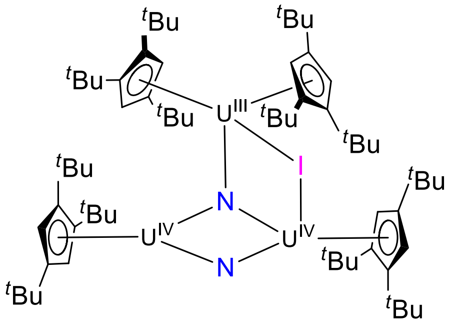


Under a nitrogen atmosphere, a vial equipped with a magnetic stirrer bar was charged with [$Cp^{ttt}_2UI$], **1**, (0.120 g; 0.14 mmol) and $KC_8$ (0.072 g, 0.53 mmol, *ca.* 4 equiv.) and anhydrous toluene (3.0 mL). The reaction mixture was allowed to stir for 6 hours at room temperature before isolating the solution by filtration. The resulting solution was allowed to stand at room temperature for 24 hours before being concentrated under reduced pressure and stored at -40 °C, affording **3** as dark brown blocks (0.036 g, 42%).

$^1$H NMR (300 MHz, toluene-*d*$_8$, 25 °C): δ 123.50 (Cp-*H*), 117.74 (Cp-*H*), 33.88 ($^t$Bu), 12.61 ($^t$Bu), 11.15 ($^t$Bu), -2.81 ($^t$Bu), -5.53 ($^t$Bu), -17.52 (Cp-*H*), -23.78 ($^t$Bu), -29.48 ($^t$Bu), -30.64 ($^t$Bu), -31.33 ($^t$Bu), -44.18 (Cp-*H*), -49.11 ($^t$Bu), -59.03 ($^t$Bu), -191.15 (Cp-*H*).

Note that some Cp-*H* signals could not be identified. In addition, one $^t$Bu signal is missing in the $^1$H NMR spectrum in tol-*d*$_8$ at room temperature (see Fig. S19-S20), possibly due to the fluxional behavior of the Cp$^{ttt}$ ligands. All $^t$Bu signals could be identified in spectrum at 80 °C (Fig. S21-22).

$^1$H NMR (300 MHz, toluene-*d*$_8$, 80 °C): δ 94.61 (Cp-*H*), 93.07 (Cp-*H*), 26.45 ($^t$Bu), 9.74 ($^t$Bu), 7.98 ($^t$Bu), -2.03 ($^t$Bu), -4.23 ($^t$Bu), -7.68 (Cp-*H*), -12.58 (Cp-H), -19.63 ($^t$Bu), -22.08 ($^t$Bu), -22.46 ($^t$Bu), -24.26 ($^t$Bu), -33.12 (Cp-*H*), -36.47 ($^t$Bu), -38.51 ($^t$Bu), -42.56 ($^t$Bu), -147.06 (Cp-*H*).

IR (ATR, cm$^{-1}$): 2951 m, 2900 m, 2861 m, 2157 m, 1596 m, 1494 w, 1455 m, 1385 m, 1356 s, 1269 w, 1356 s, 1269 w, 1237 s, 1199 m, 1162 m, 1111 w, 1072 w, 1027 w, 999 m, 955 m, 918 w, 893 w, 818 m, 796 w, 727 s, 693 s, 671 s.

UV-vis-NIR (pentane, 298 K, C = 4.99 x $10^{-5}$ M) $\lambda_{max}$/nm: 263, 398, 657

Elemental Analysis Calc. for $C_{89}H_{137}IN_2U_3$ (2076.04 g·mol$^{-1}$, after loss of one lattice toluene molecule): C, 51.49; H, 6.65; N, 1.35%. Found: C, 51.05; H, 6.95; N, 1.54%

**Isolation of [$Cp^{ttt}{}_2U(\mu\text{-}N)_2\{U(Cp^{ttt})(OEt_2)\}$] (4)**

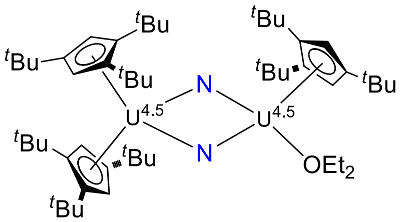


Under a nitrogen atmosphere, a vial equipped with a magnetic stirrer bar was charged with [$Cp^{ttt}{}_2UI$], **1**, (14 mg, 0.017 mmol, 1 equiv.) and $KC_8$ (23 mg, 0.17 mmol, 10 equiv.) and anhydrous toluene (0.8 mL). The reaction mixture immediately darkened from blue to black. The mixture was allowed to stir for 1 hour to obtain **2**. The reaction mixture was then filtered and held at room temperature for two days to allow full degradation of **2**. After evaporation of toluene, the resulting solid was dissolved in $Et_2O$, and the solution concentrated almost to dryness and let to crystalize at -40 °C. After 2 days, crystals of **4** suitable for X-ray diffraction were obtained.

$^1H$ NMR (300 MHz, toluene-$d_8$, 25 °C): δ 5.14, 3.20, -3.50, -8.54, -11.50, -13.20, -17.13, -19.75 (main signals)

IR (ATR, $cm^{-1}$): 2953 m, 2870 w, 1459 s, 1388 s, 1359 s, 1237 s, 1197 s, 1164 s, 1111 s, 998 s, 920 s, 959 s, 882 s, 805 s, 732 s, 672 s.

**Synthesis of [$Cp^{ttt}{}_2U(\mu\text{-}I)(\mu_3\text{-}N)(\mu\text{-}NH)(UCp^{ttt})_2$] (5)**

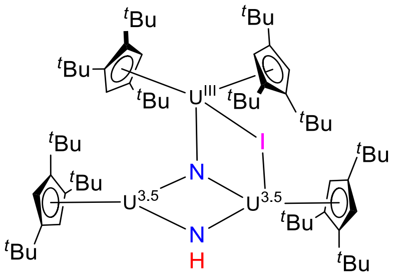


A J. Young NMR tube was charged with **3** (20 mg, 0.011 mmol) and dissolved in toluene-$d_8$ (0.3 mL). The resulting solution was frozen, placed under reduced pressure, and an $H_2$ atmosphere (1.2 bar, excess) was introduced. The NMR tube was then stirred overnight, resulting in a change in color from brown to crimson. Storage of the concentrated toluene solution at -40 °C led to crystals of **5** as crimson blocks (8 mg, 40%).

$^1H$ NMR (300 MHz, toluene-$d_8$, 25 °C): δ 156.40 (br s, 1H), 94.09 (s, 1H), 91.40 (br s, 1H), 24.04 (s, 1H), 15.09 (s, 9H), 7.23 (s, 9H), -2.10 (s, xH, br), -14.23 (s, 9H), -18.81 (s, 9H), -21.79 (s, 9H), -24.54 (s, 9H), -27.70 (s, xH, br), -33.95 (s, 1H), -42.36 (s, 18H), -57.46 (s, 9H).

IR (ATR, $cm^{-1}$): 3364 m, 2954 m, 2902 m, 2863 m, 2273 m, 2119 m, 2049 w, 1570 w, 1455 s, 1386 s, 1357 s, 1236 s, 1197 m, 1162 s, 1020 w, 999 s, 955 s, 868 m, 821 s, 787 s, 745 m, 685 m, 676 s, 666 m.

UV-vis-NIR (pentane, 298 K, C = 3.54 x $10^{-5}$ M) $\lambda_{max}$/nm: 392, 518, 588, 670, 810

Elemental Analysis Calc. for $C_{86,5}H_{137}IN_2U_3$ (2045.95 g·$mol^{-1}$, after loss of 1/2 lattice toluene molecule): C, 50.40; H, 5.40; N, 1.70%. Found: C, 50.78; H, 6.75; N, 1.70%. Despite our effort, the elemental analysis for H was not satisfactory.

### 1.c. Protonolysis experiments

**General procedure:** Protonolysis experiments were performed by adding 100 µL of $HCl(Et_2O)$ (2 M) on the reaction media. All volatiles were removed under reduced pressure, and the residue was solubilized in DMSO-$d_6$. Dimethyl sulfone ($Me_2SO_2$, 7 mM-17 mM in DMSO-$d_6$) has been used as internal standard. The quantification has been performed by quantitative $^1$H-NMR spectroscopy (D1 = 30 s, 8 scans) though integration of the signal of $NH_4Cl$ relative to that of $Me_2SO_2$ (δ 3.0 ppm).

## 2. $^1$H NMR spectroscopy

### 2.a. NMR spectra of [(Cp$^{ttt}_2$U)$_2$(μ-N$_2$)] (2)

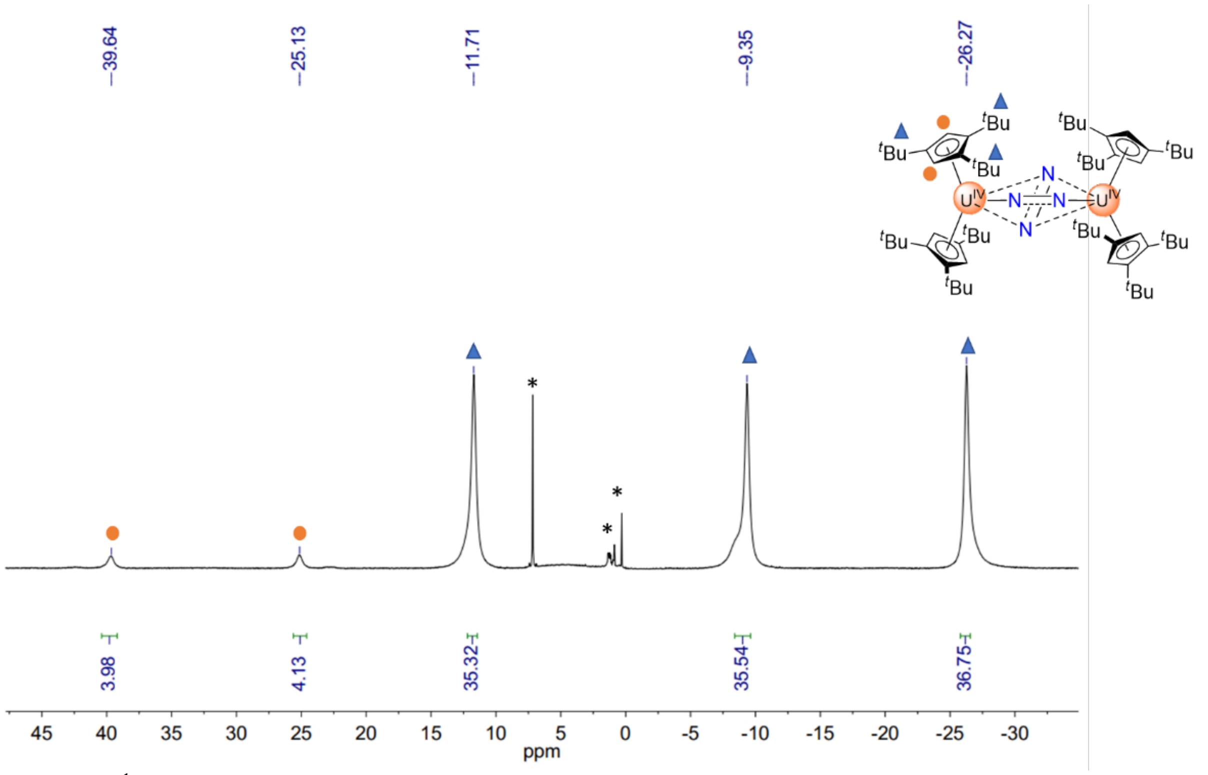


**Figure S2.** $^1$H NMR spectrum of **2** in $C_6D_6$ at 25 °C. Rotation about the U-Cp axes is restricted, resulting in inequivalence of the three $^t$Bu environments.[4] An asterisk (*) marks residual $C_6H_6$ and traces of grease and HCp$^{ttt}$.

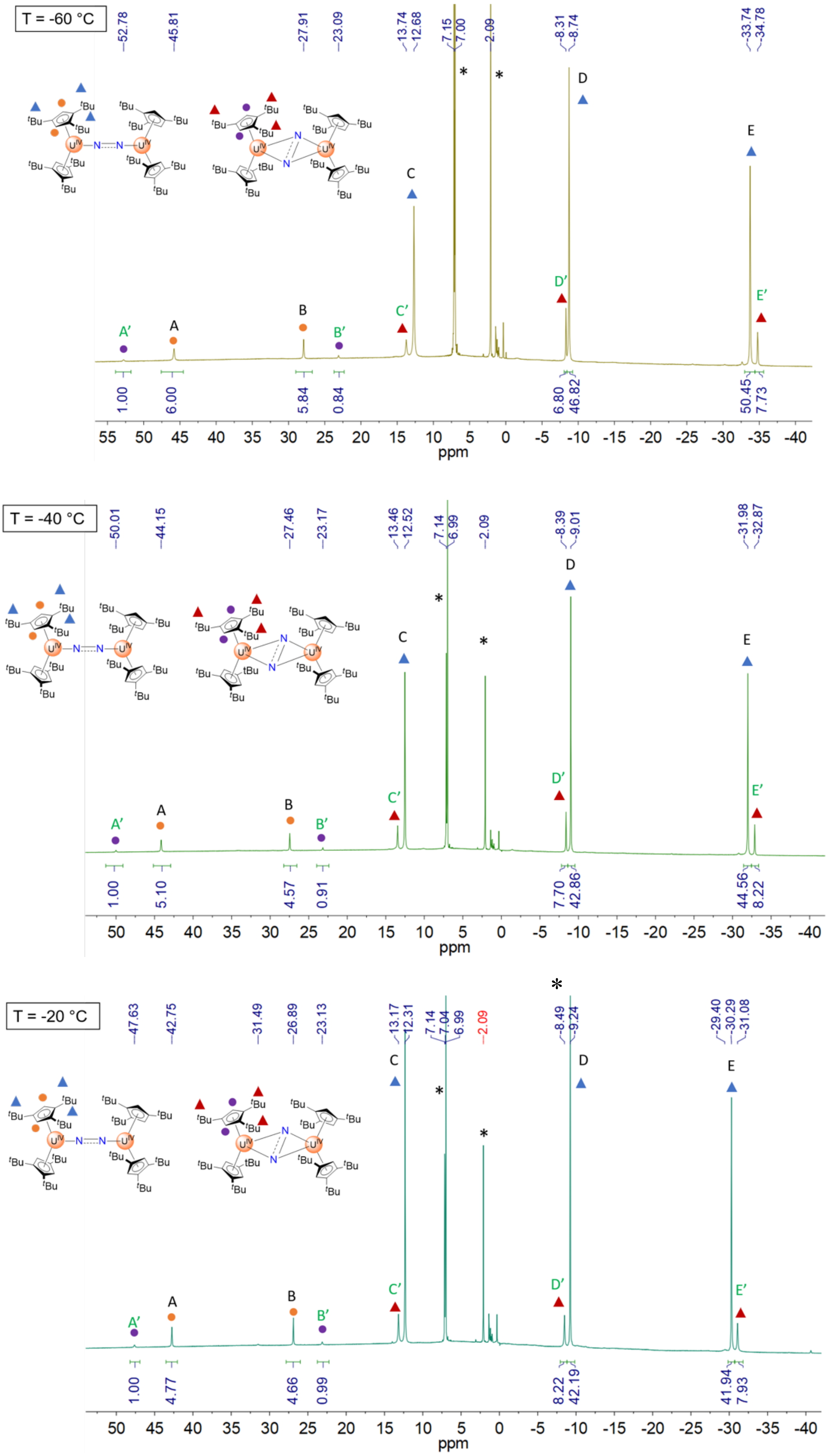


**Figure S3.** $^1$H NMR spectrum of **2** in toluene-$d_8$ at -60, -40, and -20 °C showing both coordination modes of $N_2$. An asterisk (*) marks residual toluene and traces of grease and HCp$^{ttt}$. Lettering represents signal assignments for chemical shift versus 1/T plots (Figures S5-S6).

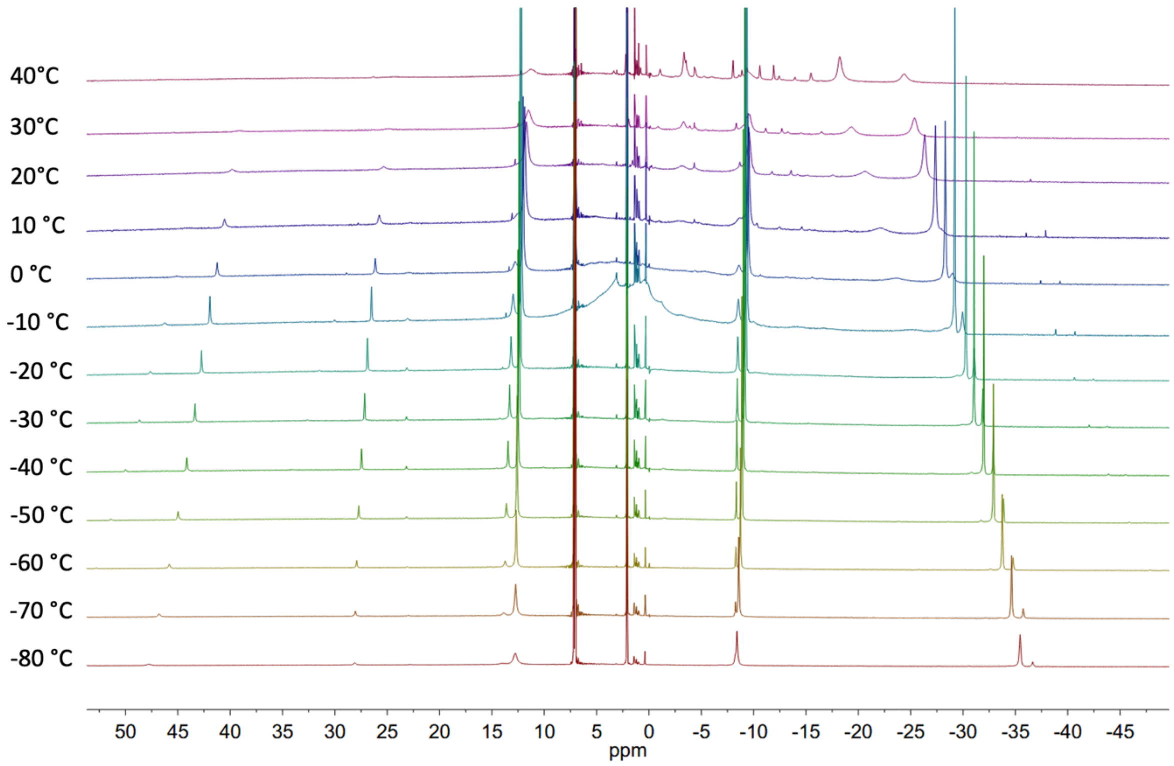


**Figure S4.** Variable temperature $^{1}$H NMR spectra of **2** in toluene-$d_8$.

## 2.b. Temperature dependency of chemical shift signals for NMR signals of 2-end-on

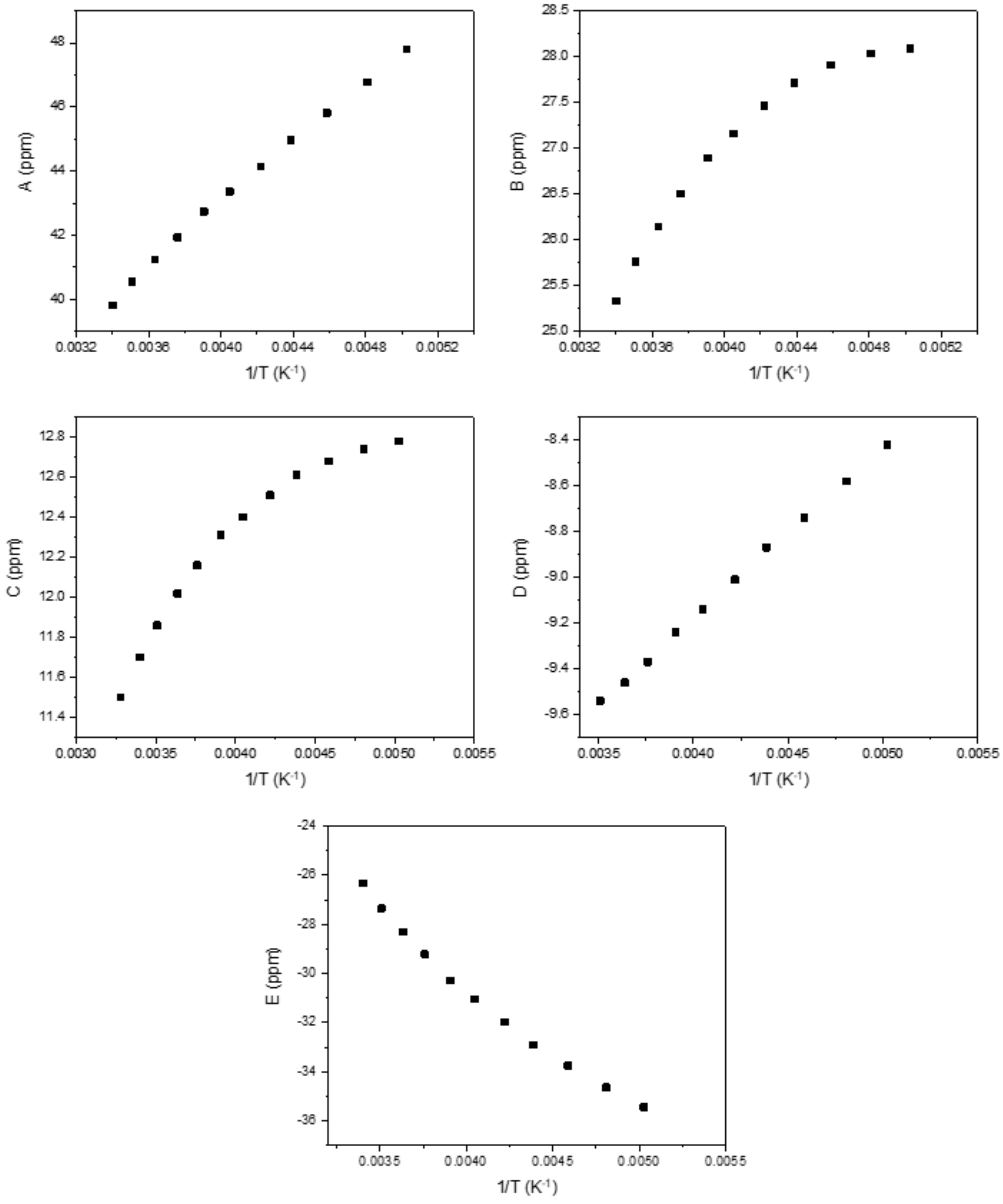


**Figure S5.** Chemical shift (δ) vs 1/T plots of **2-end-on**.

## 2.c. Temperature dependency of chemical shift signals for NMR signals of 2-side-on

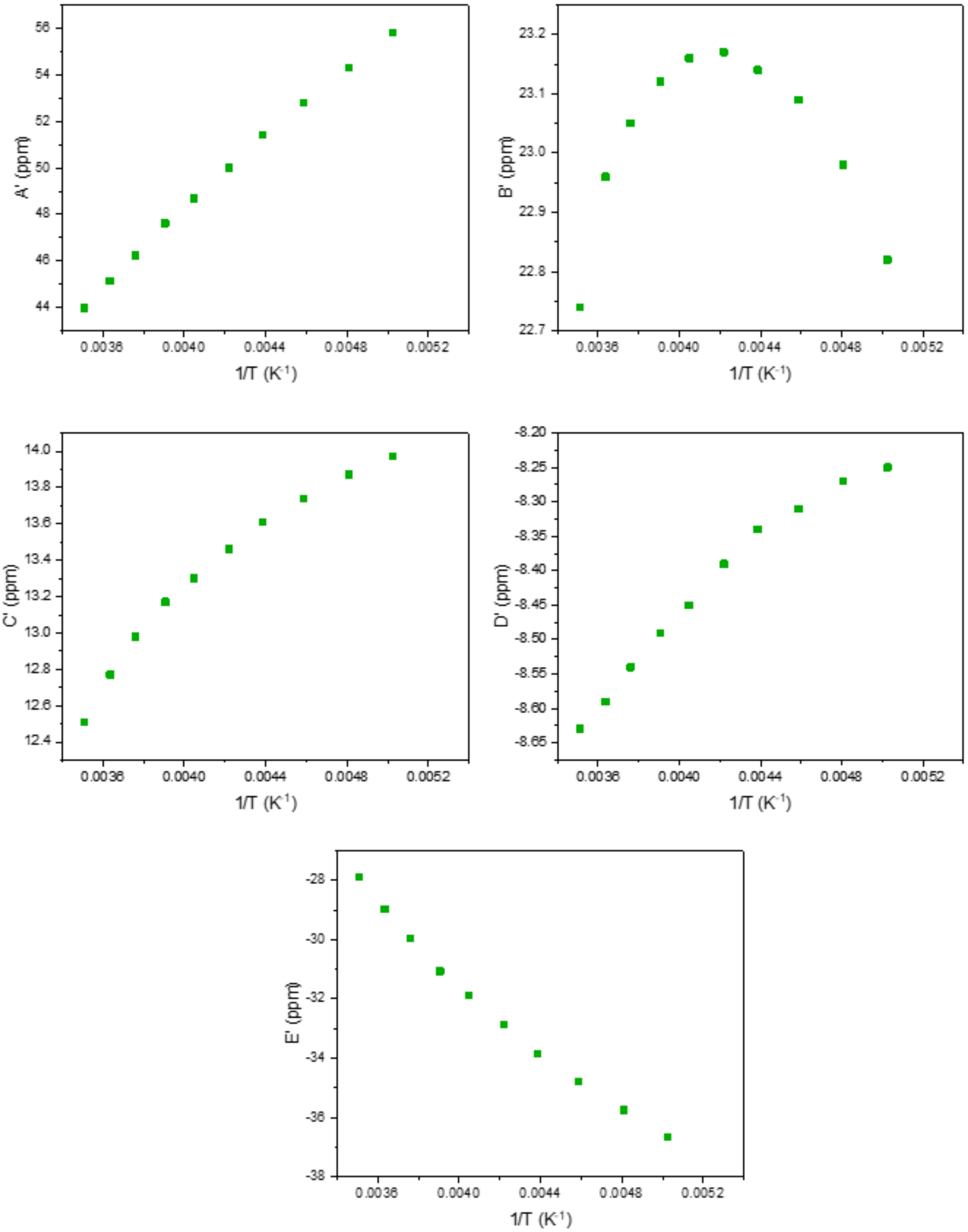


**Figure S6.** Chemical shift (δ) vs 1/T plots of **2-side-on**.

### 2.d. Degradation experiments for $[(Cp^{ttt}{}_2U)_2(\mu\text{-}N_2)]$ (2)

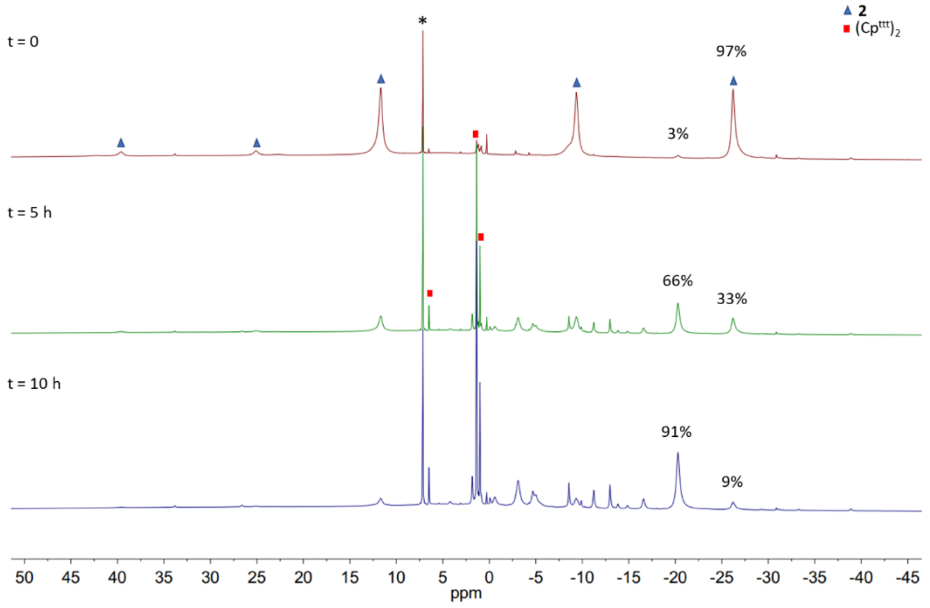


**Figure S7.** Time-dependent degradation of a solution of **2** in benzene-$d_6$, obtained after filtration of the reaction of **1** with 10 eq. of $KC_8$, under 1 atm of $N_2$ at 25 °C. An asterisk (*) marks residual benzene. Blue triangles are signals attributed to **2**, and red squares mark $Cp^{ttt}{}_2$.[5]

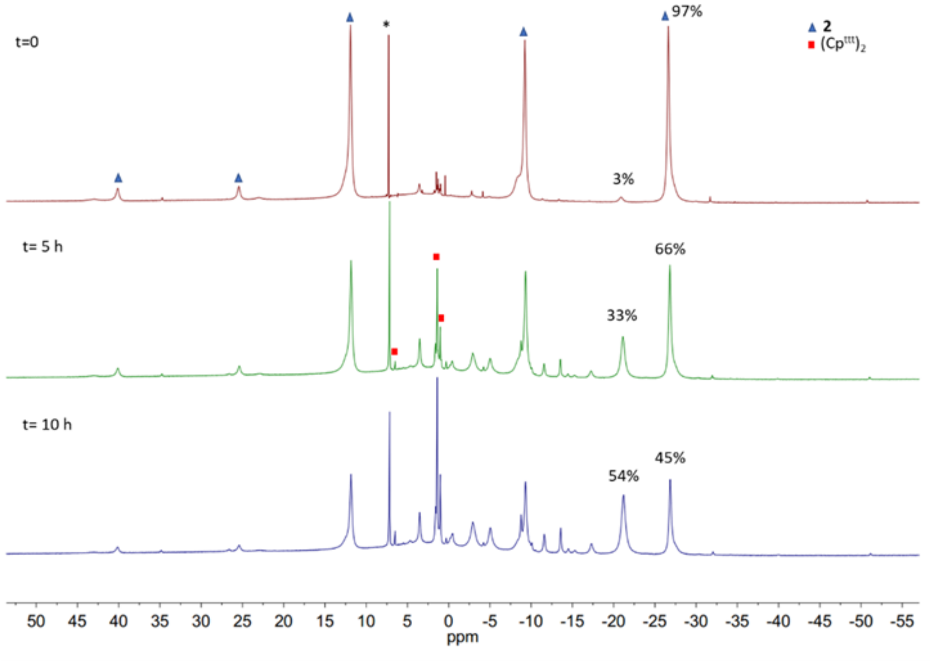


**Figure S8.** Time-dependent degradation of a degassed solution of **2** in benzene-$d_6$, obtained after filtration of the reaction of **1** with 10 eq. of $KC_8$, held under static vacuum ($1{\cdot}10^{-5}$ bar) at 25 °C. An asterisk (*) marks residual benzene and traces of grease and $HCp^{ttt}$. Blue triangles are signals attributed to **2**, and red squares mark $Cp^{ttt}{}_2$.[5]

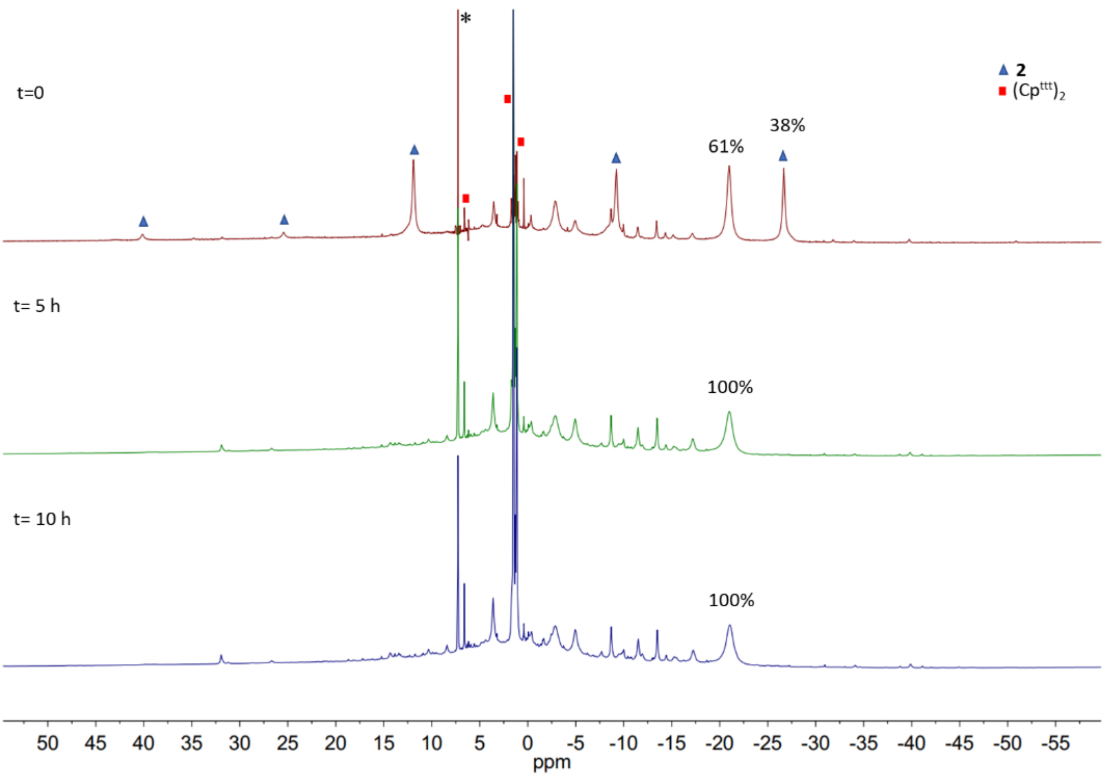


**Figure S9.** Time-dependent degradation of a solution of **2** in benzene-$d_6$, obtained after filtration of the reaction of **1** with 10 eq. of $KC_8$, under 5 bars of $N_2$ at 25 °C. An asterisk (*) marks residual benzene and traces of grease and $HCp^{ttt}$. Blue triangles are signals attributed to **2**, and red squares mark $Cp^{ttt}{}_2$.[5]

**Table S1.** Degradation of **2** at different times in benzene-$d_6$ depending on the $N_2$ pressure. Values are given as % of remaining **2** compared to the major degradation product (see Figures S7-S9).

| Time/pressure | $1 \cdot 10^{-5}$ bar | 1 bar | 5 bars |
|---|---|---|---|
| 0 h | 97 | 97 | 38 |
| 5 h | 66 | 33 | 0 |
| 10 h | 45 | 9 | 0 |

## 2.e. NMR spectra for the protonolysis of [(Cp$^{ttt}{}_2$U)$_2$(μ-N$_2$)] (2)

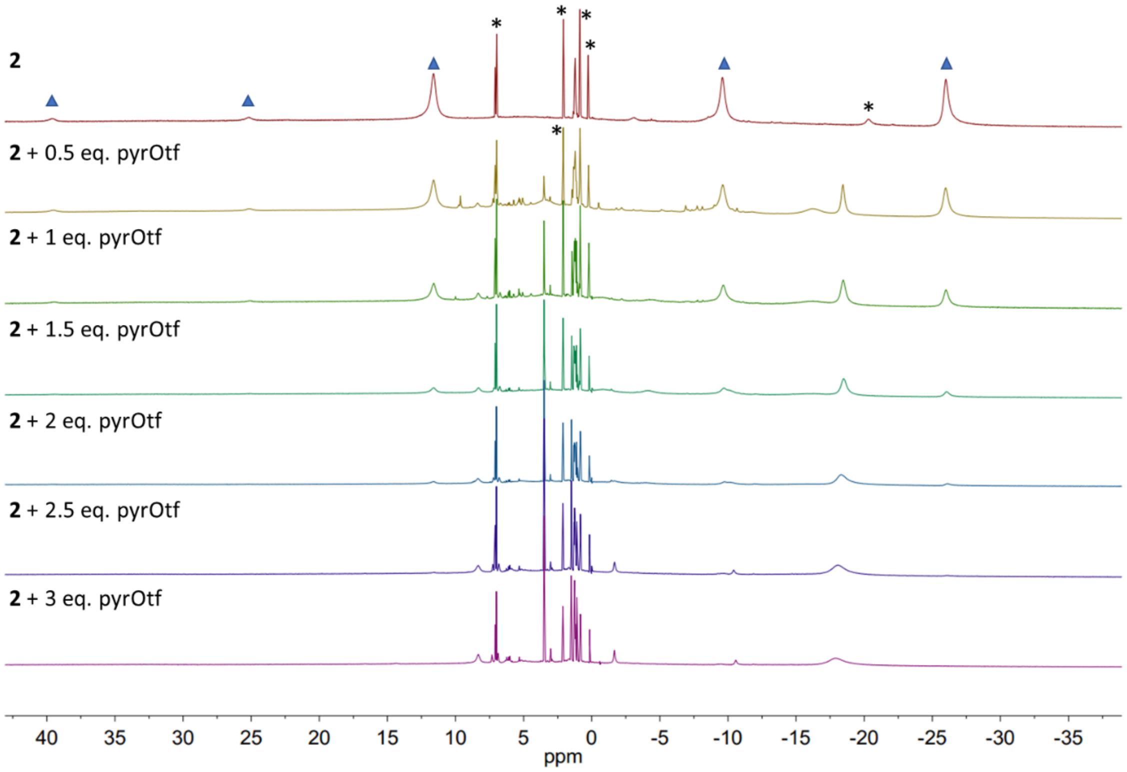


**Figure S10.** $^1$H NMR spectra of **2** in toluene-$d_8$ with incremental addition of pyridinium triflate (in THF-$d_8$). An asterisk (*) marks residual solvents.

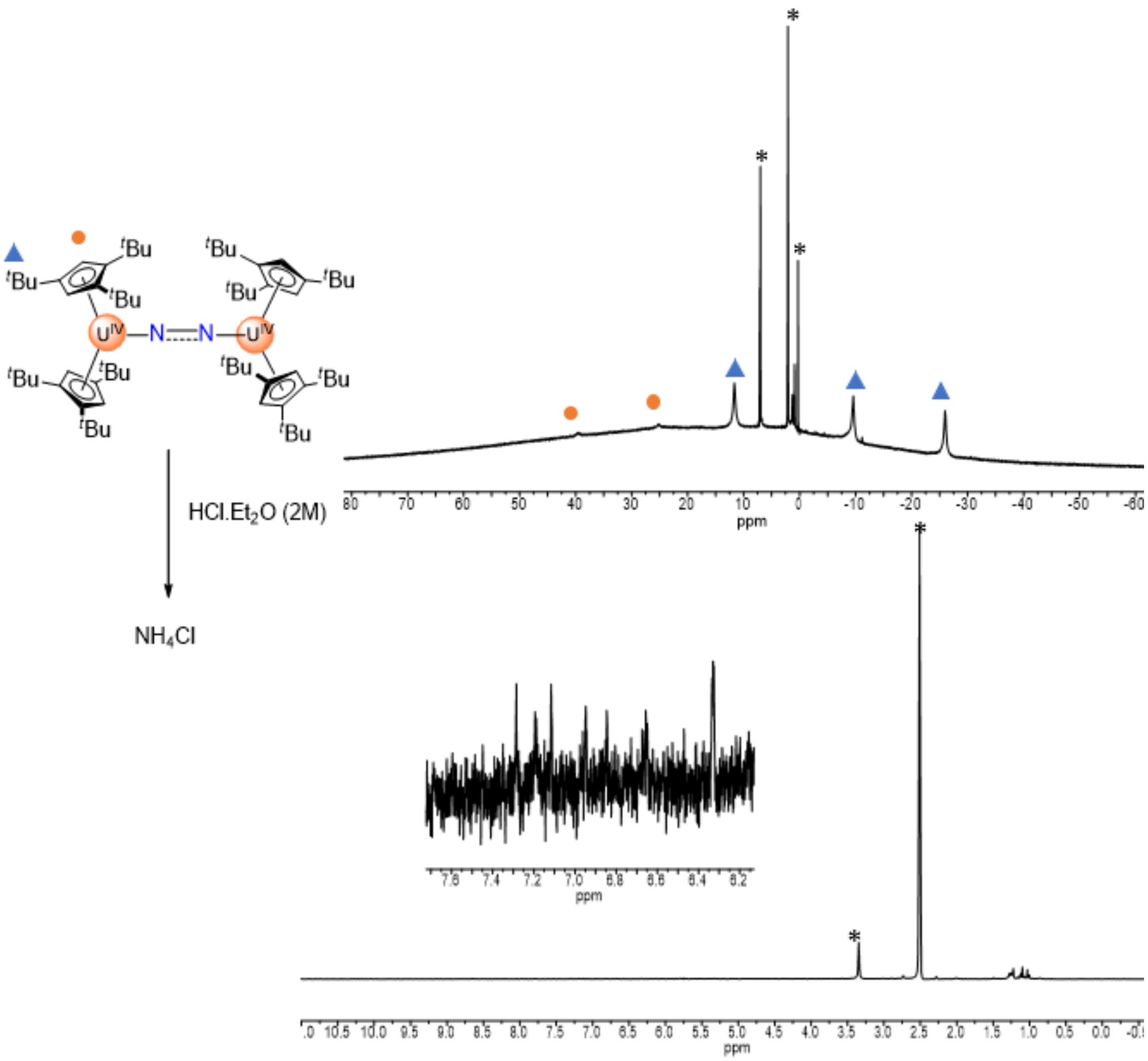


**Figure S11.** $^{1}$H NMR stacked spectra of **2** (top) and after addition HCl($Et_2O$) (2 M) on **2** (bottom) in DMSO-$d_6$. An asterisk (*) marks residual DMSO and water present in the deuterated solvent. No $NH_4^+$ signals were detected by in the $^{1}$H NMR spectrum.

2.f. Hydrogenation of $[(Cp^{ttt}_2U)_2(\mu\text{-}N_2)]$ (2) and protonolysis

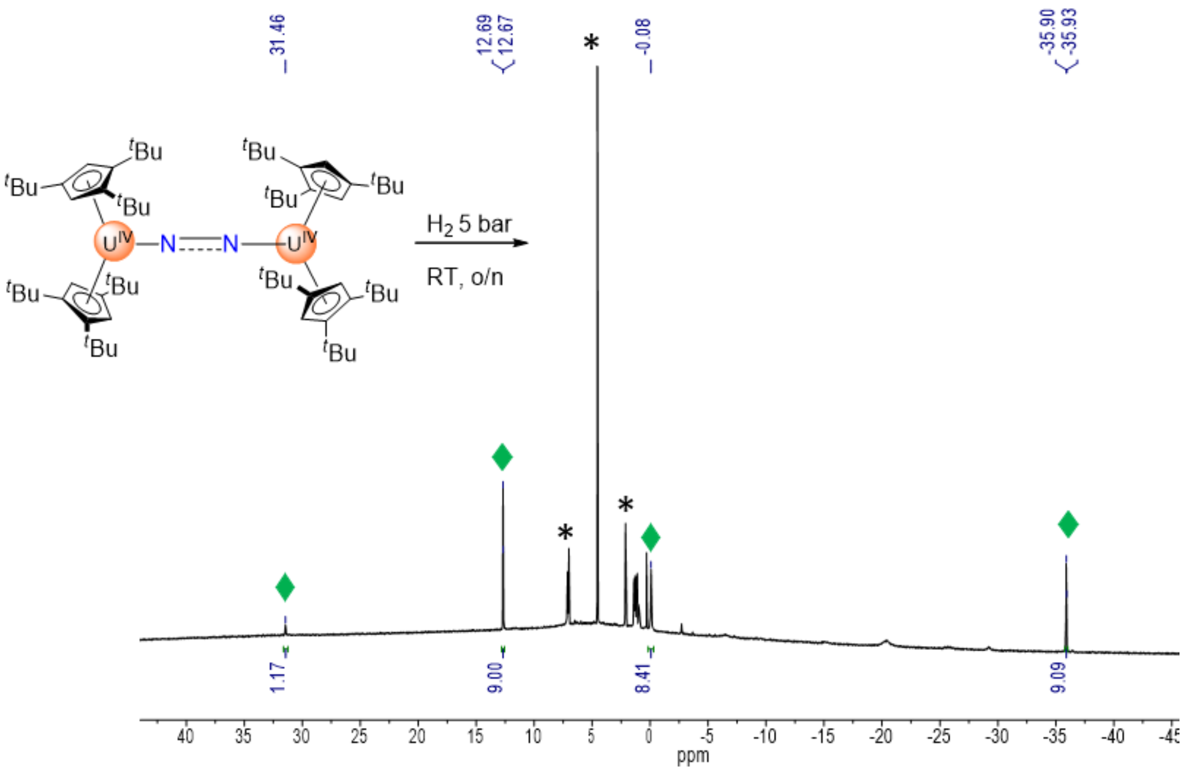


**Figure S12.** $^{1}$H NMR spectrum of **2** after addition of $H_2$ (5 bars) in toluene-$d_8$. An asterisk (*) marks residual solvents and gas ($H_2$) from the deuterated solvent. The green diamonds represent the main product of the reaction, attributed to compound **A**.

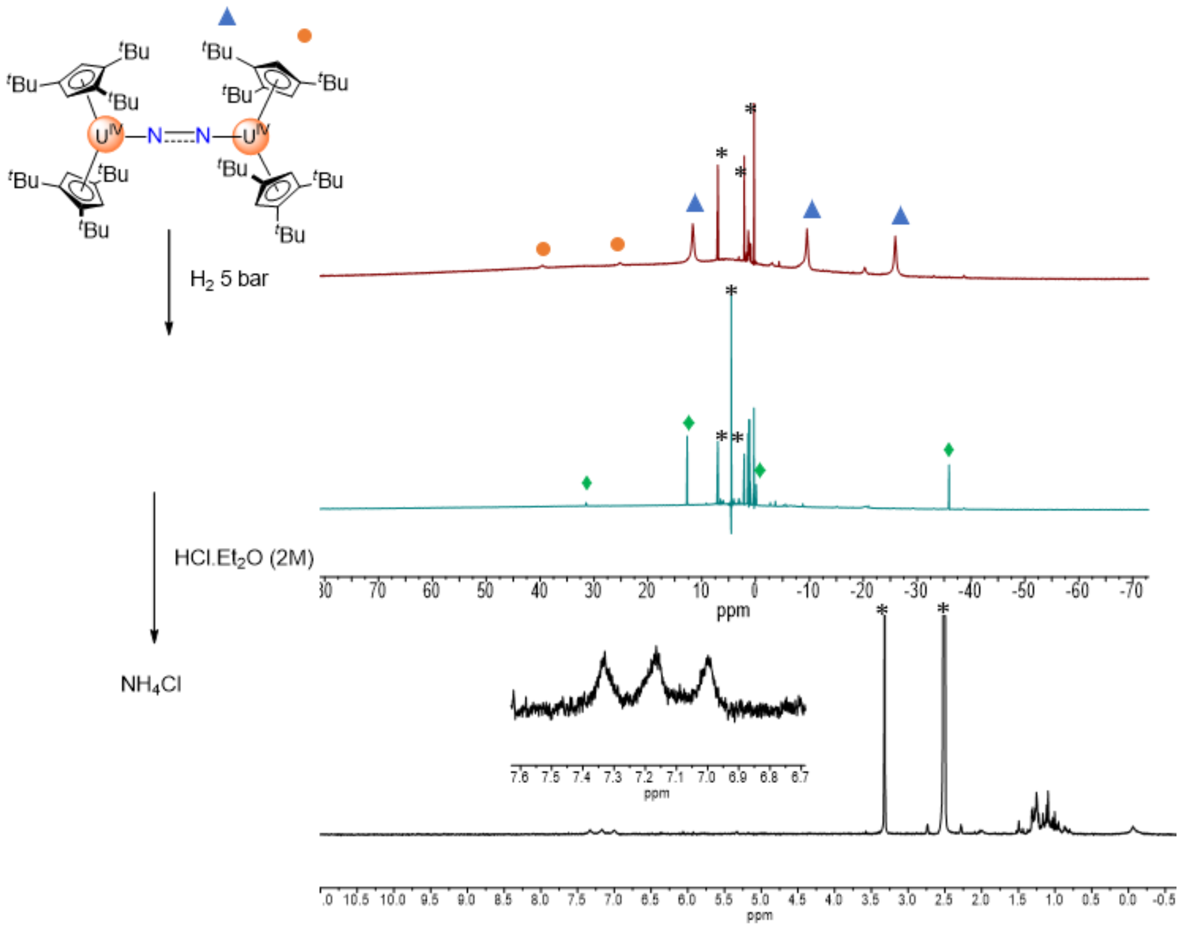


**Figure S13.** $^{1}$H NMR stacked spectra of **2** (top), after the addition of $H_2$ (middle) in toluene-$d_8$ and after the addition of HCl($Et_2O$) (2 M) (bottom) in DMSO-$d_6$. An asterisk (*) marks residual solvents and gas ($H_2$) from the deuterated solvent. The green diamonds represent the main product of the reaction.

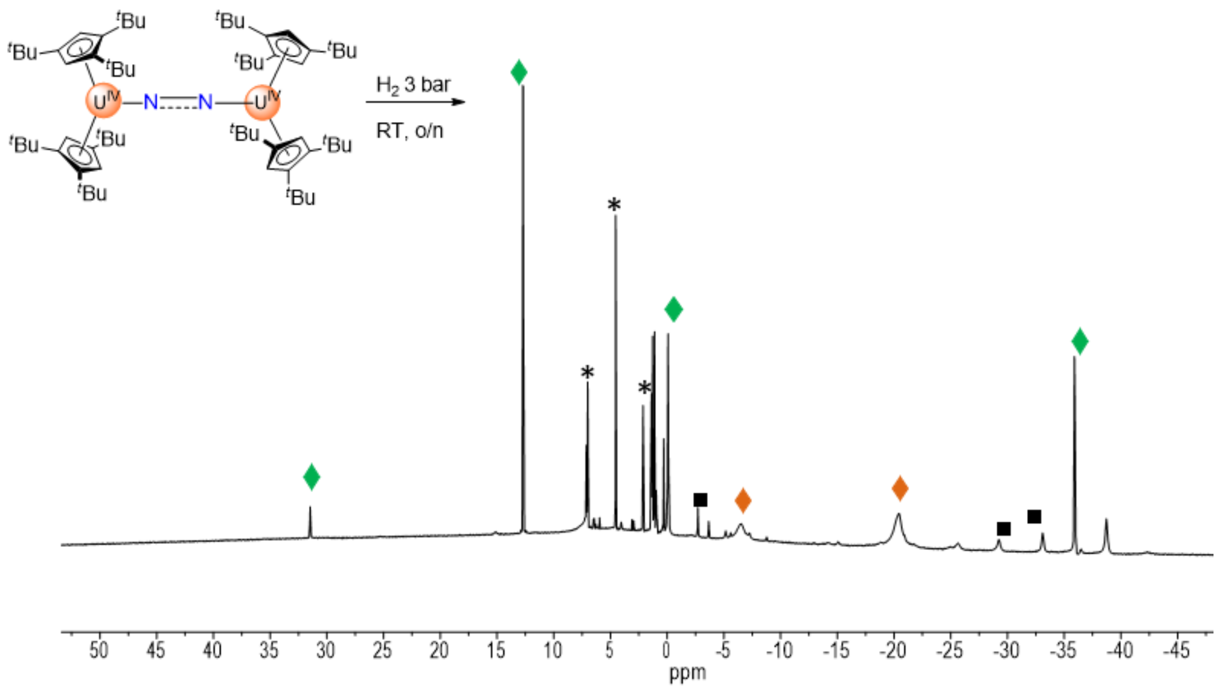


**Figure S14.** $^{1}$H NMR spectrum of **2** after addition of $H_2$ (3 bar) in toluene-$d_8$. An asterisk (*) marks residual solvents and gas ($H_2$) from the deuterated solvent. The green and orange diamonds represent the two main products of the reaction. The black squares represent some unidentified product.

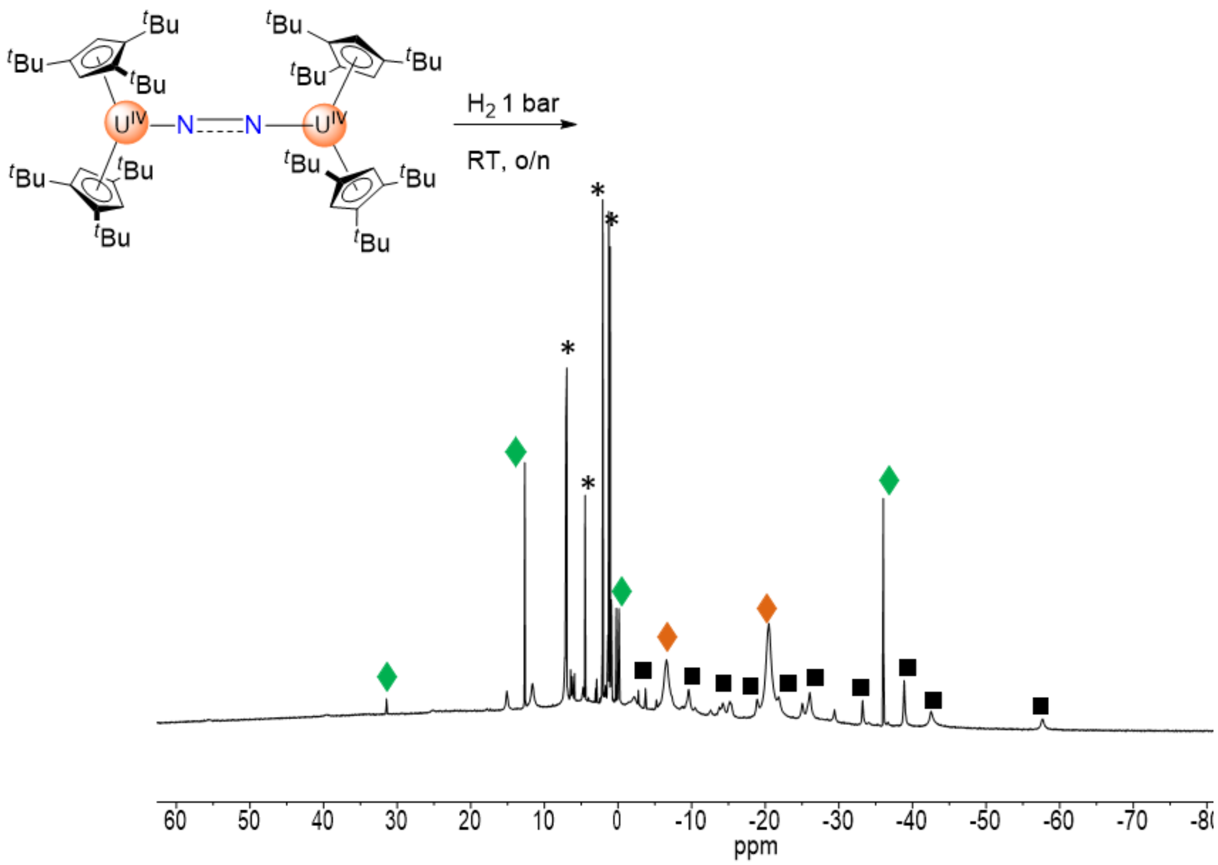


**Figure S15.** $^{1}$H NMR spectrum of **2** after addition of $H_2$ (1 bar) in toluene-$d_8$. An asterisk (*) marks residual solvents and gas ($H_2$) from the deuterated solvent. The green and orange diamonds represent the two main products of the reaction. The black squares represent some unidentified product.

.

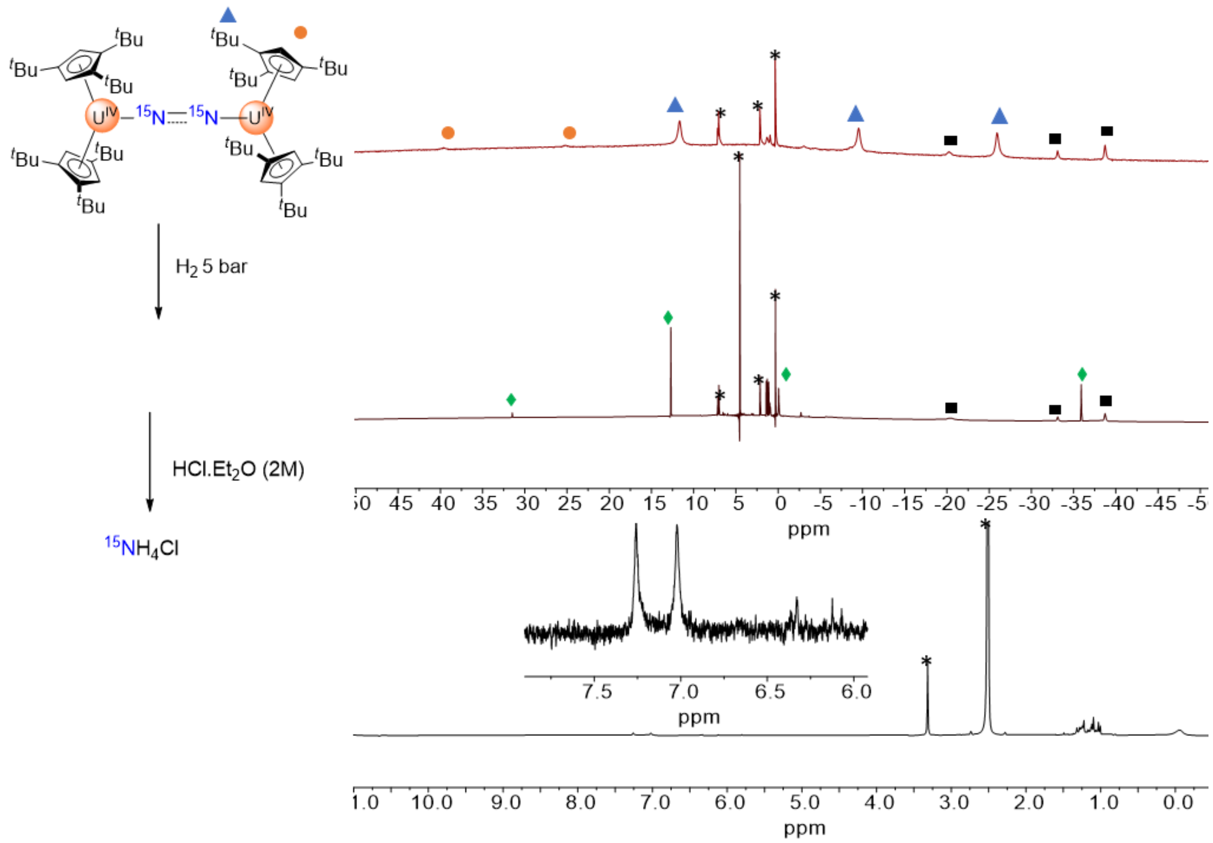


**Figure S16.** $^{1}$H NMR stacked spectra of **2** (top), after the addition of $H_2$ (middle) in toluene-$d_8$ and after the addition of HCl($Et_2O$) (2 M) (bottom) in DMSO-$d_6$. An asterisk (*) marks residual solvents, grease and gas ($H_2$) from the deuterated solvent. The black squares represent an impurity formed in the $^{15}$N-labelled complex**,** and the green diamonds represent compound **A**.

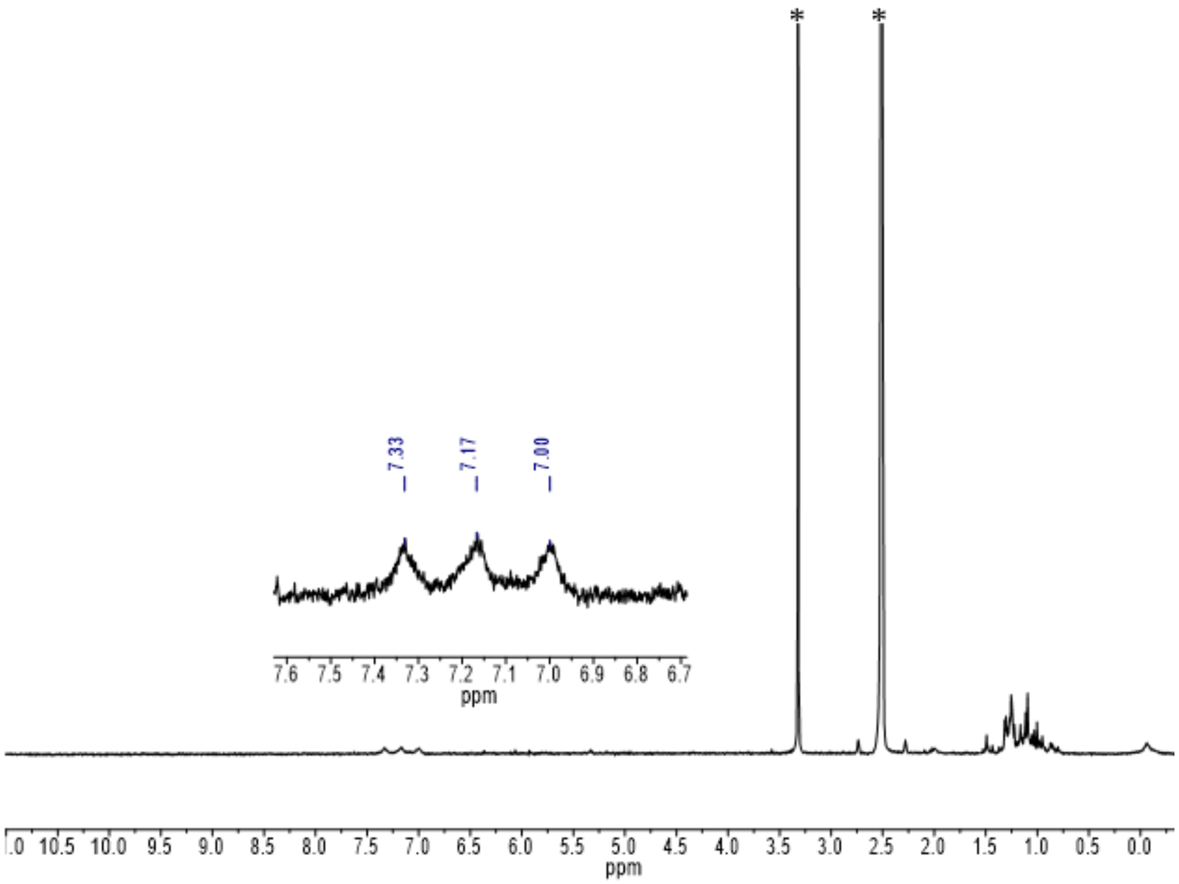


**Figure S17.** $^{1}$H NMR spectrum of $NH_4Cl$ after addition $HCl(Et_2O)$ (2 M) of **2** + $H_2$ (5 bar) in DMSO-$d_6$. An asterisk (*) marks residual solvents present in the deuterated solvent. The 1:1:1 triplet marks the presence of the ammonium with $J$ = 50 Hz.

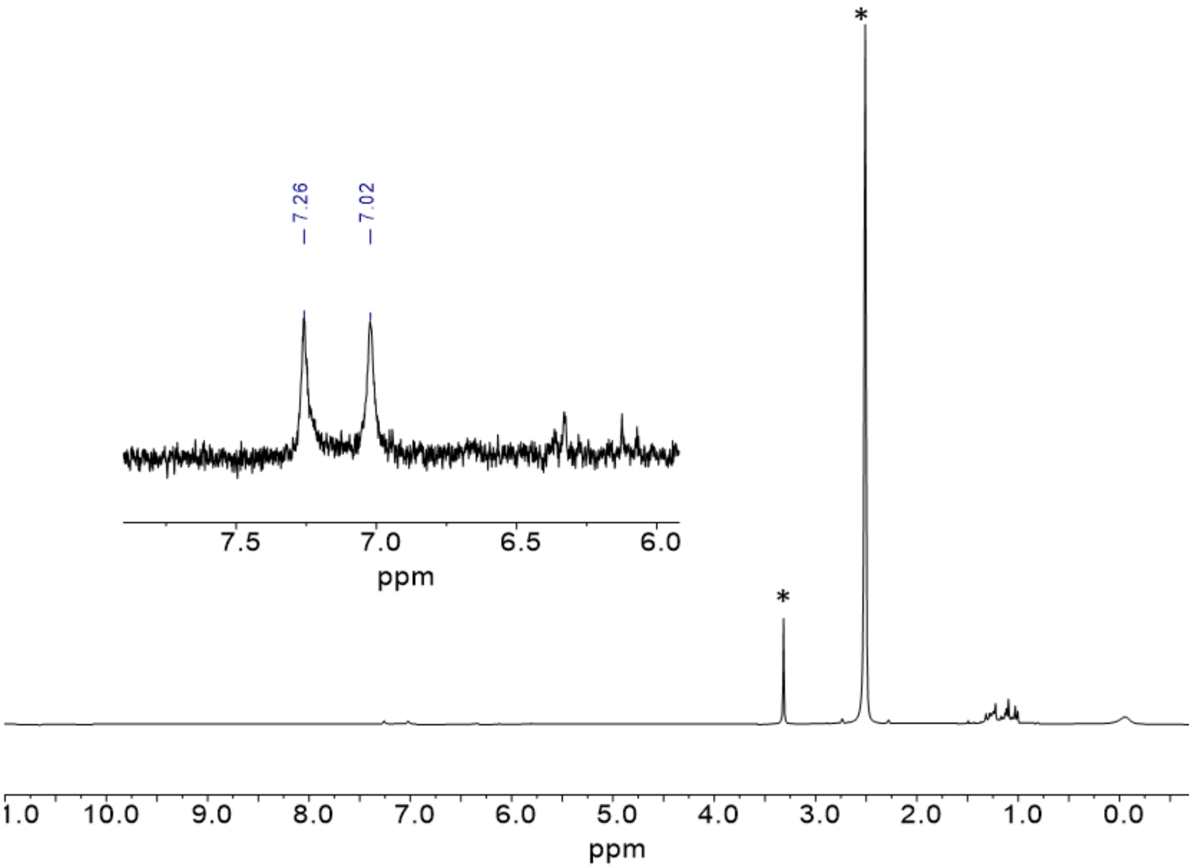


**Figure S18.** $^{1}H$ NMR spectrum of $^{15}NH_4Cl$ after addition HCl($Et_2O$) (2 M) of **2-$^{15}$N** + $H_2$ (5 bar) in DMSO-$d_6$. An asterisk (*) marks residual solvents present in the deuterated solvent. The 1:1 doublet marks the presence of the $^{15}$N-ammonium with $J$ = 71 Hz.

## 2.g. NMR spectra of [$Cp^{ttt}{}_2U(\mu\text{-}I)(\mu_3\text{-}N)(\mu\text{-}N)(UCp^{ttt})_2$] (3)

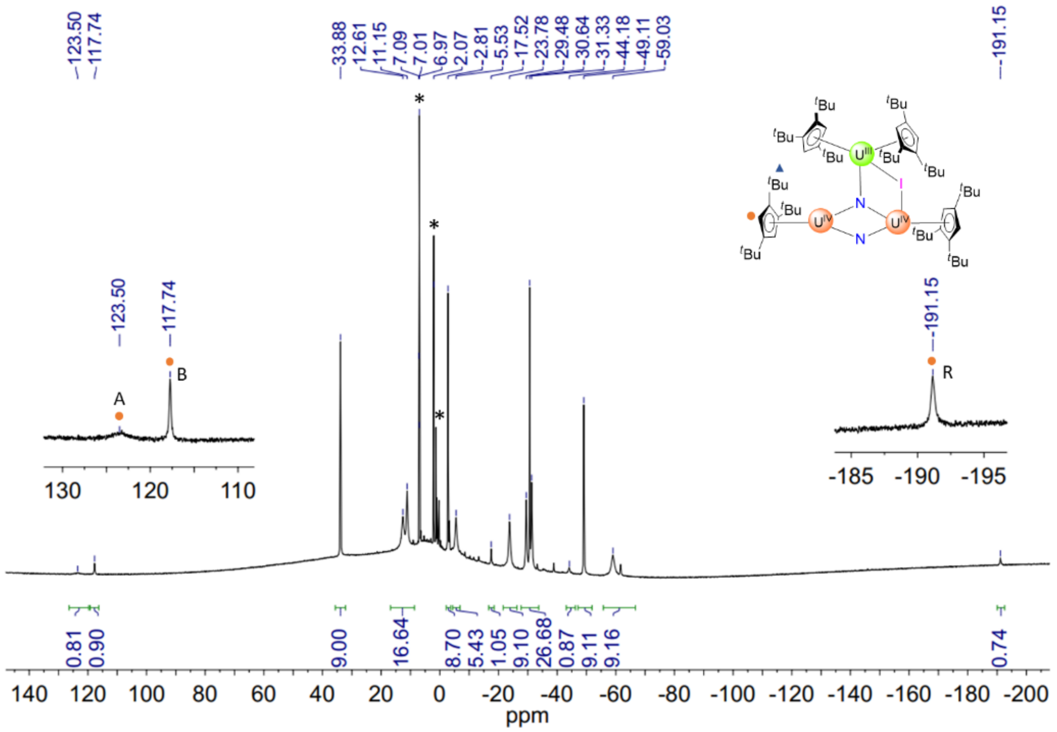


**Figure S19.** $^1$H NMR spectrum of **3** in toluene-$d_8$ at 25 °C. An asterisk (*) marks residual toluene and traces of grease and $HCp^{ttt}$. Lettering represents signal assignments for chemical shift versus 1/T plots (Figures S24-S26).

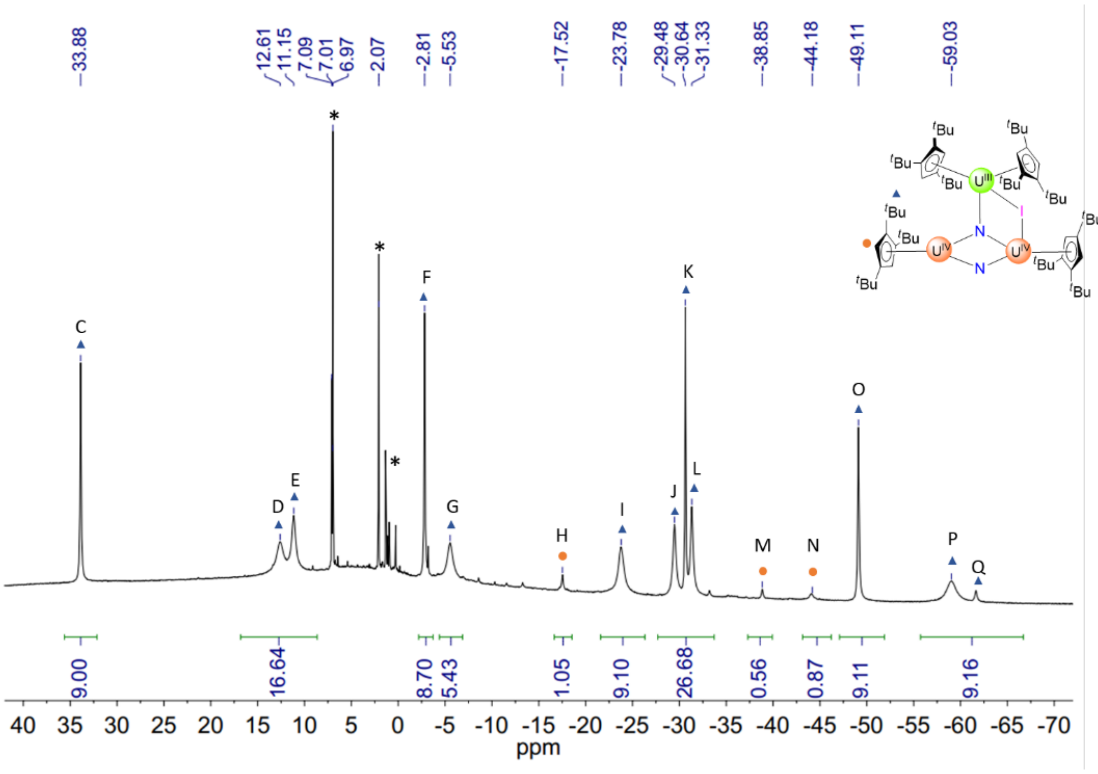


**Figure S20.** Expansion (δ: -70 – 40 ppm) of $^1$H NMR spectrum of **3** in toluene-$d_8$ at 25 °C. An asterisk (*) marks residual toluene and traces of grease and $HCp^{ttt}$.

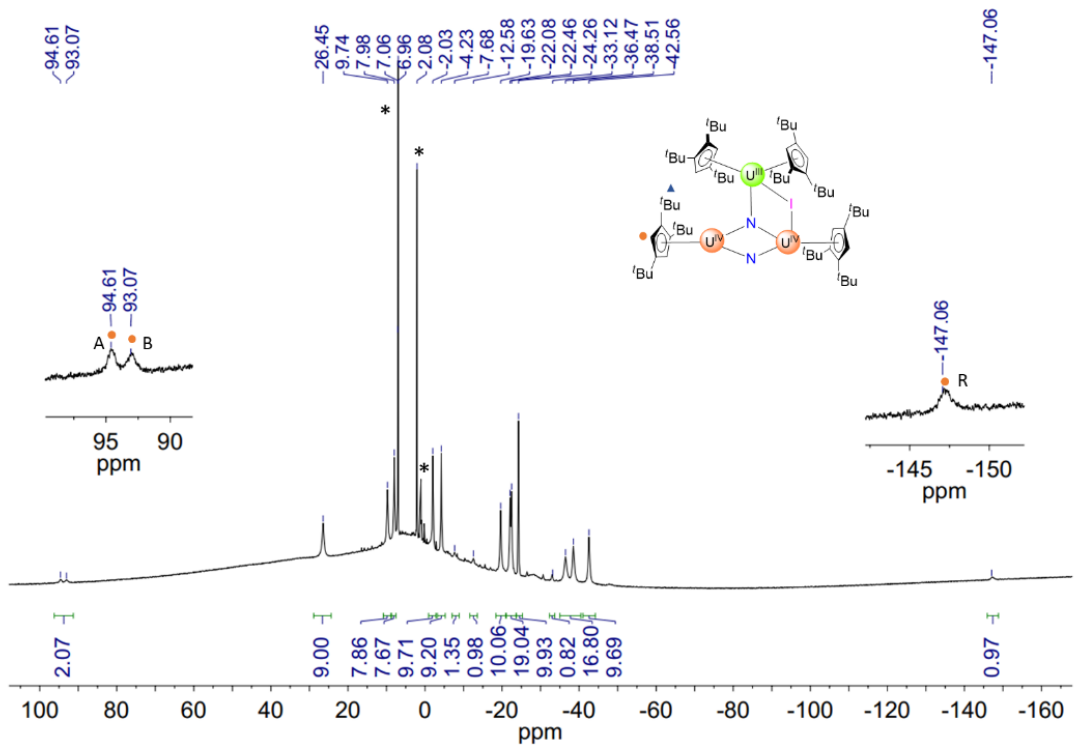

**Figure S21.** $^1$H NMR spectrum of **3** in toluene-$d_8$ at 80 °C. An asterisk (*) marks residual toluene and traces of grease and HCp$^{ttt}$. Lettering represents signal assignments for chemical shift versus 1/T plots (Figures S24-S26).

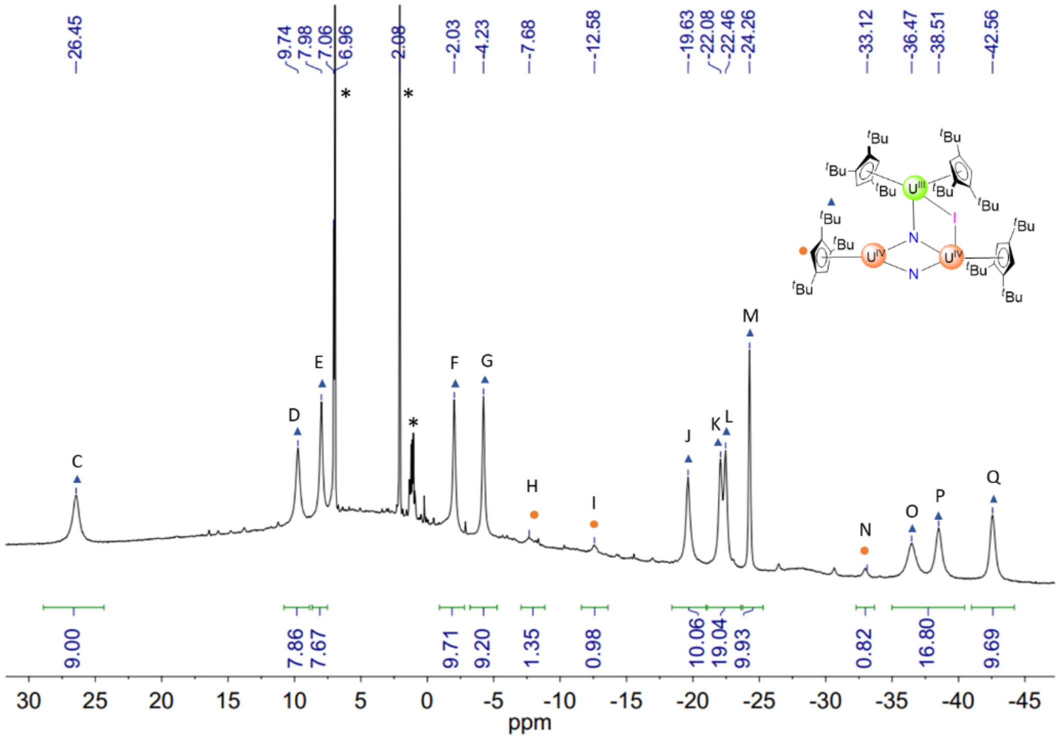

**Figure S22.** Expansion (δ: -45 – 30 ppm) of $^1$H NMR spectrum of **3** in toluene-$d_8$ at 80 °C. An asterisk (*) marks residual toluene and traces of grease and HCp$^{ttt}$.

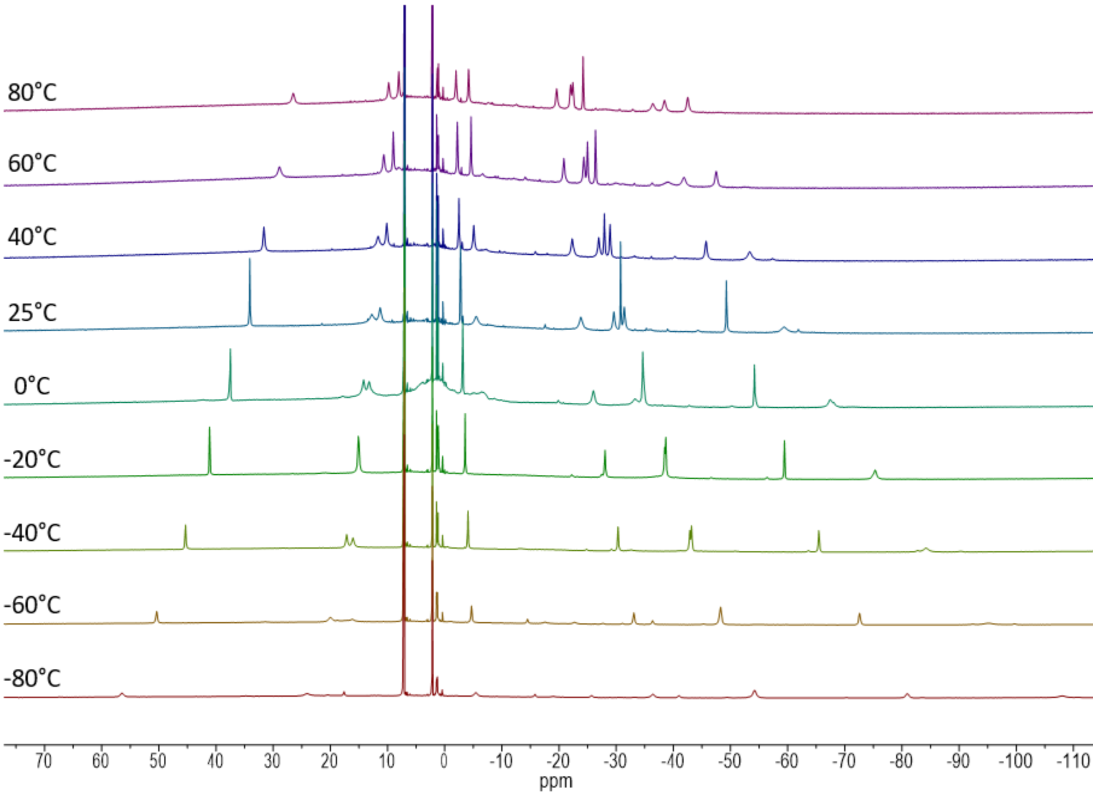


**Figure S23.** Variable temperature $^{1}H$ NMR spectra of **3** in toluene-$d_8$.

## 2.h. Temperature dependency of chemical shift signals for NMR signals of 3

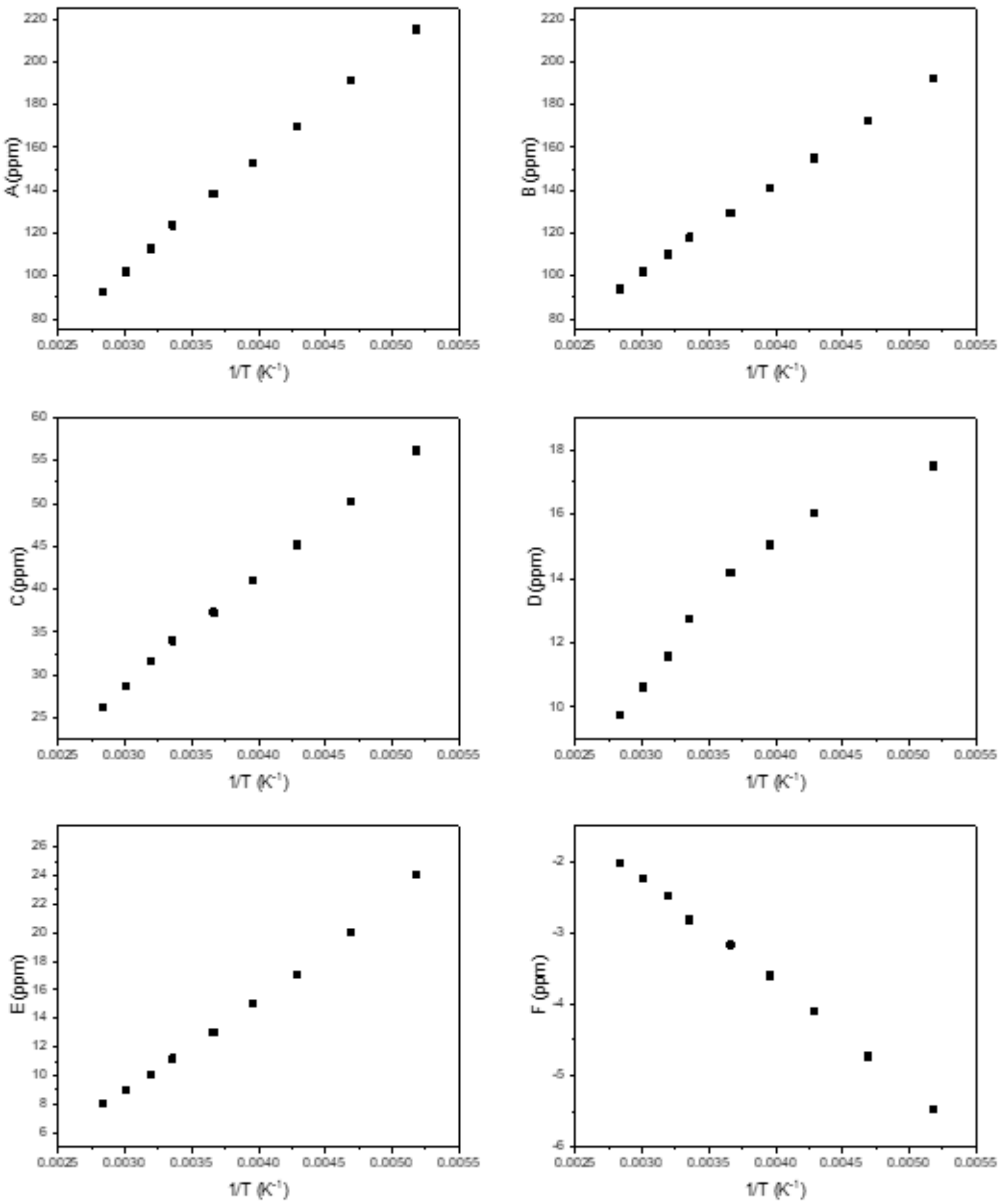


**Figure S24.** Chemical shift (δ) vs 1/T plots of **3**, signals A-F.

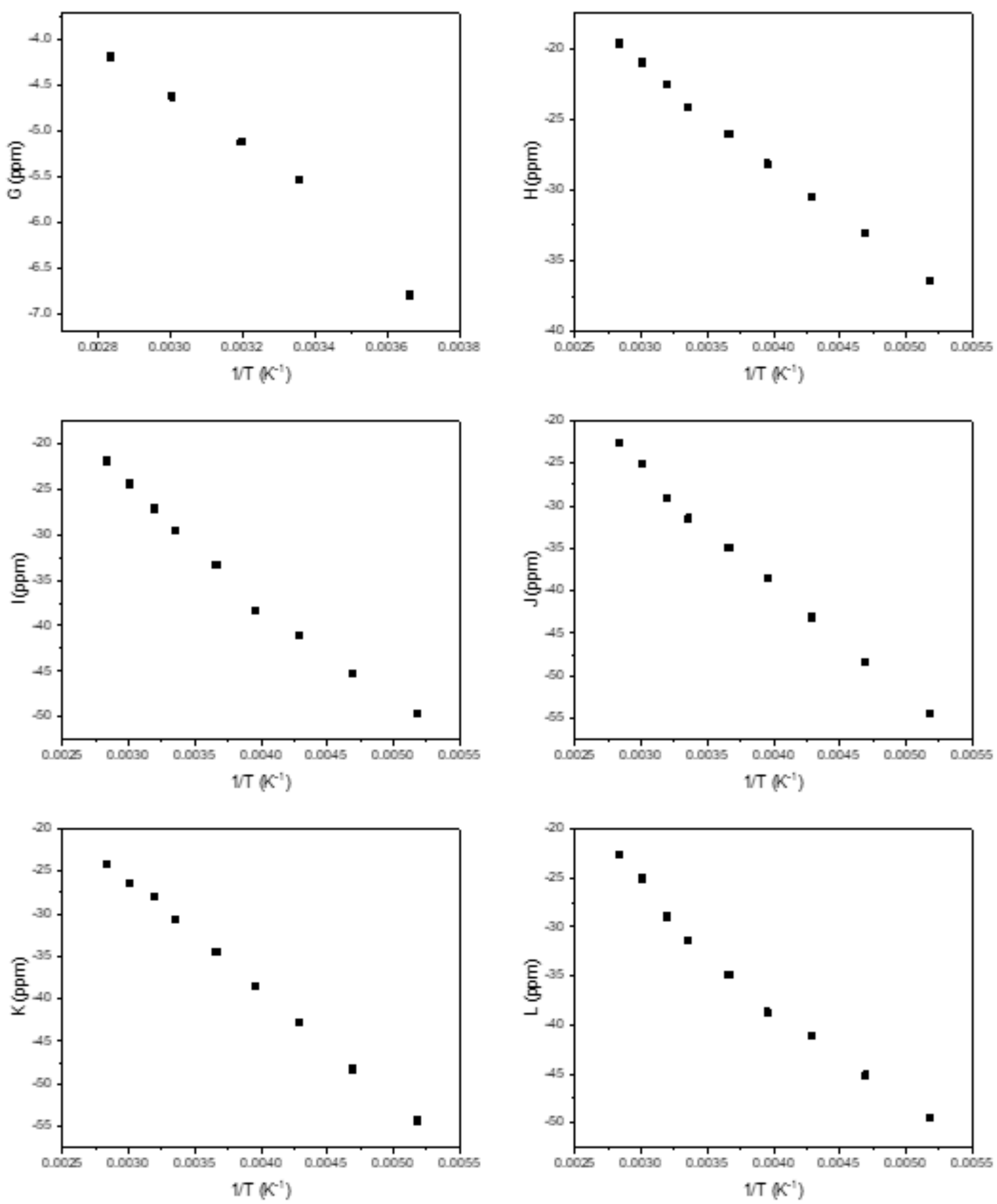


**Figure S25.** Chemical shift (δ) vs 1/T plots of **3**, signals G-L.

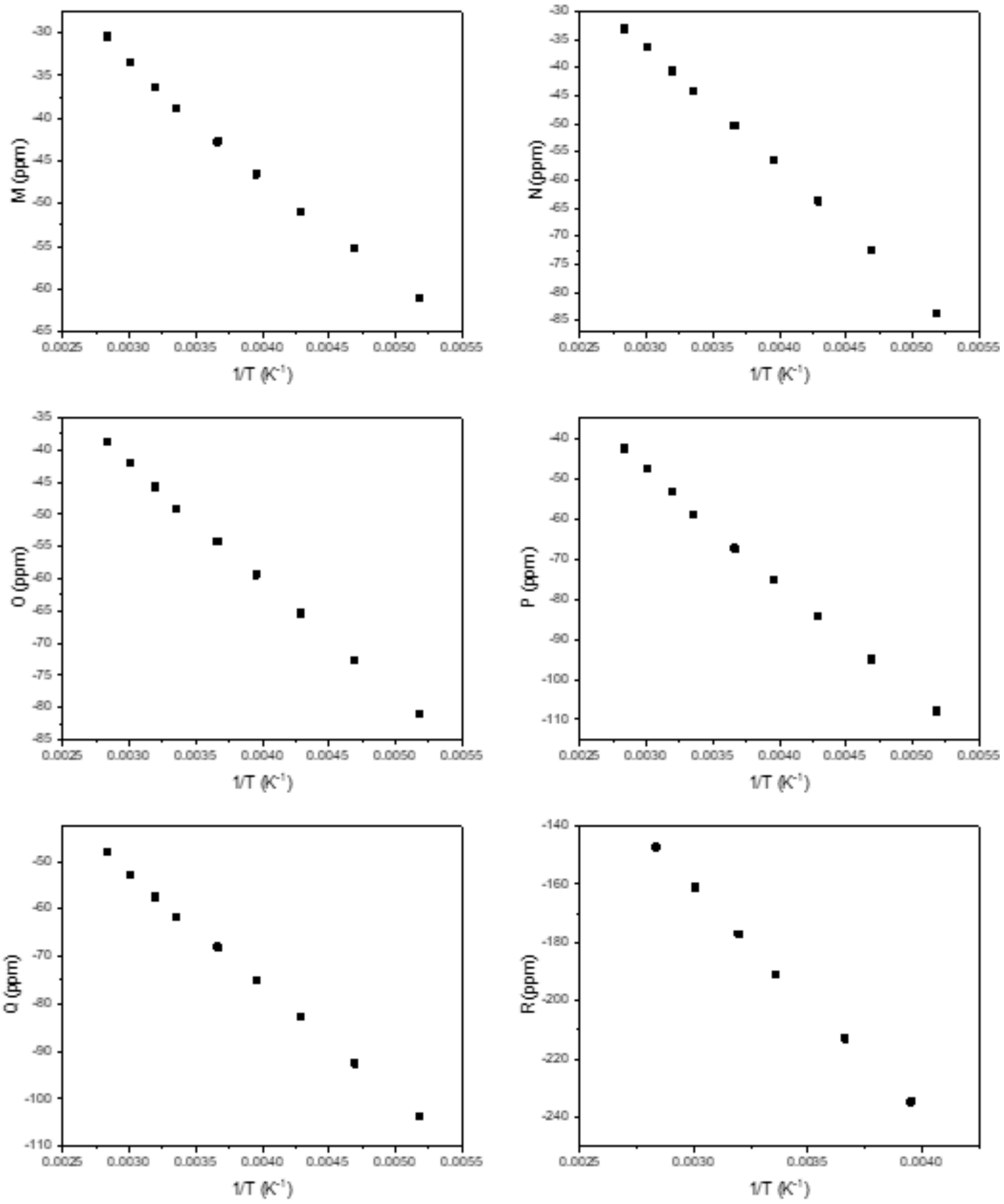


**Figure S26.** Chemical shift (δ) vs 1/T plots of **3**, signals M-R.

## 2.i. NMR spectra for the hydrogenation and protonolysis of 3

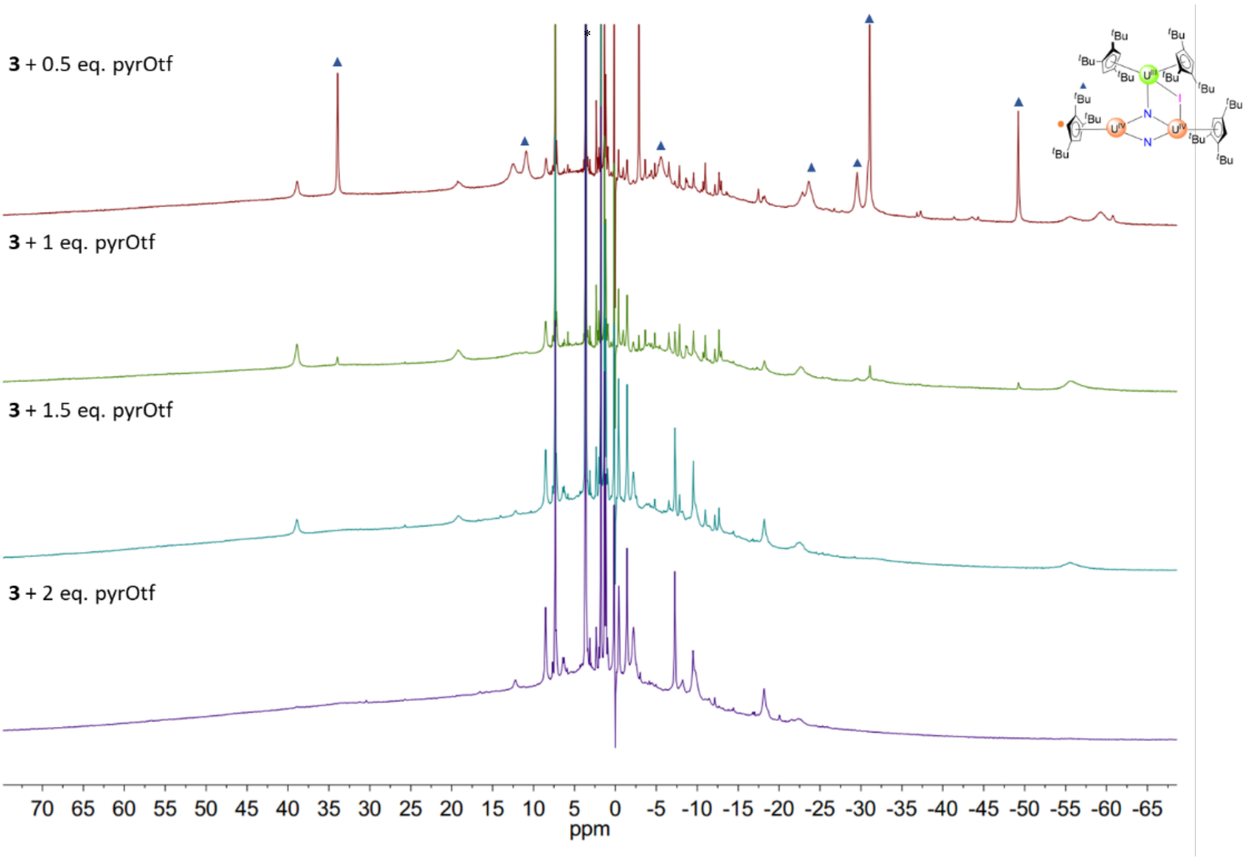


**Figure S27.** $^{1}$H NMR spectra of **3** in THF-$d_8$ with incremental addition of pyridinium triflate. An asterisk (*) marks residual THF.

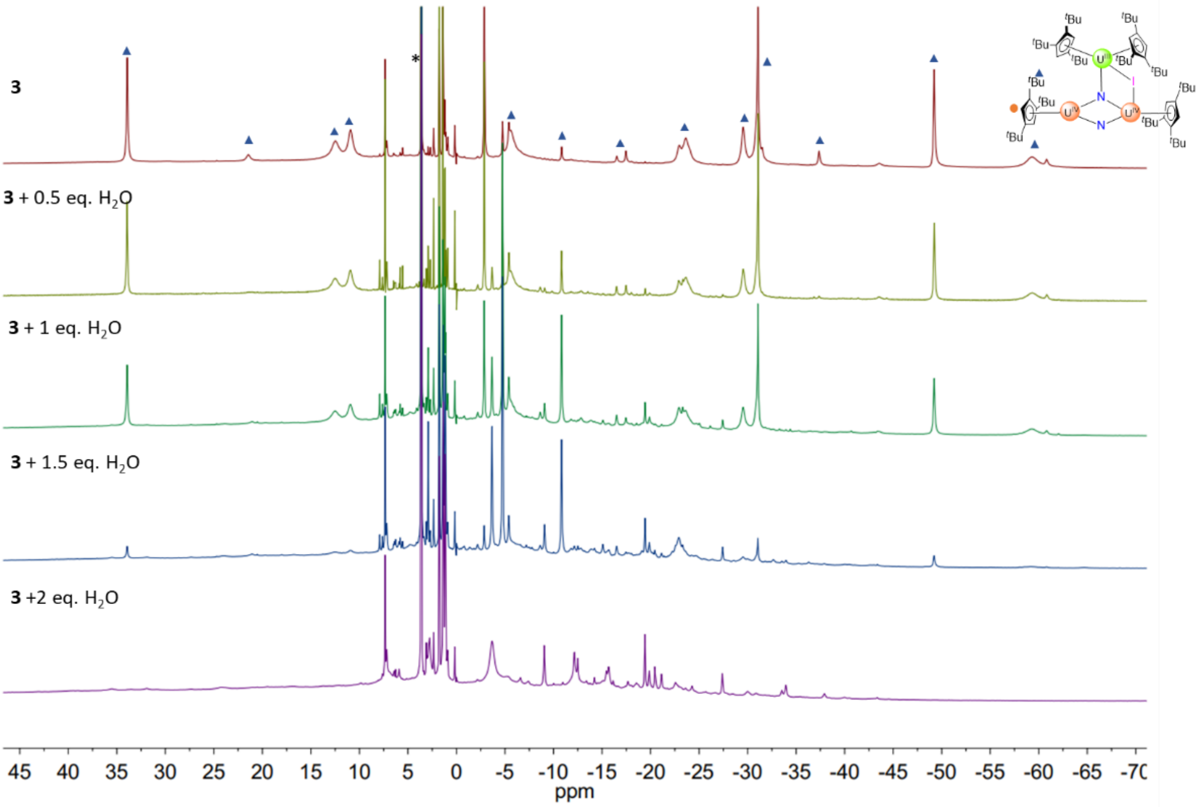


**Figure S28.** $^1H$ NMR spectra of **3** in THF-$d_8$ with incremental addition of $H_2O$. An asterisk (*) marks residual THF.

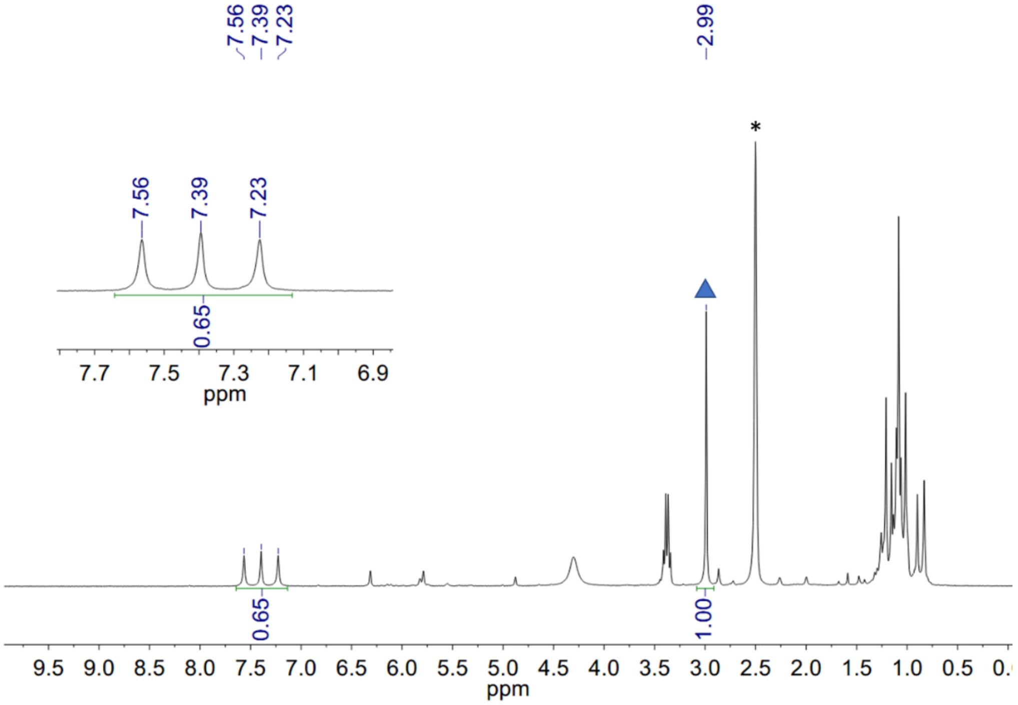


**Figure S29.** $^1H$ NMR of **3** (5.5 µmol) after addition of excess $HCl(Et_2O)$ (2M) (100 µL, 200 µmol) in DMSO-$d_6$ (508 µL) at 25 °C showing the formation of $NH_4Cl$. The blue triangle marks the signals of $Me_2SO_2$ (17 mM) used as an internal standard for the quantification of $NH_4Cl$ (8.74 µmol, yield: 69%). An asterisk (*) marks the residual DMSO-$d_6$.

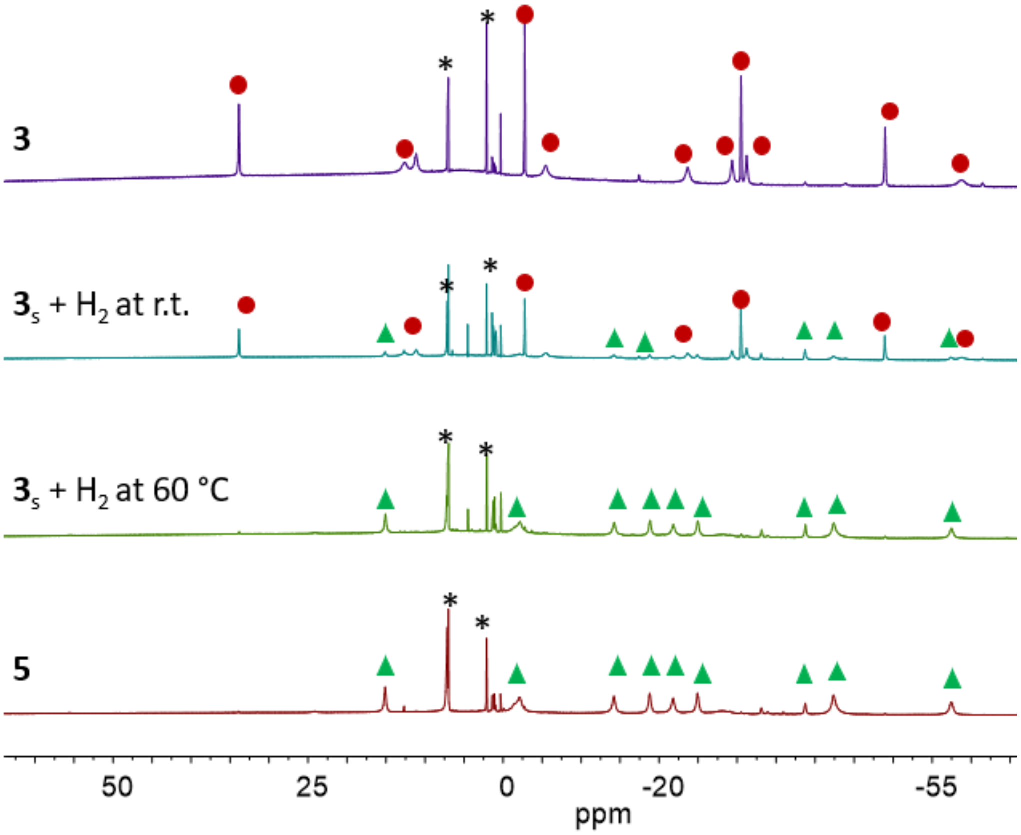


**Figure S30.** $^{1}$H NMR stacked spectra of **3** (top) solid-state hydrogenation of **3** with the addition $H_2$ (1 atm) at room temperature overnight, and after heating at 60 °C overnight (middle), and **5** in toluene-$d_8$ (bottom). The red circles and green triangles represent **3** and **5**, respectively. An asterisk (*) marks residual toluene.

## 2.j. NMR spectra of [Cp$^{ttt}_2$U(μ-N)$_2${U(Cp$^{ttt}$)(OEt$_2$)}] (4)

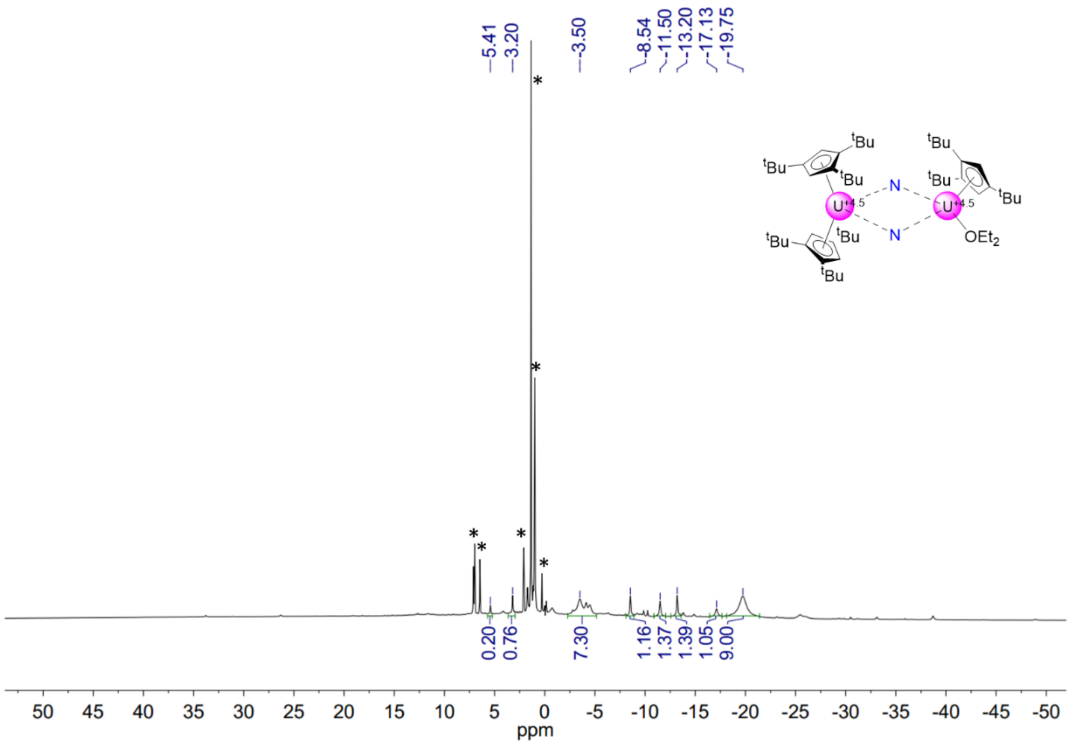


**Figure S31.** $^1$H NMR spectrum of **4** in toluene-$d_8$ at 25 °C.

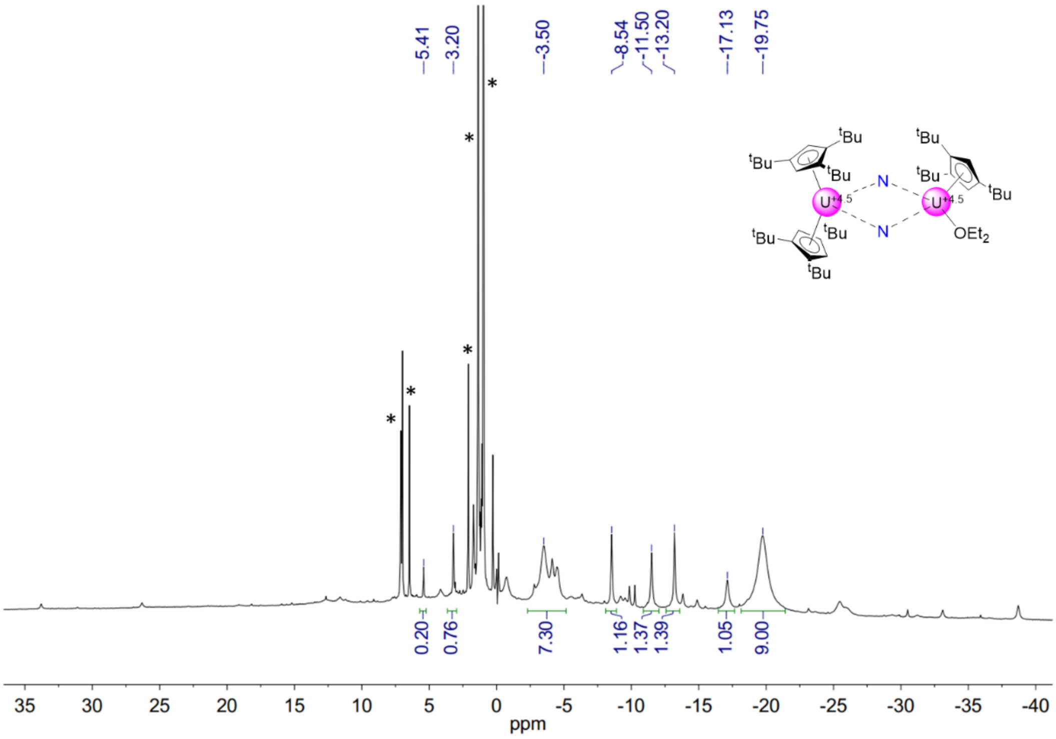


**Figure S32.** Expansion of $^1$H NMR spectrum of **4** in toluene-$d_8$ at 25 °C. An asterisk (*) marks residual toluene and traces of grease and Cp$^{ttt}_2$.[5]

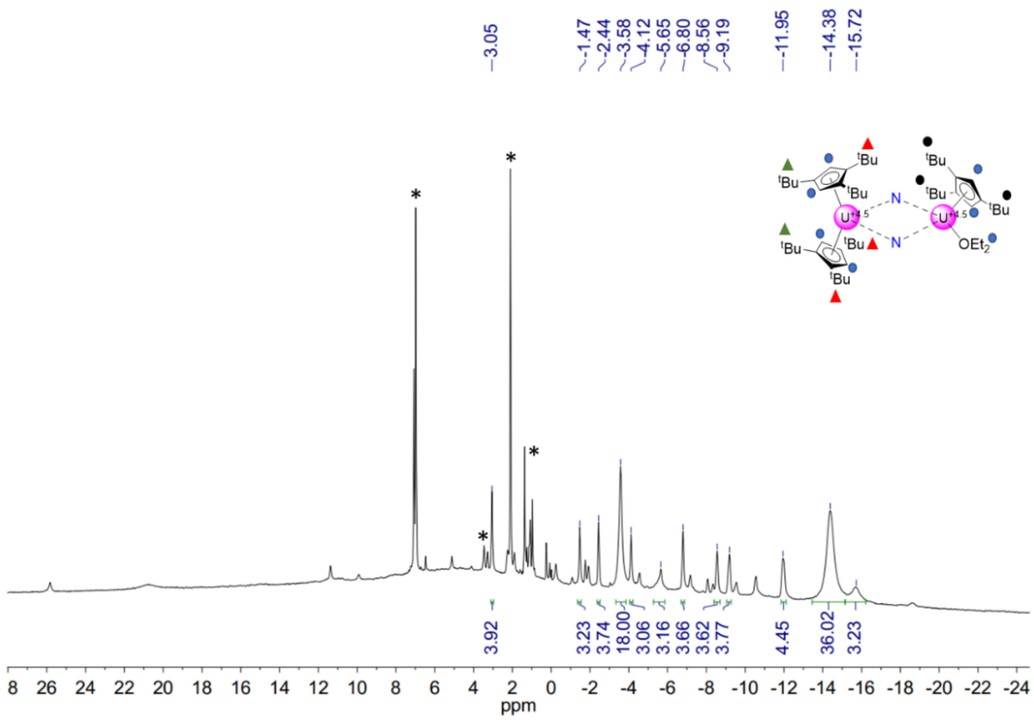

**Figure S33.** $^{1}$H NMR spectrum of **4** in toluene-$d_8$ at 80 °C. An asterisk (*) marks residual toluene and traces of grease and $Cp^{ttt}_2$.[5]

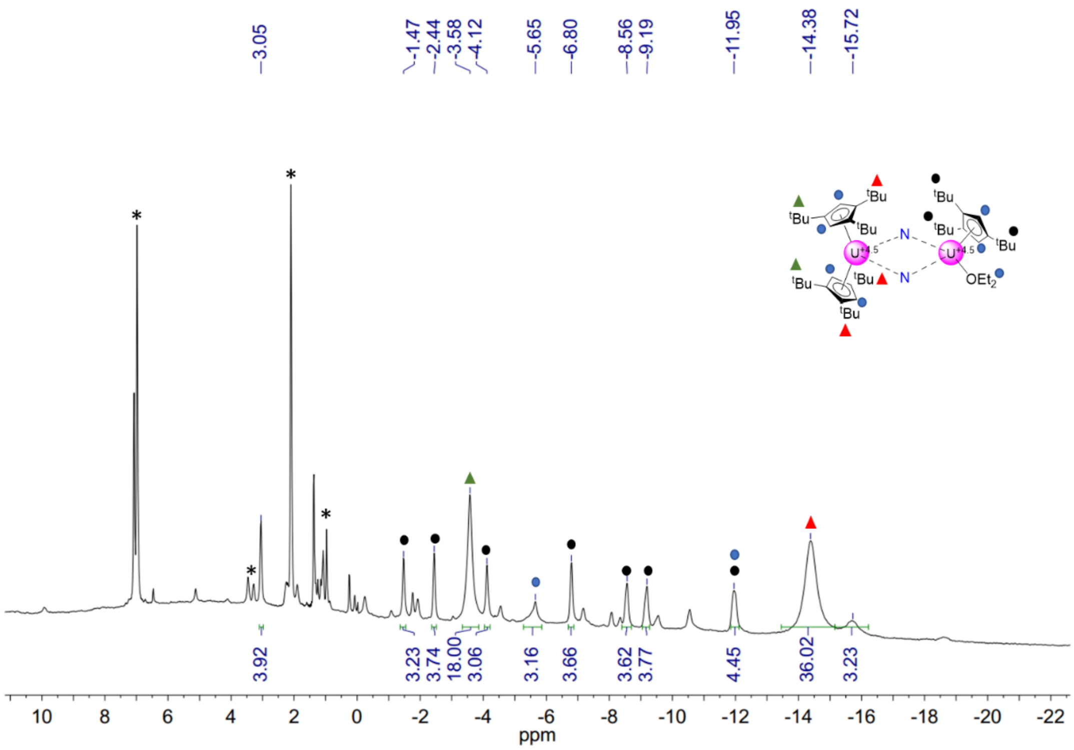

**Figure S34.** Expansion of $^{1}$H NMR spectrum of **4** in toluene-$d_8$ at 80 °C and tentative assignment of the resonances. An asterisk (*) marks residual toluene and traces of grease and $Cp^{ttt}_2$.[5]

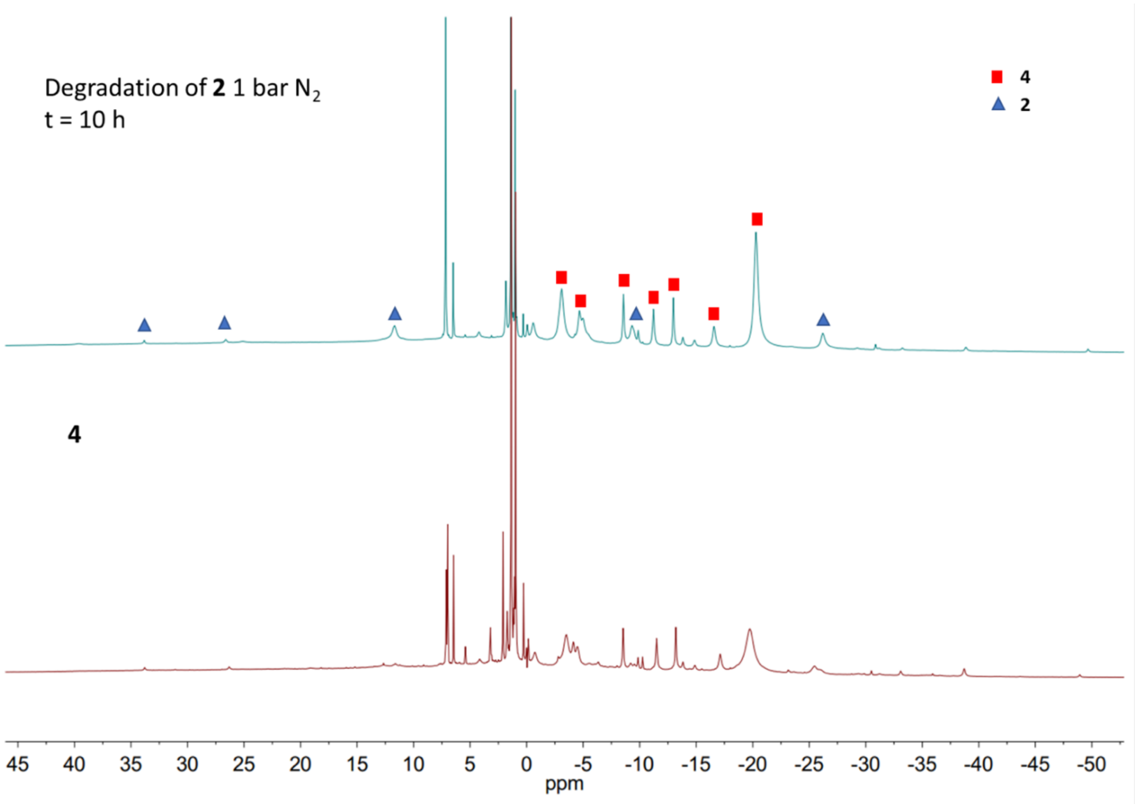


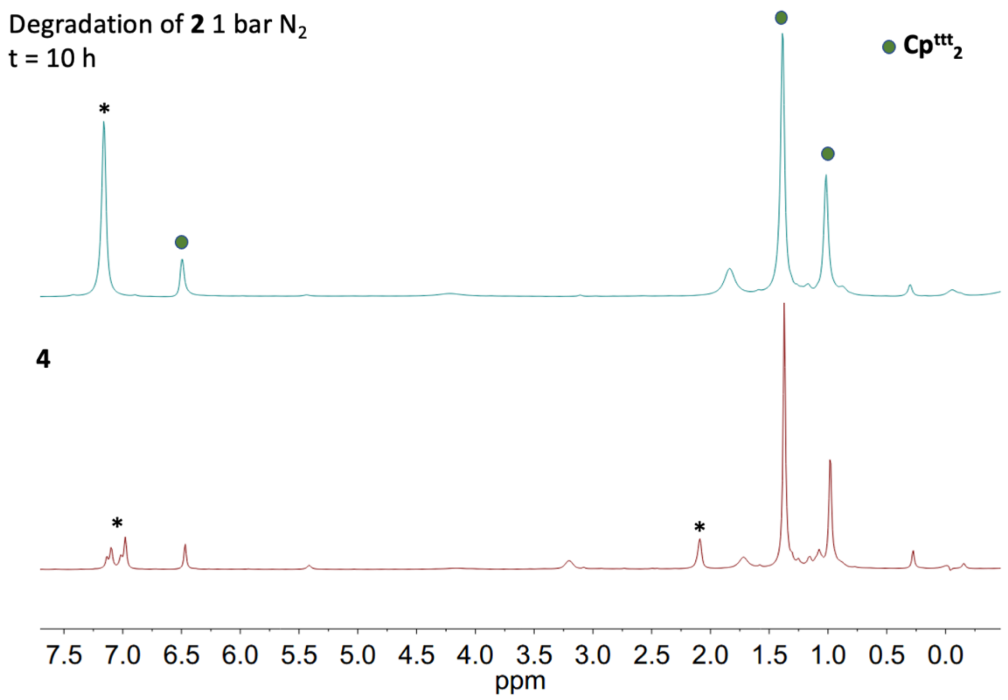


**Figure S35.** Stacked $^{1}$H NMR spectra of **2** after 10 hours with 1 bar of $N_2$ in benzene-$d_6$ (above) and spectrum of **4** in toluene-$d_8$ at 25 °C (below). Red squares mark the signals of **4**, while the blue triangles mark the remains of **2** (above). Zoom on the 0-8 ppm range with marks on the $Cp^{ttt}_2$ product.

## 2.k. NMR spectra for the hydrogenation of [$Cp^{ttt}{}_2U(\mu\text{-}N)_2\{U(Cp^{ttt})(OEt_2)\}$] (4)

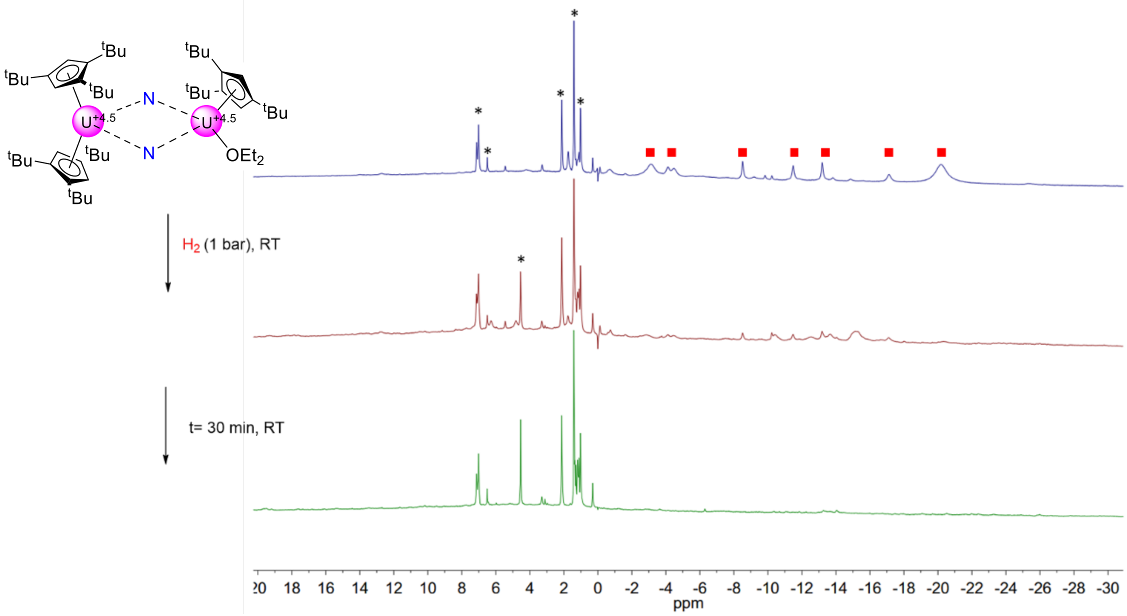


**Figure S36.** Stacked $^1$H NMR spectrum of **4** in toluene-$d_8$ (top), spectrum of **4** after the addition of 1 bar of $H_2$ in toluene-$d_8$ at 25 °C (middle) and **4** after the addition of 1 bar of $H_2$ in toluene-$d_8$ at 25 °C after 30 minutes (bottom).

## 2.l. NMR spectra of [Cp$^{ttt}$$_2$U(μ-I)(μ$_3$-N)(μ-NH)(UCp$^{ttt}$)$_2$] (5)

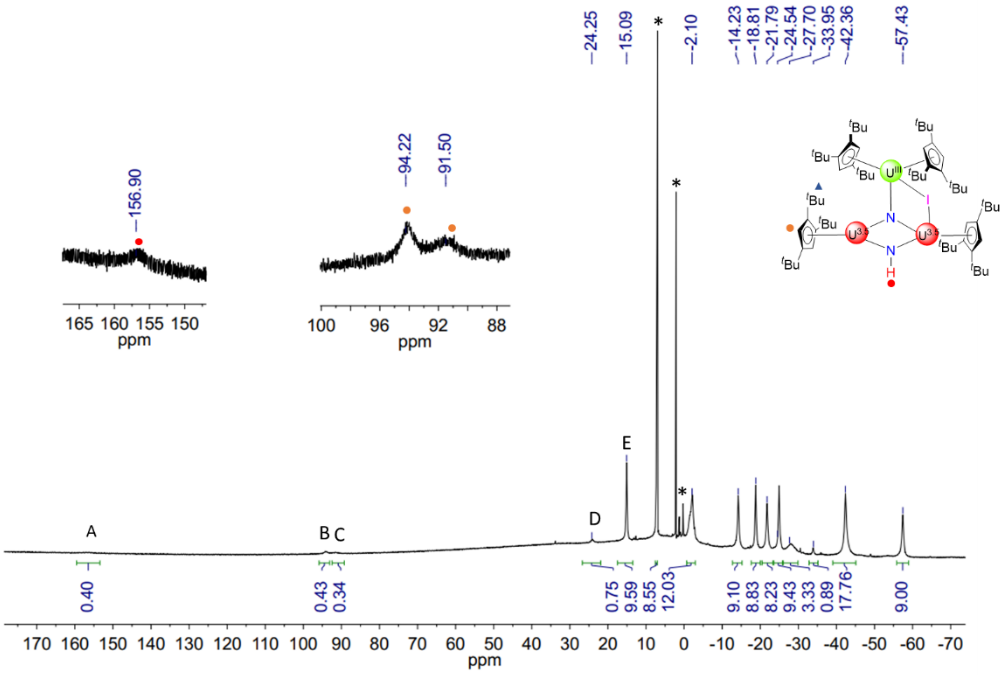

**Figure S37.** $^1$H NMR spectrum of **5** in toluene-$d_8$ at 25 °C. An asterisk (*) marks residual toluene and traces of grease and HCp$^{ttt}$. Lettering represents signal assignments for chemical shift versus 1/T plots (Figures S41-S43).

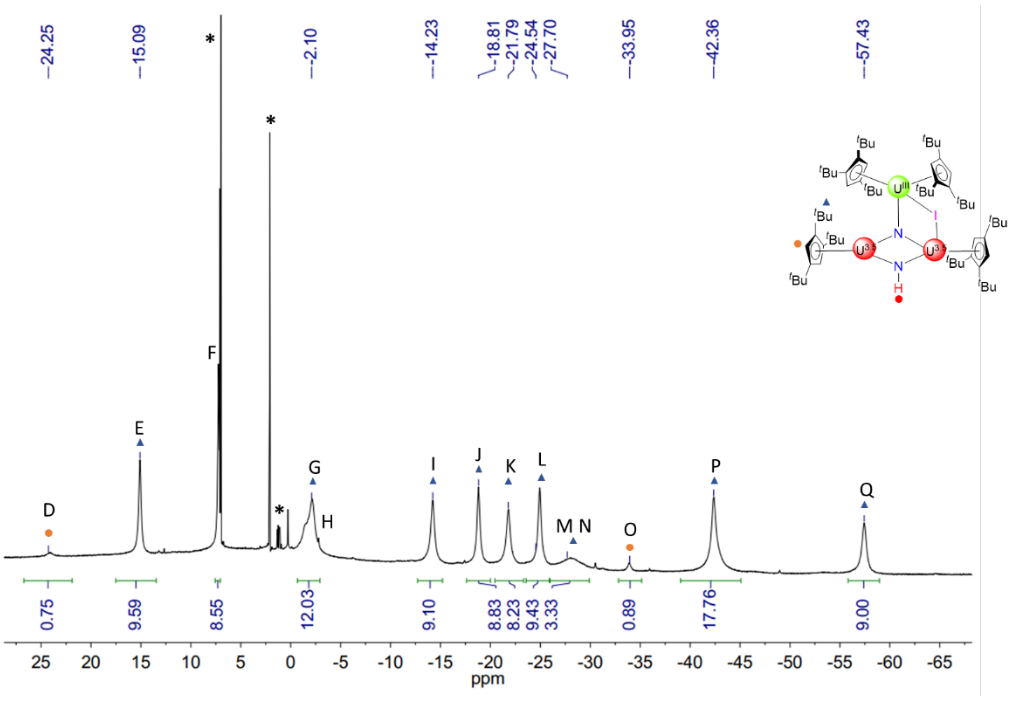

**Figure S38.** Expansion of $^1$H NMR spectrum of **5** in toluene-$d_8$ at 25 °C. An asterisk (*) marks residual toluene and traces of grease and HCp$^{ttt}$. Lettering represents signal assignments for chemical shift versus 1/T plots (Figures S41-S43).

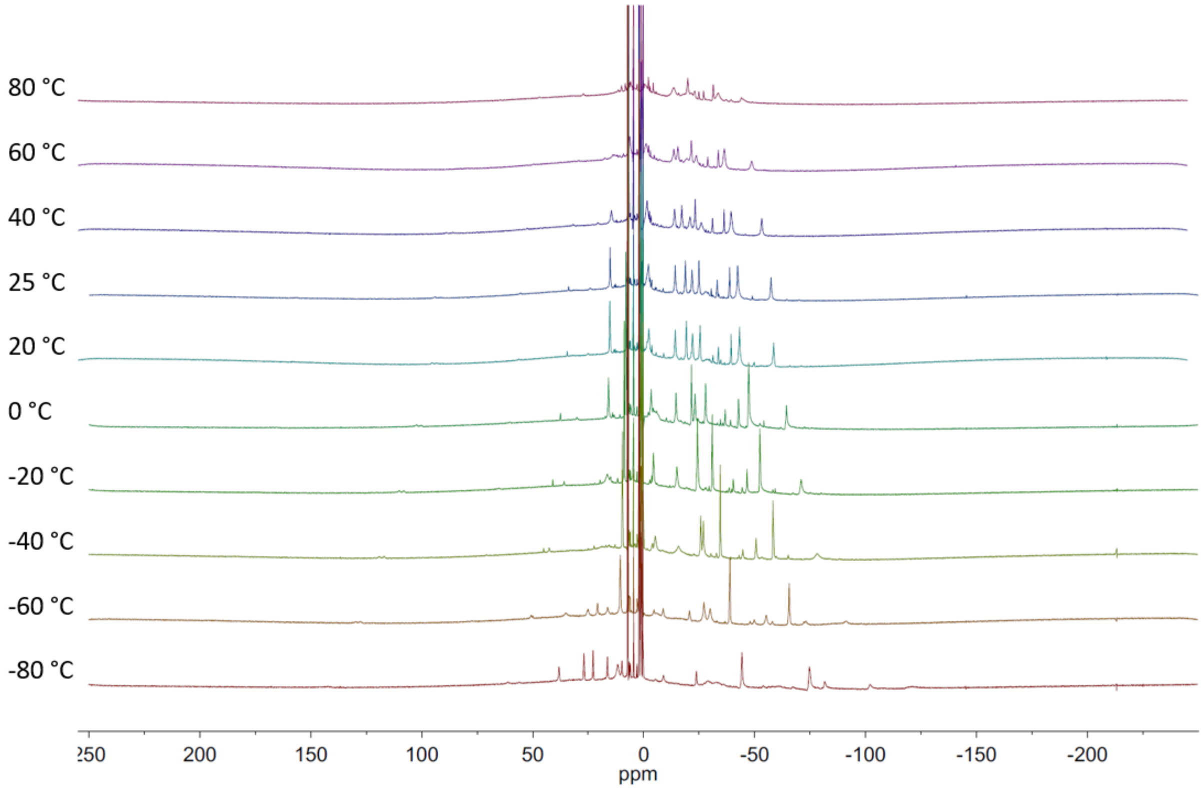


**Figure S39.** Variable temperature $^{1}H$ NMR spectra of **5** in toluene-$d_8$

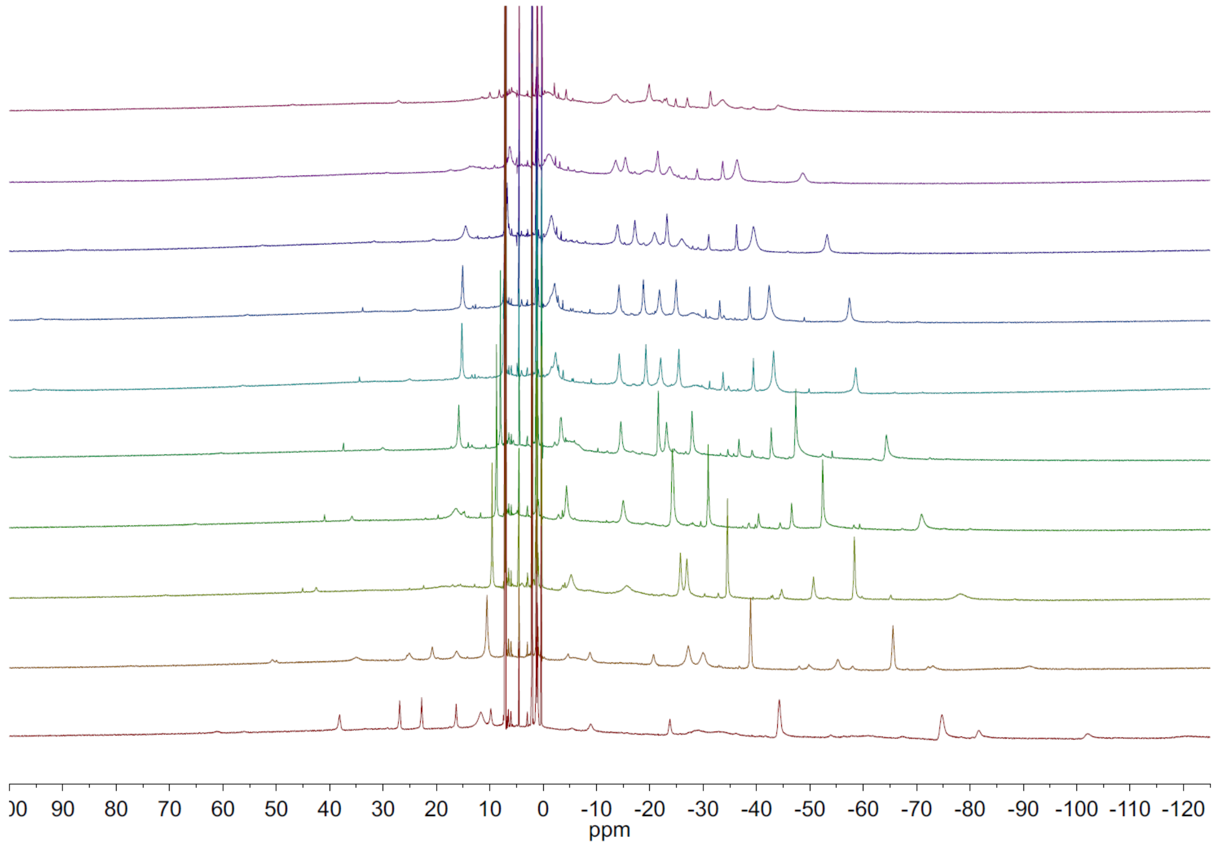


**Figure S40.** Expansion of variable temperature $^{1}H$ NMR spectra of **5** in toluene-$d_8$

## 2.m. Temperature dependency of chemical shift signals for NMR signals of 5

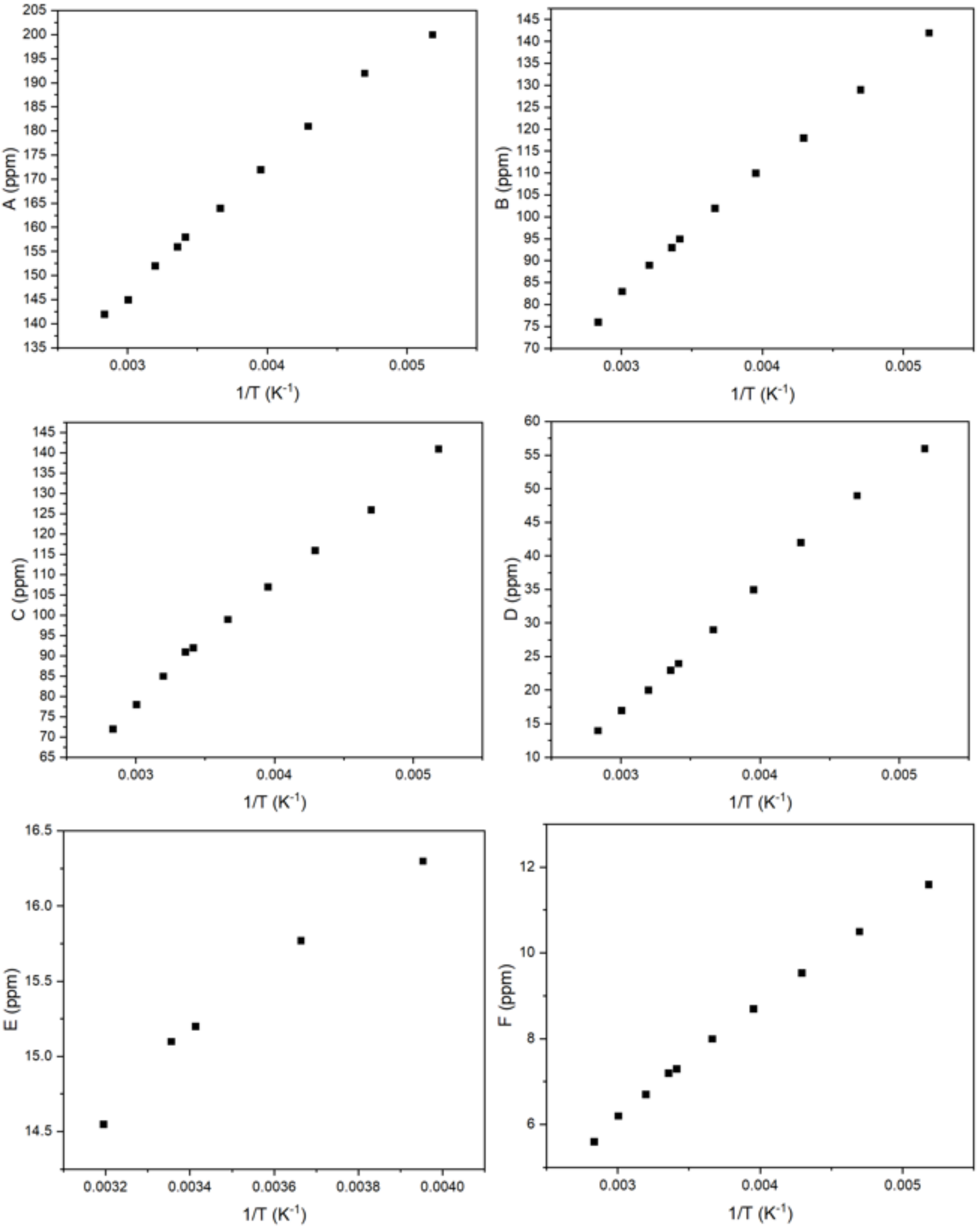


**Figure S41.** Chemical shift (δ) vs 1/T plots of **5**, signals A-F.

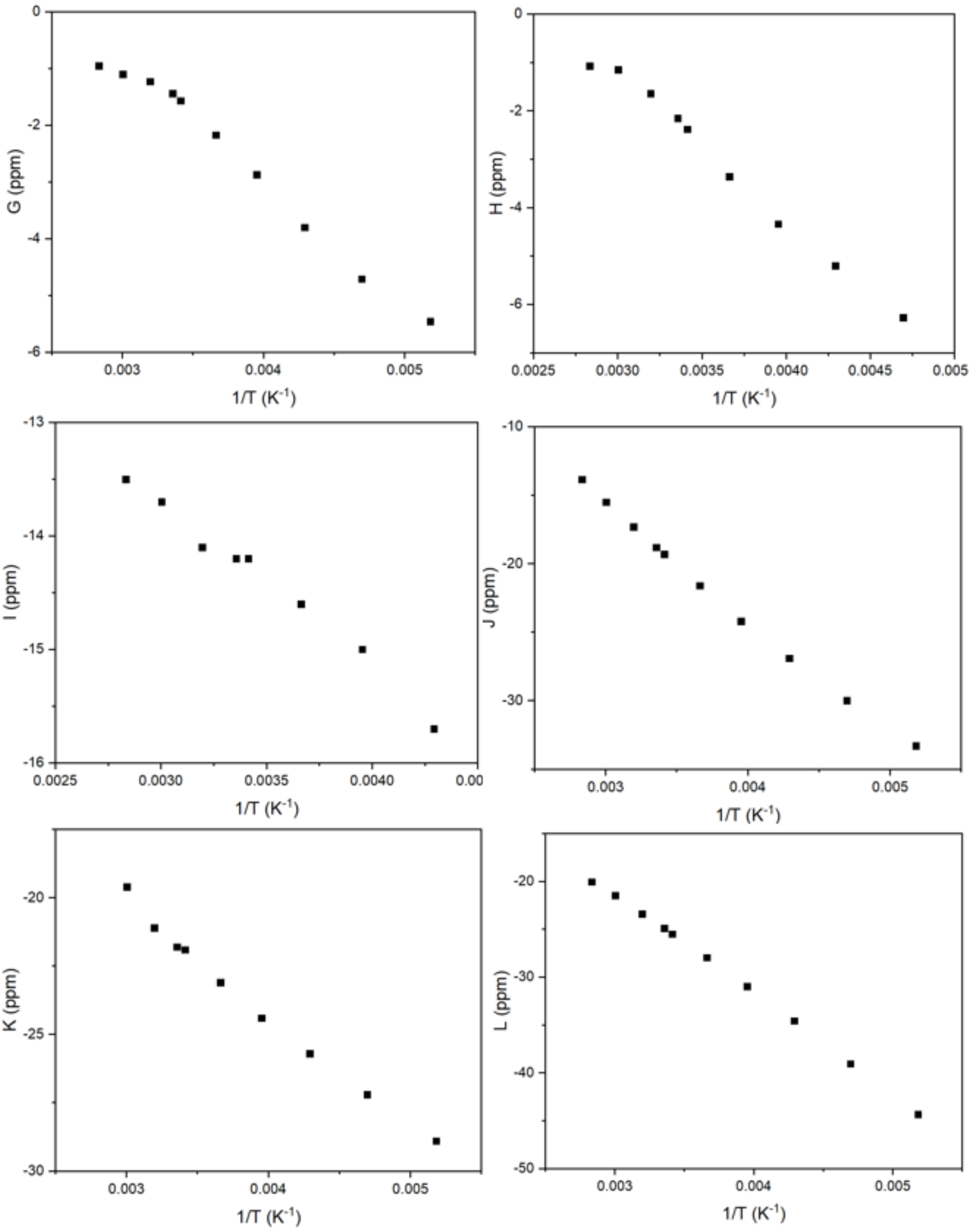


**Figure S42.** Chemical shift (δ) vs 1/T plots of **5**, signals G-L.

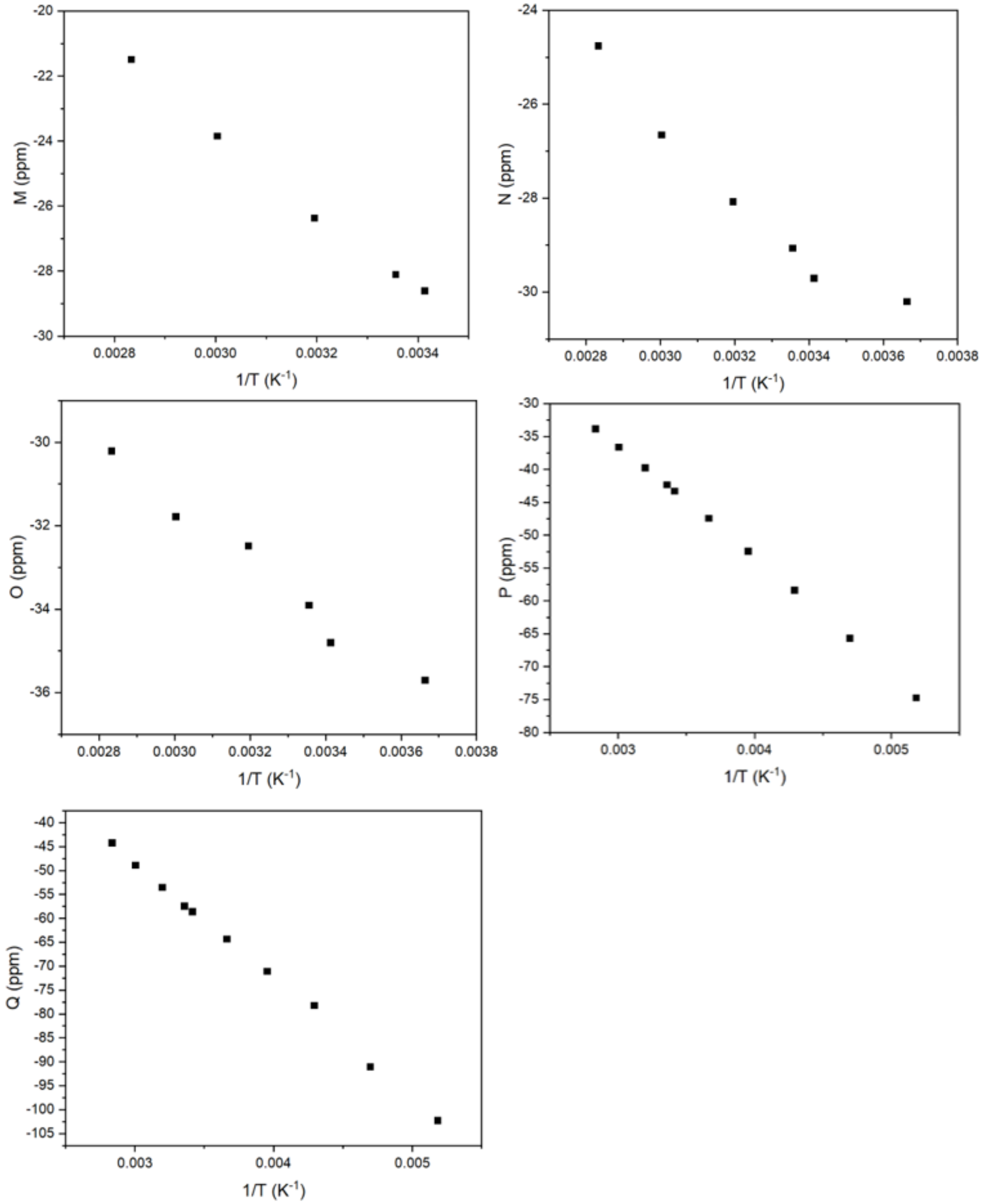


**Figure S43.** Chemical shift (δ) vs 1/T plots of **5**, signals M-Q.

### 2.n. Dehydrogenation and deuteration of $[Cp^{ttt}_2U(\mu\text{-}I)(\mu_3\text{-}N)(\mu\text{-}NH)(UCp^{ttt})_2]$ (5)

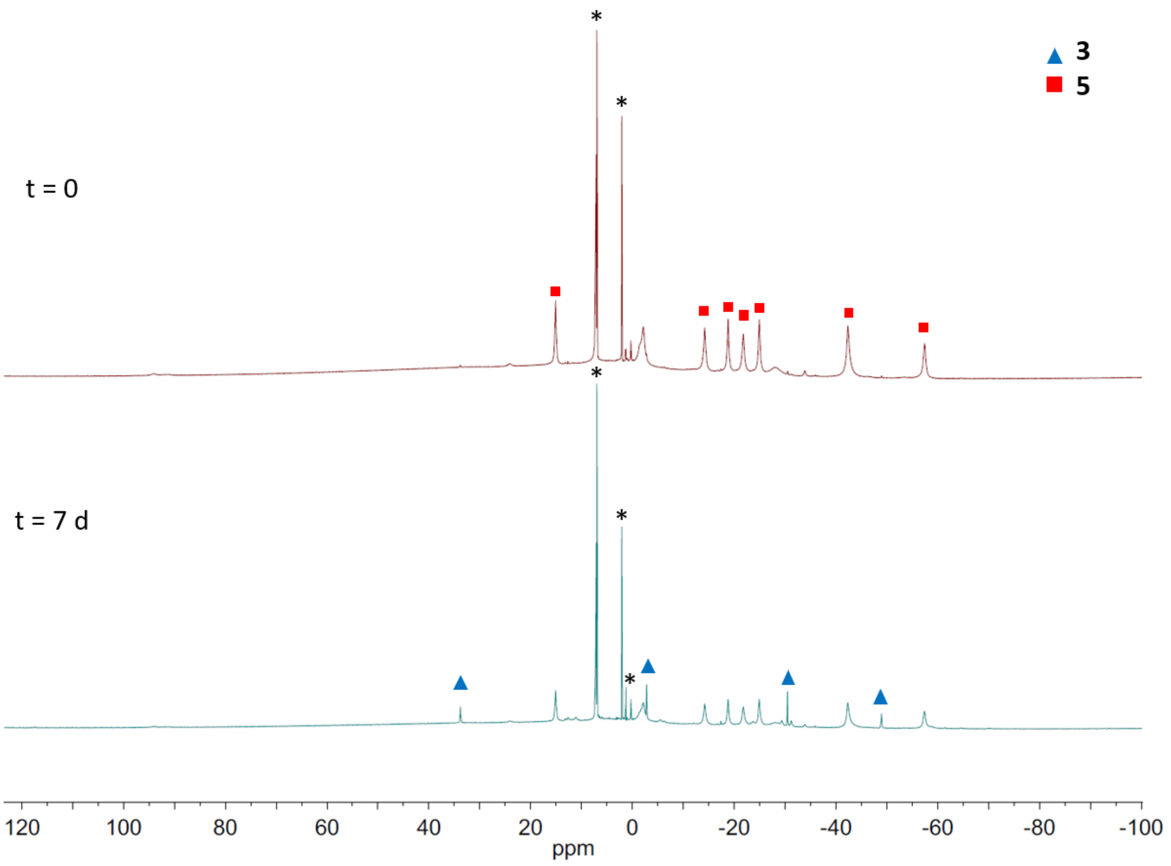


**Figure S44.** Stacked $^1$H NMR spectra of **5** in toluene-$d_8$ at 25 °C over time, showing partial conversion of **5** back into **3** after one week of standing in solution. An asterisk (*) marks residual toluene, traces of grease, and evolution of $HCp^{ttt}$. Red squares mark the signals of **5**, while the blue triangles indicate the partial regeneration of compound **3**.

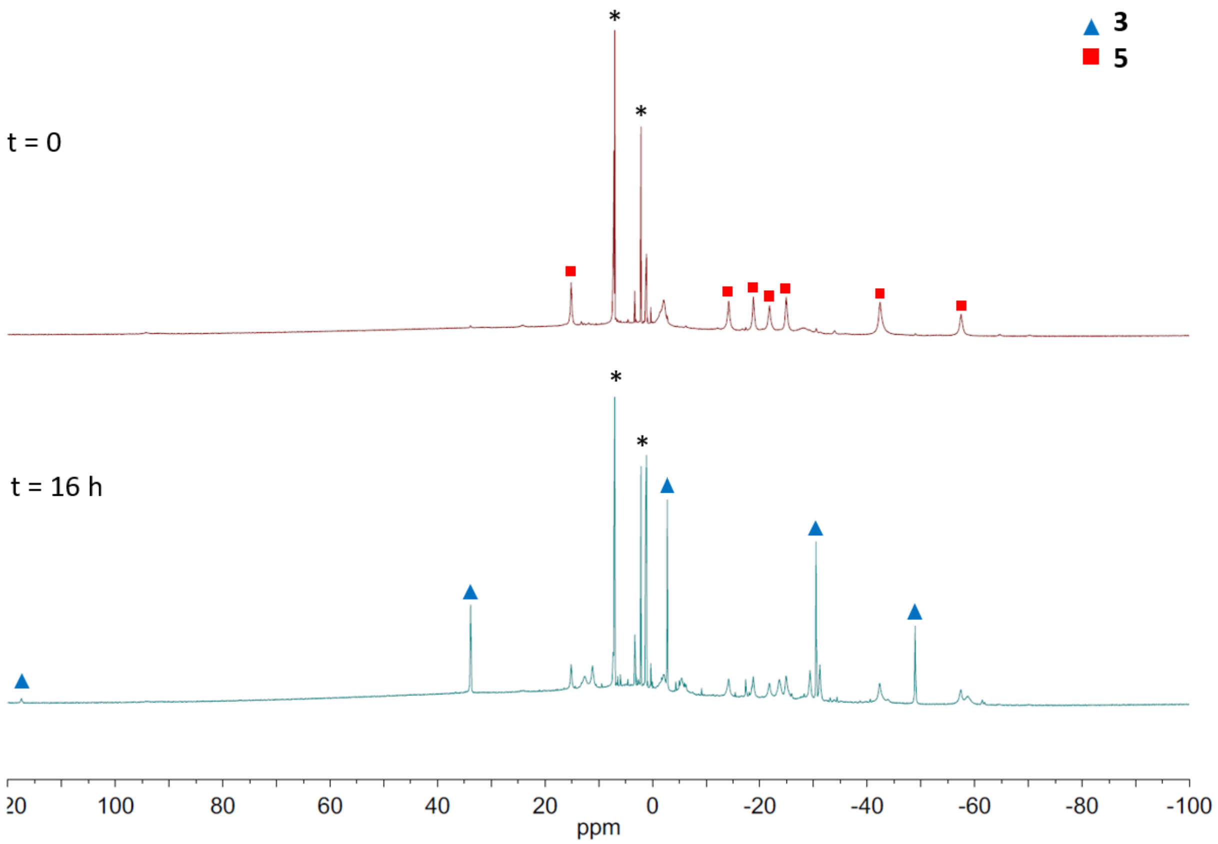


**Figure S45.** Stacked [1]H NMR spectra of **5** in toluene-$d_8$ at 25 °C over time with heating at 60 °C, showing significantly faster conversion of **5** back into **3** after 16 hours. An asterisk (*) marks residual toluene, traces of grease, and evolution of HCp$^{ttt}$. Red squares mark the signals of compound **5**, while the blue triangles indicate the partial regeneration of compound **3**.

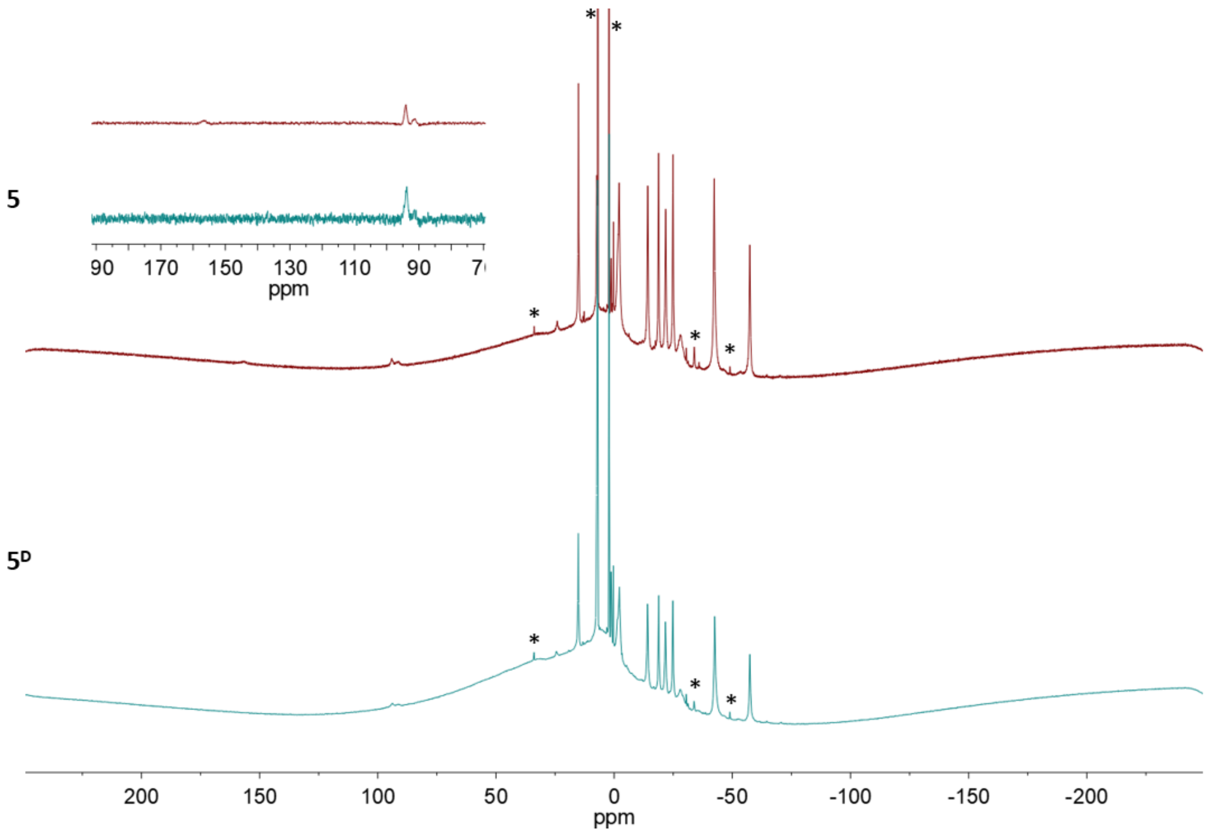


**Figure S46** Stacked [1]H NMR spectra of **5** (above) and the deuterated analogue, $\mathbf{5^D}$ (below). The expansion shows the absence of the signal at 156.4 ppm for $\mathbf{5^D}$, signifying that this represents an NH proton. An asterisk (*) marks residual toluene and traces of **3**.

## 2.o. Protonolysis of $[Cp^{ttt}{}_2U(\mu\text{-}I)(\mu_3\text{-}N)(\mu\text{-}NH)(UCp^{ttt})_2]$ (5)

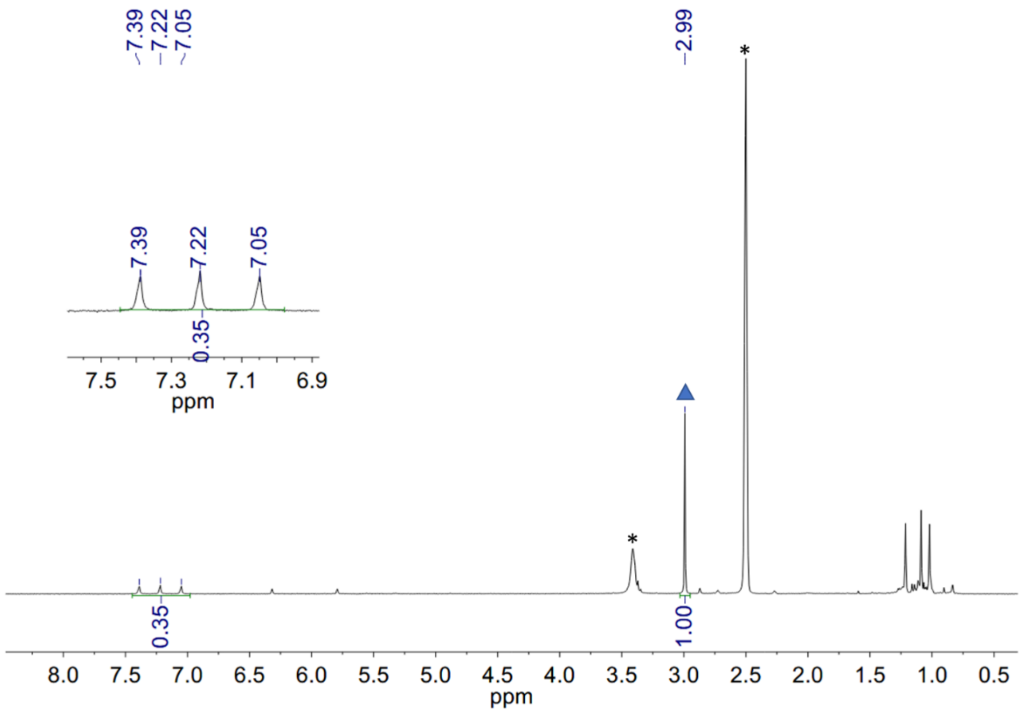


**Figure S47.** $^1$H NMR of **5** (2.15 µmol) after addition of excess $HCl\cdot Et_2O$ (2M) (100 µL, 200 µmol) in DMSO-$d_6$ (500 µL) at 25 °C showing the formation of $NH_4Cl$. Blue triangle marks the signals of $Me_2SO_2$ (7 mM) used as internal standard for the quantification of $NH_4Cl$ (1.91 µmol, yield: 44%). An asterisk (*) marks the residual DMSO-$d_6$.

## 3. Infrared spectra

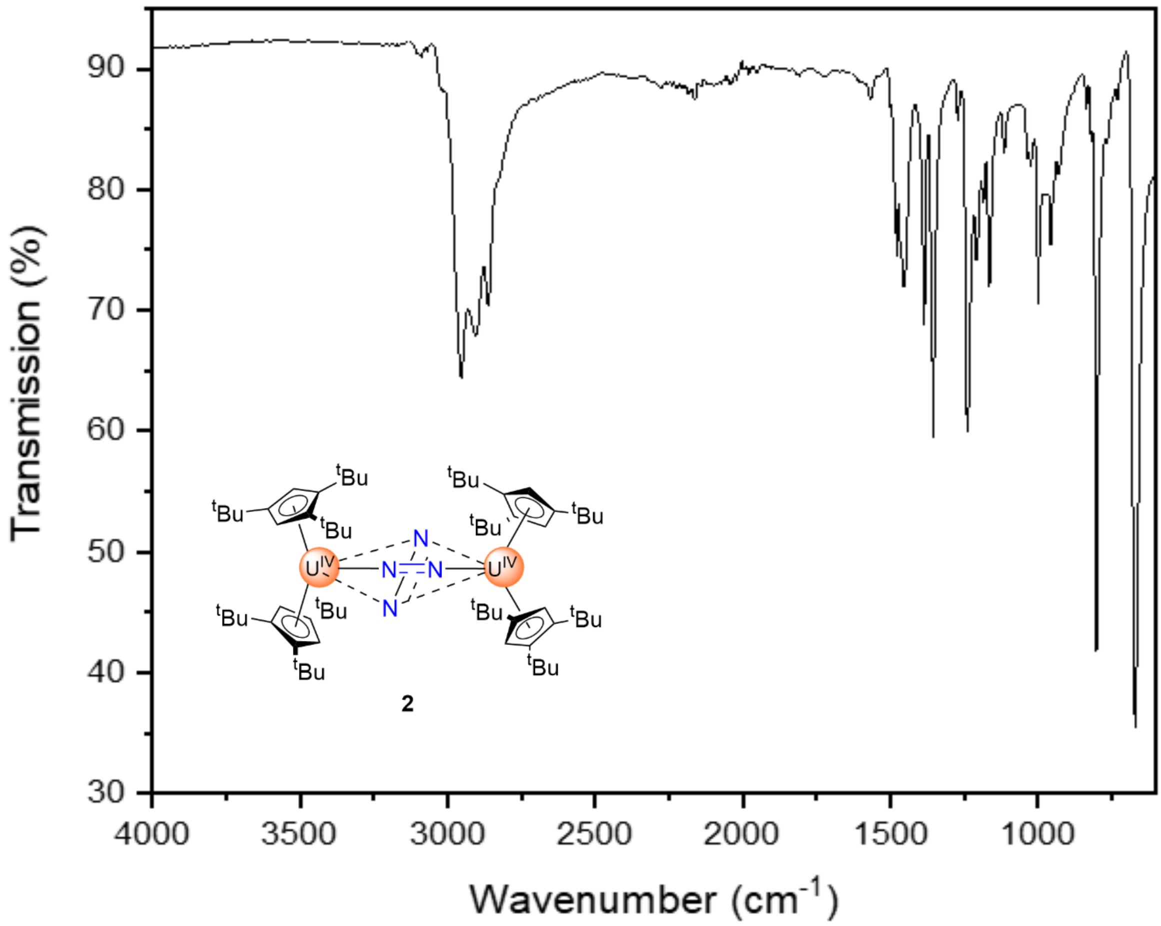


**Figure S48.** Infrared spectrum of $[(Cp^{ttt}{}_2U)_2(\mu\text{-}N_2)]$ (**2**)

IR (ATR, $cm^{-1}$): 2954 m, 2905 m, 2863 m, 1478 w, 1454 m, 1385 s, 1355 s, 1239 s, 1209 m, 1184 m, 1165 m, 1114 m, 1025 w, 999 s, 956 s, 892 s, 670 s.

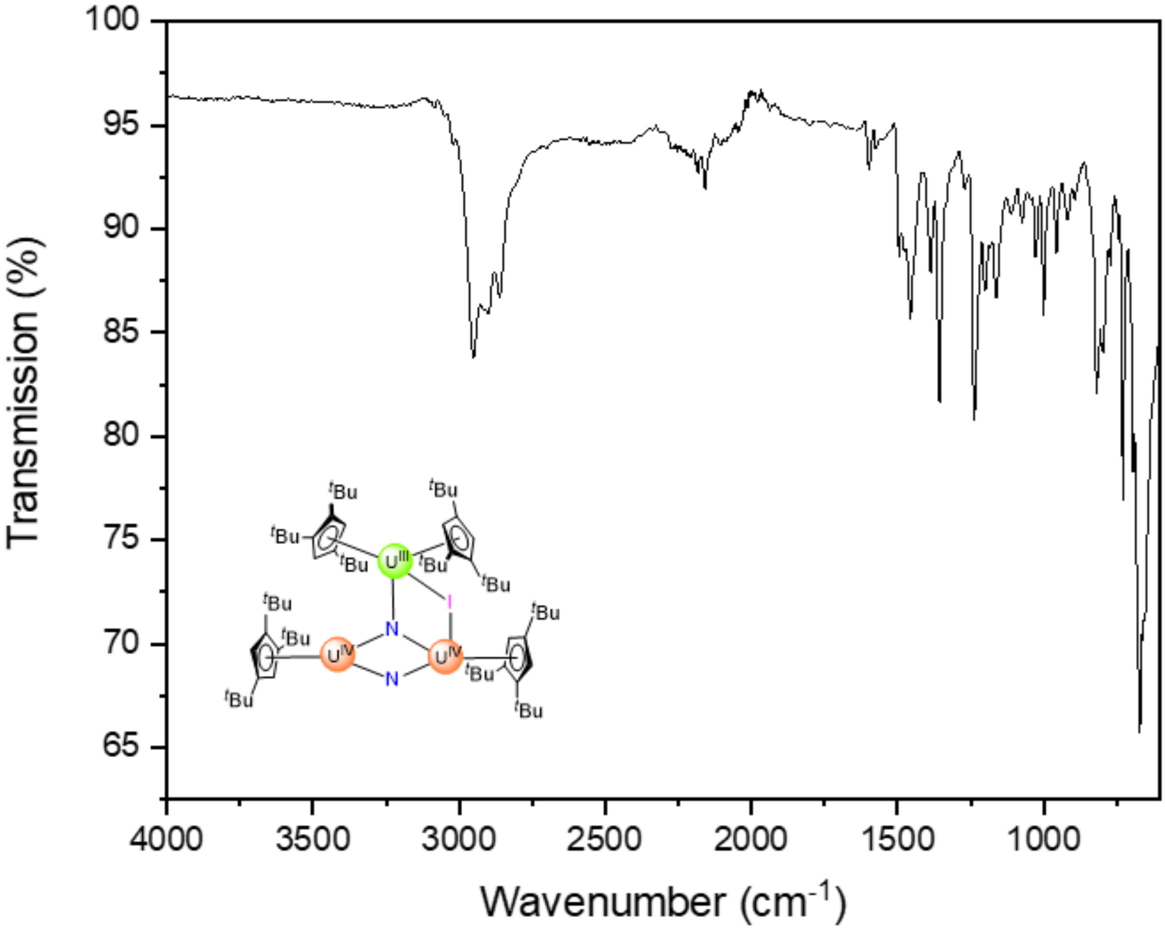


**Figure S49.** Infrared spectrum of [Cp$^{ttt}{}_2$U(μ-I)(μ$_3$-N)(μ-N)(UCp$^{ttt}$)$_2$] (**3**)

IR (ATR, cm$^{-1}$): 2951 m, 2900 m, 2861 m, 2157 m, 1596 m, 1494 w, 1455 m, 1385 m, 1356 s, 1269 w, 1356 s, 1269 w, 1237 s, 1199 m, 1162 m, 1111 w, 1072 w, 1027 w, 999 m, 955 m, 918 w, 893 w, 818 m, 796 w, 727 s, 693 s, 671 s.

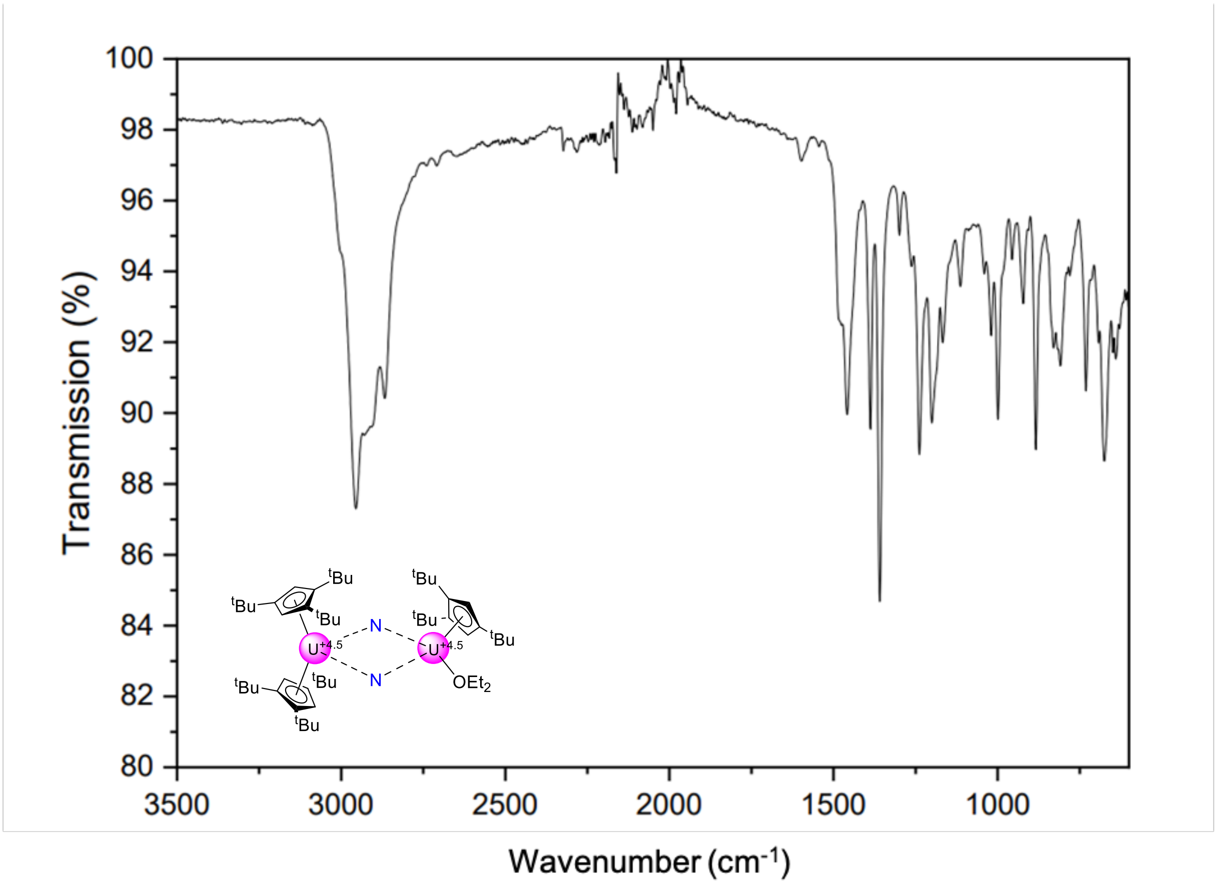


**Figure S50.** Infrared spectrum of [Cp$^{ttt}_2$U(μ-N)$_2${U(Cp$^{ttt}$)(OEt$_2$)}] (**4**)

IR (ATR, cm$^{-1}$): 2953 m, 2870 w, 1459 s, 1388 s, 1359 s, 1237 s, 1197 s, 1164 s, 1111 s, 998 s, 920 s, 959 s, 882 s, 805 s, 732 s, 672 s.

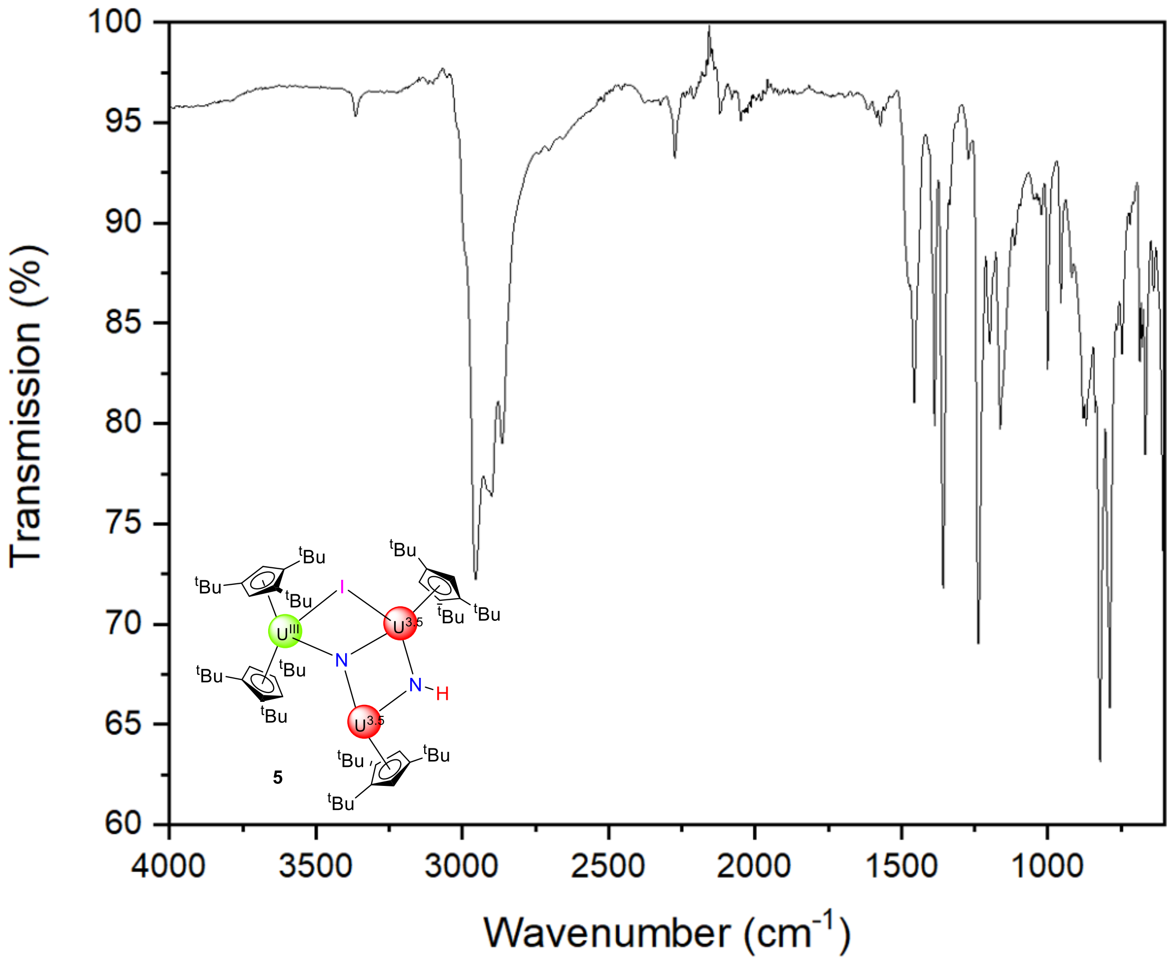


**Figure S51.** Infrared spectrum of [Cp$^{ttt}$$_2$U(μ-I)(μ$_3$-N)(μ-NH)(UCp$^{ttt}$)$_2$] (**5**)

IR (ATR, cm$^{-1}$): 3364 m, 2954 m, 2902 m, 2863 m, 2273 m, 2119 m, 2049 w, 1570 w, 1455 s, 1386 s, 1357 s, 1236 s, 1197 m, 1162 s, 1020 w, 999 s, 955 s, 868 m, 821 s, 787 s, 745 m, 685 m, 676 s, 666 m.

## 4. Raman spectra

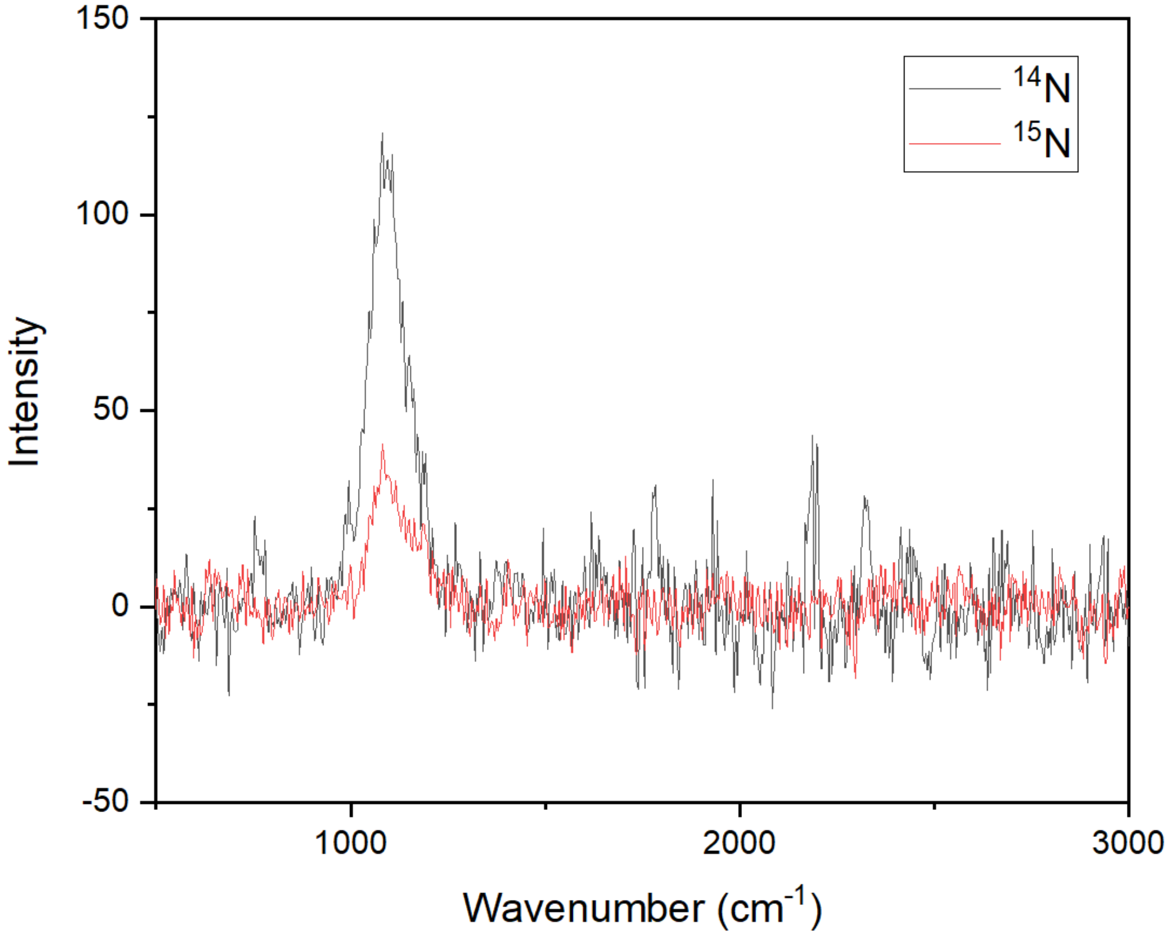


**Figure S52.** Stacked Raman spectra of [(Cp$^{ttt}$$_2$U)$_2$(μ-N$_2$)] (**2-$^{14}$N (**black) and **2-$^{15}$N** (red)) (powder in glass capillary, 473 nm laser, 50% laser power, 10 seconds acquisition, 3 accumulations).

# 5. UV-Visible Spectra

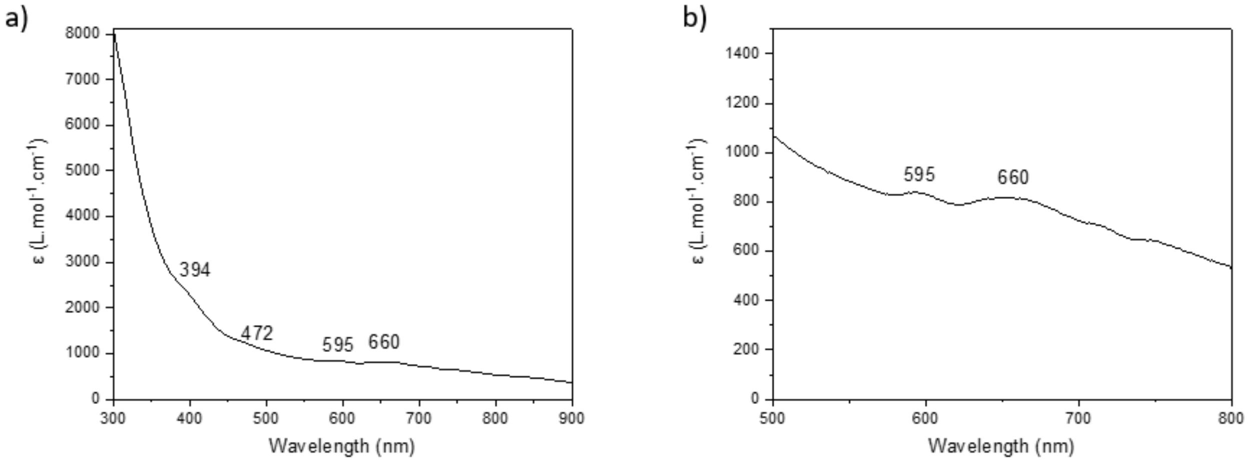


**Figure S53.** a) UV/Visible spectrum of **2** in toluene (7.27 x $10^{-4}$ M) and b) spectrum expansion from 300 nm to 800 nm.

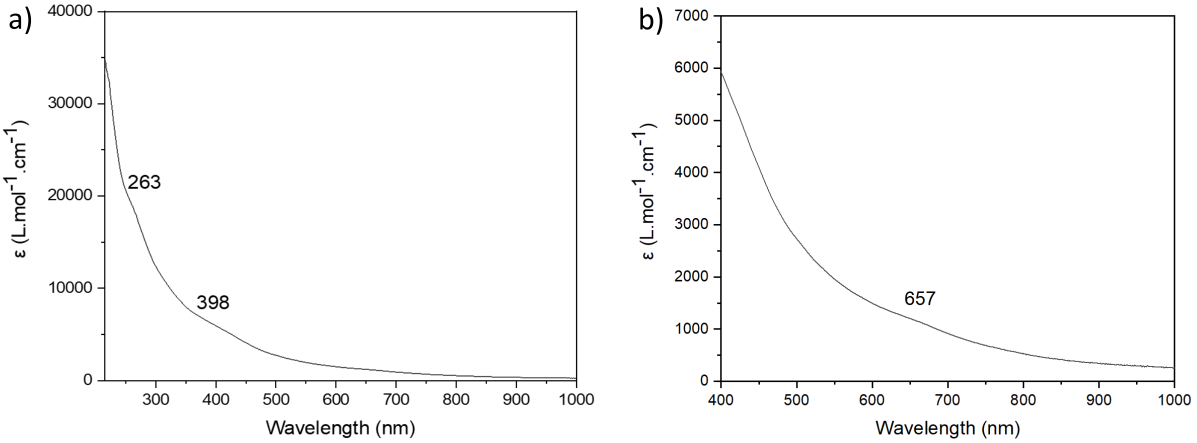


**Figure S54.** a) UV/Visible spectrum of **3** in pentane (4.99 x $10^{-5}$ M) and b) spectrum expansion from 400 nm to 1000 nm.

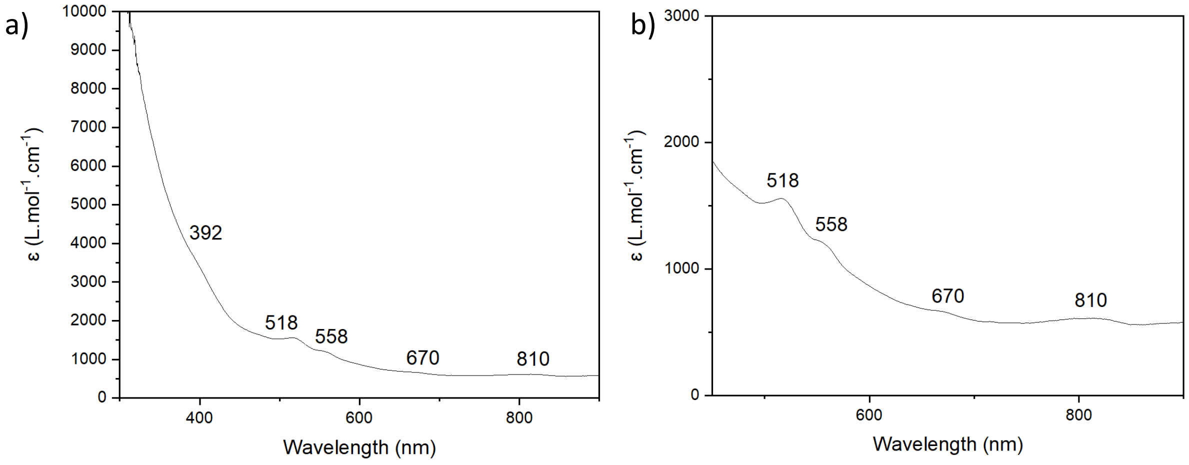


**Figure S55.** a) UV/Visible spectrum of **5** in pentane (3.54 x $10^{-4}$ M) and b) spectrum expansion from 400 nm to 900 nm.

# 6. Magnetic measurements

Magnetic samples of polycrystalline $[(Cp^{ttt}{}_2U)_2(\mu\text{-}N_2)]$ (**2**) and $[Cp^{ttt}{}_2U(\mu\text{-}I)(\mu_3\text{-}N)(\mu\text{-}N)(UCp^{ttt})_2]$ (**3**) were prepared in an argon-filled glovebox by adding with a diamagnetic spatula the finely grounded powder (10.7 mg and 11.7 mg respectively) of each compound to 3 mm quartz tubes. In each sample, the fine powder was pressed between two layers of quartz wool to prevent sample displacement during measurement. The tubes were fitted with rubber adapters and put under a partial static vacuum before flame-sealing them using a butane/$O_2$ torch. The samples were preserved at -40 °C before measurement. Magnetic measurements were performed using a SQUID magnetometer (MPMS Quantum Design).

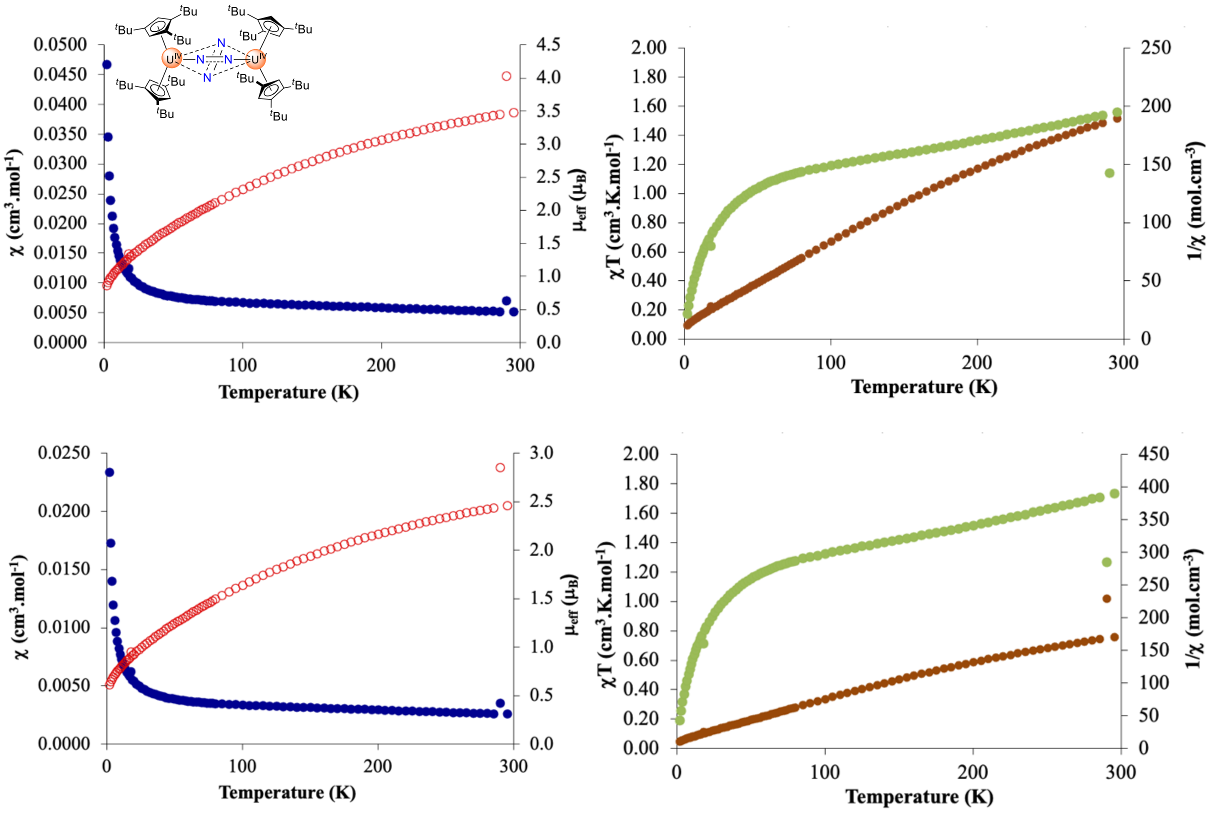


**Figure S56.** Plot of $\chi_M$, blue dots and $\mu_{eff}(\mu_B)$, unfilled red dots, versus temperature (K), left, and of $\chi_M T$, green dots and $1/\chi_M$, orange dots, versus temperature (K), right, for $[(Cp^{ttt}{}_2U)_2(\mu\text{-}N_2)]$ (**2**) under 0.5 d.c. field. Below, the same plots are given per uranium ion.

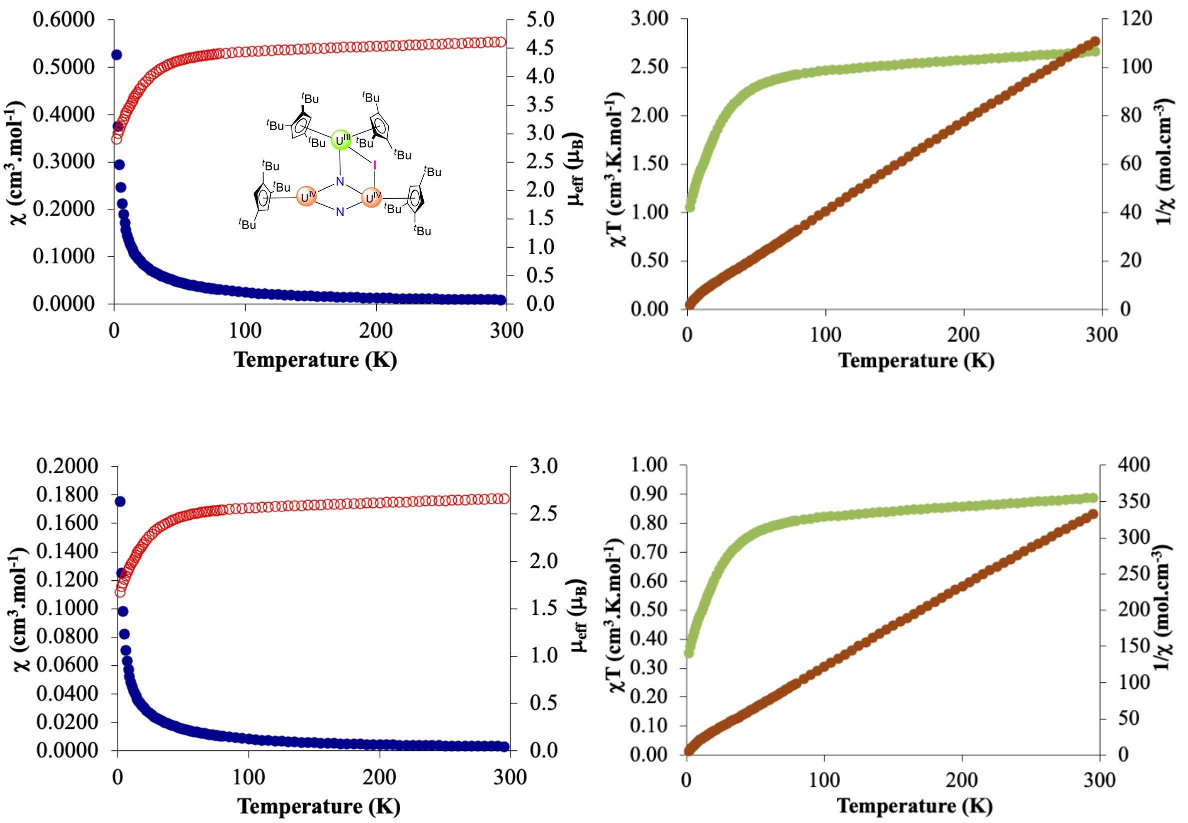


**Figure S57.** Plot of $\chi_M$, blue dots and $\mu_{eff}(\mu_B)$, unfilled red dots, versus temperature (K), left, and of $\chi_M T$, green dots and $1/\chi_M$, orange dots, versus temperature (K), right, for $[Cp^{ttt}{}_2U(\mu\text{-}I)(\mu_3\text{-}N)(\mu\text{-}N)(UCp^{ttt})_2]$ (**3**) under 0.5 d.c. field. Below, the same plots are given per uranium ion.

# 7. X-ray crystallography

## 7.a. General methods

Single crystals of the complexes suitable for X-ray analysis covered with viscous hydrocarbon oil were mounted on a Kapton loop. Data were obtained at -123 °C (150 K) unless otherwise stated using a STOE Stradivari instrument equipped with an EIGER2 1M CdTe detector and a Mo microfocus source (MoKα, λ=0.71073). The resolution of solid-state structures was accomplished using SHELXS-97 or SHELXT programs[6,7] where the refinement was performed with SHELXL[8] program using *Olex2* software.[9] All atoms except hydrogens were refined anisotropically, and hydrogen atoms were placed in calculated positions using the riding model. Data collection and refinement details are summarized below (Table S2 -S3). Crystallographic data for the structures reported in this paper have been deposited with the Cambridge Crystallographic Data Centre (CCDC # 2464092, 2464093, 2464096, 2464097, 2464099 for **2**, **3**, **4**, **5**, and **A**, respectively) and can be obtained free of charge from https://www.ccdc.cam.ac.uk/structures/ or from the Cambridge Crystallographic Data Centre, 12 Union Road, Cambridge CB2 1EZ, UK; tel: + 44 (0)1223 336408; or e-mail: deposit@ccdc.cam.ac.uk

## 7.b. Summary of crystal data

**Table S2.** Crystal data and structural refinement for complexes **2, 3** and **4**.

| Compound | **2** | **3** | **4** |
|---|---|---|---|
| Empirical Formula | $C_{73}H_{128}N_2U_2$ | $C_{89}H_{140}IN_2U_3$ | $C_{55}H_{97}N_2OU_2$ |
| Forpmula weigh | 1509.83 | 2079.01 | 1278.40 |
| Crystal system | Monoclinic | Triclinic | Monoclinic |
| Space Group | *P*2/*n* | *P*-1 | *P*2<sub></sub>$_1$/*n* |
| Unit cell dimensions | | | |
| a(Å) | 18.4067(9) | 14.0922(3) | 10.5775(12) |
| b(Å) | 10.3962(5) | 14.3701(3) | 25.372(4) |
| c(Å) | 19.3564(9) | 23.5776(5) | 21.043(3) |
| α° | 90 | 77.506(2) | 90 |
| β° | 109.753(3) | 82.208(2) | 101.426(9) |
| γ° | 90 | 65.737(2) | 90 |
| Unit cell volume(Å$^3$) | 3486.1(3) | 4243.94(17) | 5535.4(12) |
| Z | 2 | 2 | 4 |
| $\rho_{calc}$g/cm$^3$ | 1.438 | 1.627 | 1.534 |
| μ/mm$^{-1}$ | 4.679 | 6.116 | 5.880 |
| F(000) | 1528.0 | 2034.0 | 2532.0 |
| Crystal size mm$^3$ | 0.203 x 0.032 x 0.024 | 0.25 × 0.162 × 0.08 | 0.21 × 0.14 × 0.14 |
| 2Θ range (°) | 4.472 to 49.996 | 4.42 to 54.206 | 4.244 to 52.044 |
| Index ranges | $-21 \leq h \leq 21$, $-12 \leq k \leq 12$, $-23 \leq l \leq 22$ | $-18 \leq h \leq 17$, $-18 \leq k \leq 17$, $-30 \leq l \leq 30$ | $-13 \leq h \leq 13$, $-31 \leq k \leq 31$, $-25 \leq l \leq 25$ |
| Reflection collected | 17049 | 72299 | 41618 |
| Independent reflections | 6098 [$R_{int}$ = 0.0773, $R_{sigma}$ = 0.0925] | 18716 [$R_{int}$ = 0.0315, $R_{sigma}$ = 0.0327] | 10873 [$R_{int}$ = 0.0432, $R_{sigma}$ = 0.0338] |
| Data/restraints/ parameters | 6098/45/386 | 18716/384/943 | 10873/930/767 |
| Goodness-of-fit on F$^2$ | 1.015 | 1.009 | 1.060 |
| Final R$^2$ indexes [I≥2σ(I)] | $R_1$ = 0.0629, w$R_2$ = 0.1438 | $R_1$ = 0.0254, wR2 = 0.0567 | $R_1$ = 0.0536, w$R_2$ = 0.1376 |
| Final R$^2$ indexes [all data] | $R_1$ = 0.1211, w$R_2$ = 0.1742 | $R_1$ = 0.0375, wR2 = 0.0594 | $R_1$ = 0.0672, w$R_2$ = 0.1487 |
| Largest diff. Peak/hole/e.Å-$^3$ | 1.39/-1.62 | 3.06/-0.99 | 4.26/-2.05 |
| CCDC Number | 2464092 | 2464093 | 2464096 |

**Table S3.** Crystal data and structural refinement for complex **5** and **A**.

| Compound | **5** | **A** |
|---|---|---|
| Empirical Formula | $C_{89}H_{141}IN_2U_3$ | $C_{73}H_{130}N_2U_2$ |
| Forpmula weigh | 2080.02 | 1511.84 |
| Crystal system | Triclinic | Monoclinic |
| Space Group | *P*-1 | *C*2/*c* |
| Unit cell dimensions | | |
| a(Å) | 14.2226(4) | 44.3908(7) |
| b(Å) | 14.7263(3) | 11.6107(2) |
| c(Å) | 22.8579(6) | 56.6934(11) |
| α° | 78.484(2) | 90 |
| β° | 83.187(2) | 101.829(1) |
| γ° | 65.206(2) | 90 |
| Unit cell volume(Å$^3$) | 4255.4(2) | 28599.7(9) |
| Z | 2 | 16 |
| $\rho_{calc}$g/cm$^3$ | 1.623 | 1.404 |
| μ/mm$^{-1}$ | 6.100 | 4.563 |
| F(000) | 2036.0 | 12256.0 |
| Crystal size mm$^3$ | 0.187 × 0.163 × 0.139 | 0.16 × 0.077 × 0.033 |
| 2Θ range (°) | 4.366 to 54.968 | 4.462 to 50.054 |
| Index ranges | $-18 \leq h \leq 17$, $-19 \leq k \leq 18$, $-29 \leq l \leq 29$ | $-28 \leq h \leq 52$, $-13 \leq k \leq 13$, $-67 \leq l \leq 67$ |
| Reflection collected | 69853 | 81690 |
| Independent reflections | 19358 [$R_{int}$ = 0.0425, $R_{sigma}$ = 0.0593] | 25119 [$R_{int}$ = 0.0475, $R_{sigma}$ = 0.0506] |
| Data/restraints/ parameters | 19358/382/939 | 25119/76/1463 |
| Goodness-of-fit on F$^2$ | 0.981 | 1.047 |
| Final R$^2$ indexes [I≥2σ(I)] | $R_1$ = 0.0324, $wR_2$ = 0.0667 | $R_1$ = 0.0447, $wR_2$ = 0.0924 |
| Final R$^2$ indexes [all data] | $R_1$ = 0.0567, $wR_2$ = 0.0716 | $R_1$ = 0.0774, $wR_2$ = 0.1041 |
| Largest diff. Peak/hole/e.Å-$^3$ | 1.13/-0.83 | 2.15/-0.98 |
| CCDC Number | 2464097 | 2464099 |

### 7.c. Crystal structure of $[(Cp^{ttt}{}_2U)_2(\mu\text{-}N_2)]$ (2)

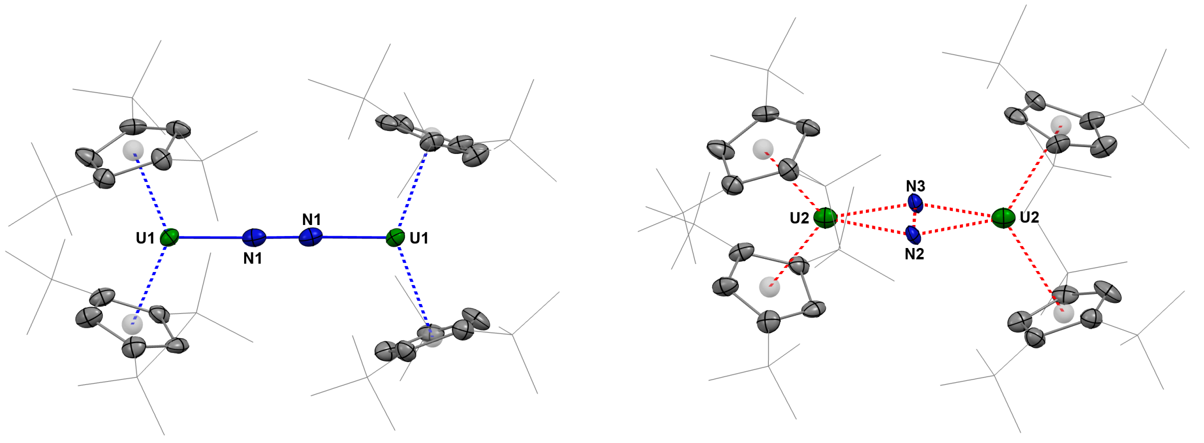


**Figure S58.** ORTEP diagrams of **2-end-on** (left) and **2-side-on** (right) showing atom numbering scheme for relevant atoms. Hydrogen atoms are omitted for clarity. Thermal ellipsoids are drawn at the 50% probability level.

### 7.d. Analysis of the bond distance in $Ln\text{-}N_2\text{-}Ln$ complexes

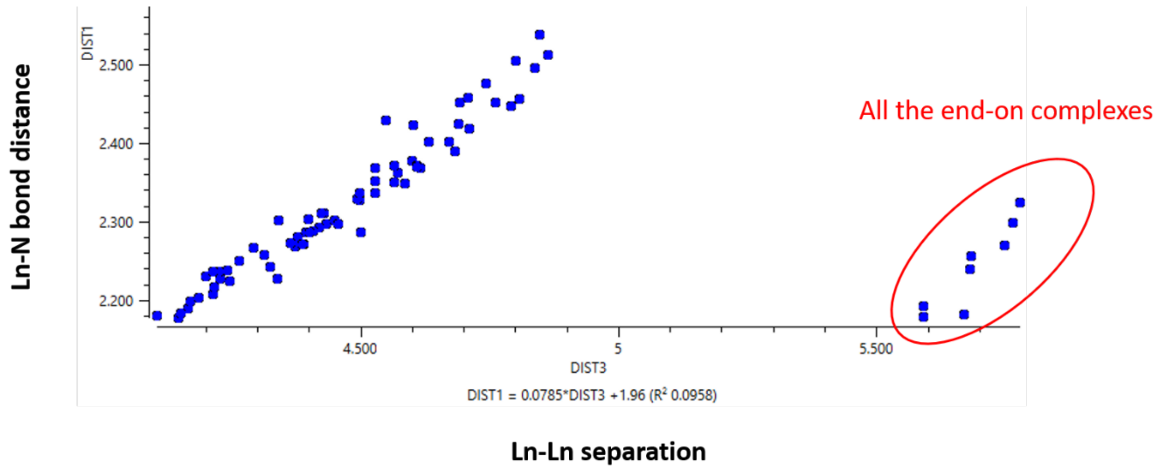


**Figure S59.** Analysis of the M-M distance in $Ln\text{-}N_2\text{-}Ln$ complexes related to the Ln-N bond distance. The investigation was carried out in 12/2024 on the CCDC database using Conquest and Mercury.[10] Values are provided in Å ($1 \cdot 10^{-10}$ m).

**Table S4.** Main metric parameters for **2-5**. Distances are given in Å and angles in °.

| | **2-end-on** | **2-side-on** | **3** | **4** | **5** |
|---|---|---|---|---|---|
| U-Cp(Ctr) | 2.515(4)<br>2.506(5) | 2.754(7)<br>2.778(7) | U1, 2.5086(16)<br>U2, 2.546(2)<br>U3, 2.6397(16)<br>U3, 2.6252(15) | U1, 2.613(4)<br>U1, 2.586(4)<br>U2, 2.520(14)<br>U2, 2.606(13) | U1, 2.513(2)<br>U2, 2.533(3)<br>U3, 2.620(2)<br>U3, 2.632(2) |
| U-N | 1.958(12) | 2.194(16)<br>2.211(16) | U1-N1, 2.027(3)<br>U1-N2, 2.221(3)<br>U2-N1, 2.033(3)<br>U2-N2, 2.170(3)<br>U3-N2, 2.284(3) | U1-N1, 2.031(7)<br>U1-N2, 2.061(7)<br>U2-N1, 2.094(7)<br>U2-N2, 2.047(7) | U1-N1, 2.173(4)<br>U1-N2, 2.186(4)<br>U2-N1, 2.158(4)<br>U2-N2, 2.141(4)<br>U3-N2, 2.315(3) |
| U-L | - | - | U1-I1, 3.1065(3)<br>U3-I1, 3.2293(3) | O1, 2.573(7) | U1-I1, 3.1397(3)<br>U3-I1, 3.1943(4) |
| Cp(Ctr)-U-Cp(Ctr) | 139.2 | 116.6 | 134.3 | 128.3 | 134.3 |

**Table S5.** Main metric parameters for compound **A**. Distances are given in Å and angles in °.

| | **A** |
|---|---|
| U-Cp(Ctr) | U1, 2.705(3)<br>U1, 2.679(3)<br>U2, 2.671(3)<br>U2, 2.683(3)<br>U3, 2.684(3)<br>U3, 2.680(3)<br>U4, 2.675(3)<br>U4, 2.683(3) |
| U-N | U1-N1, 2.214(6)<br>U1-N2, 2.199(6)<br>U2, N1, 2.188(6)<br>U2-N2, 2.194(5)<br>U3-N1, 2.190(5)<br>U3-N2, 2.202(5)<br>U4-N1, 2.207(5)<br>U4-N2, 2.204(5) |
| U-L | - |
| Cp(Ctr)-U-Cp(Ctr) | 119.9 |

**Table S6.** Comparison of the metrics with similar $N_2$, end-on, and side-on uranium complexes

| | U (**2**) | U-N=N-Mo[a] | U-N=N[b] | U-($\mu$-$\eta^2$:$\eta^2$-$N_2$)-U[c] | U-($\mu$-$\eta^2$:$\eta^2$-$N_2$)-U[d] | $U_2$($\mu$-N)($\mu$-$\eta^2$:$\eta^2$-$N_2$)[e] | $K_2U_2$($\mu$-O)($\mu$-$\eta^2$:$\eta^2$-$N_2$)[f] | $Rb_2U_2$($\mu$-O)($\mu$-$\eta^2$:$\eta^2$-$N_2$)[g] | $K_3U_2$($\mu$-N) $U_2$($\mu$-N)($\mu$-$\eta^2$:$\eta^2$-$N_2$)[h] |
|---|---|---|---|---|---|---|---|---|---|
| U-N end-on | 2.004(9) | 2.220(9) | 2.492(10) | - | - | - | - | - | - |
| U-N side-on | 2.263(16),<br>2.229(15) | - | - | 2.439(5),<br>2.445(5),<br>2.394(5),<br>2.397(5) | 2.401(8),<br>2.422(8),<br>2.402(8),<br>2.423(8) | 2.163 (13)<br>2.311(13) | 2.279(8)<br>2.157(17) | 2.14(3)<br>2.30(1) | 2.178(15)<br>2.318(15) |
| N-N | - | - | - | - | - | 1.521(18) | 1.40(1) | 1.41(1) | 1.54(2) |
| N-N end-on | 1.334(18) | 1.232(11) | 1.120(14) | - | - | - | - | - | - |
| N-N side-on | 1.44(5) | - | - | 1.109(7) | 1.232(10) | - | - | - | - |
| M-M | 5.342(8), 4.26(3) | - | - | - | - | - | 3.3801(5) | - | - |
| U-L(ave) | 2.507,2.512,<br>2.748,<br>2.507,2.512 | - | - | - | - | - | | | -<br>- |
| L-U-L | 140, 118 | - | - | - | - | 106.0 (5) | 99.0(2)<br>98.56(18) | | - |

All values are reported in Å ($1\cdot10^{-10}$ m) [a.] Odom L. A., Arnold P. L., Cummins C.C., *J. Am. Chem. Soc.* **1998**, *120*, 5836-5837. [b.] Evans W. J., Kozimor S. A., Ziller J. W., *J. Am. Chem. Soc.* **2003**, *125*, 14264-14265. [c.] Roussel P., Scott P., *J. Am. Chem. Soc.* **1998**, *120*, 1070-1071. [d.] Geoffey F., Cloke N., Hitchcock P. B., *J. Am. Chem. Soc.* **2002**, *124*, 9352-9353. [e.] M. Falcone, L. Chatelain, R. Scopelliti, I. Živković, M. Mazzanti, *Nature* **2017**, *547*, 332-335. [f.] M. Falcone, L. Barluzzi, J. Andrez, F. Fadaei Tirani, I. Zivkovic, A. Fabrizio, C. Corminboeuf, K. Severin, M. Mazzanti, *Nat. Chem.* **2019**, *11*, 154-160. [g.] N. Jori, T. Rajeshkumar, R. Scopelliti, I. ˇivković, A. Sienkiewicz, L. Maron, M. Mazzanti, *Chem. Sci.* **2022**, *13*, 9232-9242. [h] M. Keener, F. Fadaei-Tirani, R. Scopelliti, I. Zivkovic, M. Mazzanti, *Chem. Sci.* **2022**, *13*, 8025-8035.

**Table S7.** Comparison of the metrics with similar $N_2$, end-on and side-on f-element complexes

| | Gd[a] | Tb[a] | Dy[a] | Lu[c] | Sc[b] | Tb[b] | Gd[b] |
|---|---|---|---|---|---|---|---|
| M-N end-on | 2.325(4) | 2.296(4) | 2.268(7) | 2.184(5) | 2.031(1) | 2.189(2) | 2.268(27), 2.302(26) |
| M-N side-on | - | - | - | - | - | - | 2.408(26), 2.45(3) |
| N-N end-on | 1.130(8) | 1.175(8) | 1.215(13) | 1.203(11) | 1.221(3) | 1.217(12) | 1.271(12), 1.234(9) |
| N-N side-on | - | - | - | | - | - | 1.190(5), 1.193(9) |
| M-M | 5.7791(9) | 5.7659(9) | 5.7500(9) | 5.5711(7) | - | - | - |
| M-L(ave) | 2.4370(16), 2.4299(17) | 2.4146(19), 2.419(2) | 2.405(3), 2.406(3) | 2.336, 2.338 | - | - | - |
| L-M-L | 144.63(8) | 143.96(9) | 143.86(13) | 165.44 | - | - | - |

All values are reported in Å ($1\cdot10^{-10}$ m) [a.] Mondal A., Price C. G. T., Tang J., Layfield R. A., *J. Am. Chem. Soc.* **2023**, *145*, 20121-20131. [b.] Ryan A. J., Balasubarmani S. G., Ziller J. W., Furche F., Evans W. J., *J. Am. Chem. Soc.* **2020**, *142*, 9302-9313. [c.] Papangelis E., Demonti L., del Rosal I., Shephard A., Maron L., Nocton G., Simler T., *J. Am. Chem. Soc.* **2025**, *147*, 11, 9752-9763.

### 7.e. Crystal structure $[Cp^{ttt}_2U(\mu\text{-}I)(\mu_3\text{-}N)(\mu\text{-}N)(UCp^{ttt})_2]$ (3)

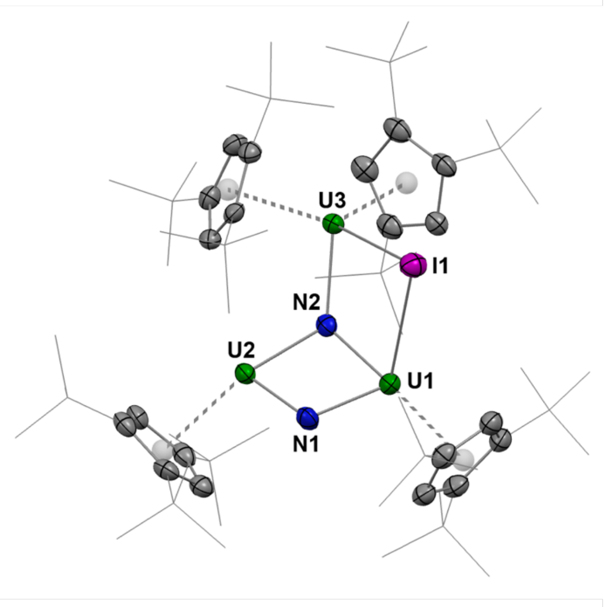


**Figure S60.** ORTEP of **3** showing atom numbering scheme for relevant atoms. Hydrogen atoms, disorder on the *tert*-butyl groups, and lattice toluene are omitted for clarity. Thermal ellipsoids are drawn at the 50% probability level.

### 7.f. Crystal structure of $[Cp^{ttt}_2U(\mu\text{-}N)_2\{U(Cp^{ttt})(OEt_2)\}]$ (4)

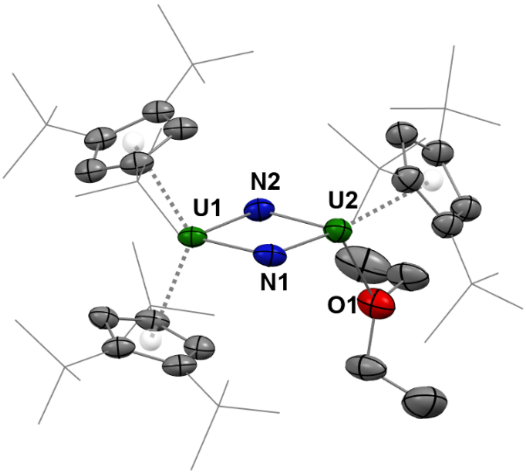


**Figure S61.** ORTEP diagram of **4** showing atom numbering scheme for relevant atoms. Hydrogen atoms are omitted for clarity. Thermal ellipsoids are drawn at the 50% probability level.

### 7.g. Crystal structure of [Cp$^{ttt}{}_2$U(μ-I)(μ$_3$-N)(μ-NH)(UCp$^{ttt}$)$_2$] (5)

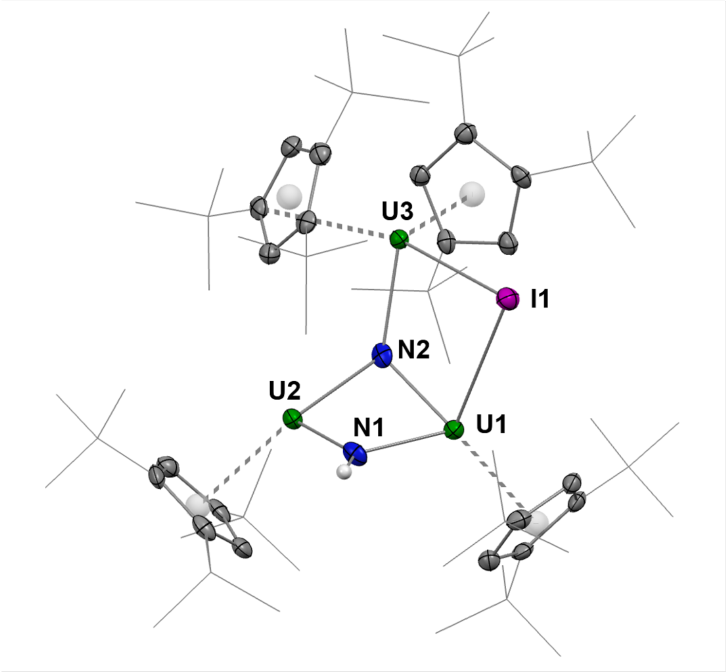


**Figure S62.** ORTEP diagram of **5** showing atom numbering scheme for relevant atoms. Hydrogen atoms (excluding N-*H* atom) and lattice toluene are omitted for clarity. Thermal ellipsoids are drawn at the 50% probability level.

### 7.h. Crystal structure of [(Cp$^{ttt}$$_2$U)$_2$(μ-NH)$_2$] (A)

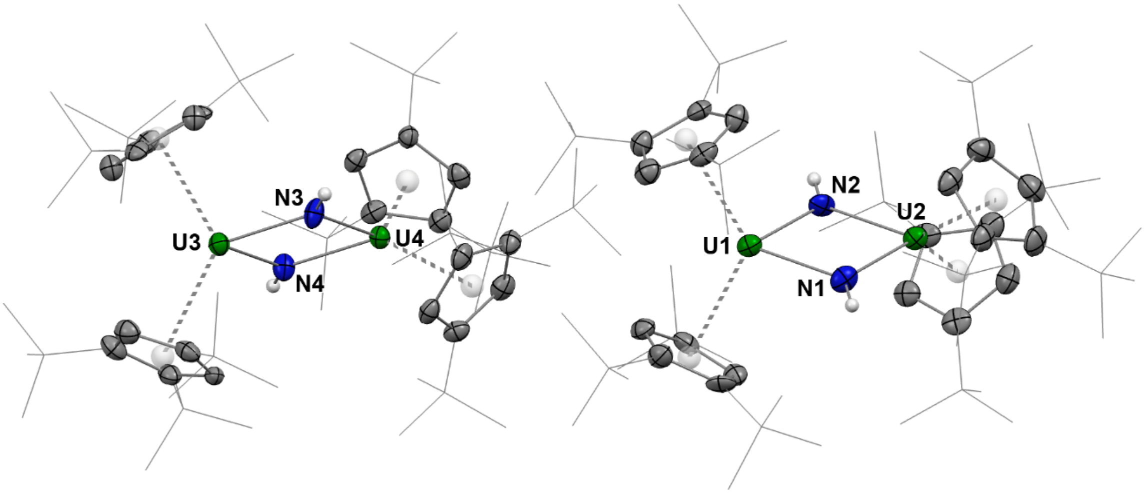


**Figure S63.** ORTEP diagram of **A** showing atom numbering scheme for relevant atoms. Hydrogen atoms (excluding N-*H* atoms) and lattice pentane are omitted for clarity. Thermal ellipsoids are drawn at the 50% probability level.

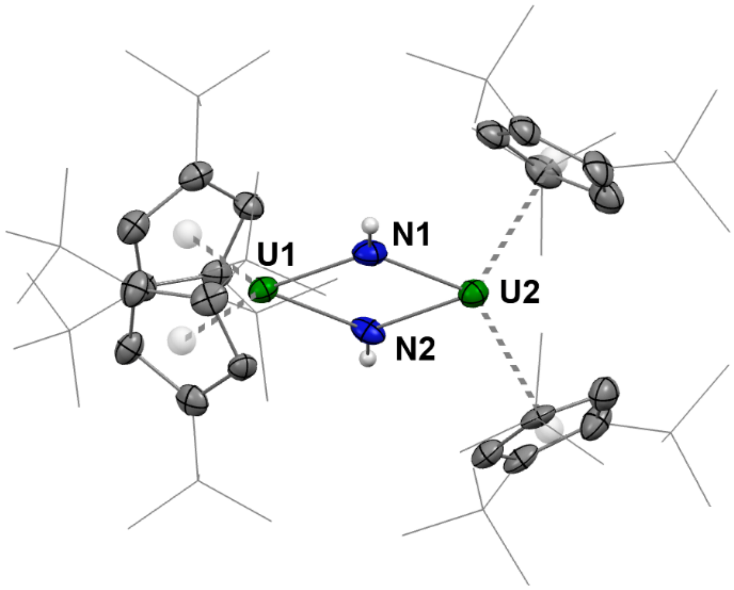


**Figure S64.** ORTEP diagram of the first of the two independent molecules of **A**. Hydrogen atoms (excluding N-*H* atoms) and lattice pentane are omitted for clarity. Thermal ellipsoids are drawn at the 50% probability level.

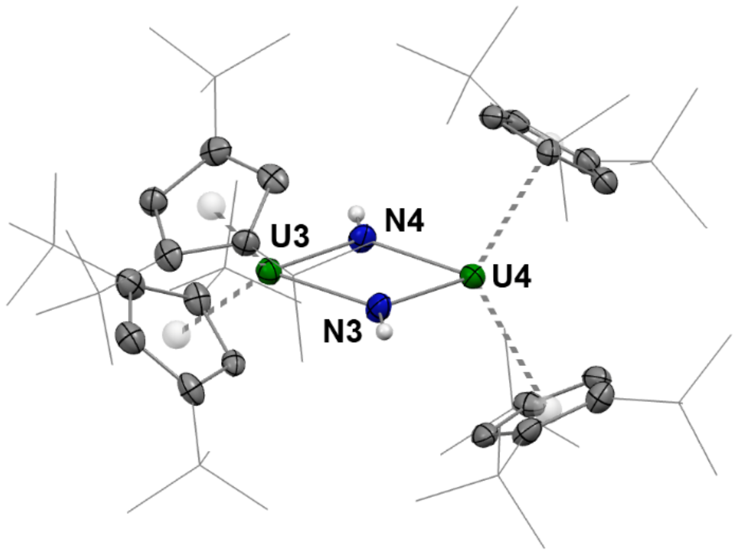


**Figure S65.** ORTEP diagram of the second of the two independent molecules of **A**. Hydrogen atoms (excluding N-*H* atoms) and lattice pentane are omitted for clarity. Thermal ellipsoids are drawn at the 50% probability level.

## 8. Computational details

The optimization of three different spin states for uranium complexes was carried out by employing DFT hybrid functional (B3PW91)[11] along with a small core pseudopotential Stuttgart basis set for uranium and iodine atoms with additional polarization functions for iodine atom.[12] Pople basis sets (6-31G**) were employed for the rest of the atoms.[13] Frequency calculations were performed to locate minima for the optimized structures. All the calculations were performed using Gaussian 09 suite of programs.[14]

**Table S8.** Spin energetics for two spin states computed for $[(Cp^{ttt}{}_2U)_2(\mu\text{-}\eta^1{:}\eta^1\text{-}N_2)]$, **2-end-on**

| spin states | ΔH(ΔG), kcal/mol |
|---|---|
| s=0 (open shell singlet) | 0.0 (0.0) |
| s=2 | 2.0 (0.8) |
| s=3 | 7.1 (4.5) |

**Table S9.** Computed natural charges for selected atoms in $[(Cp^{ttt}{}_2U)_2(\mu\text{-}\eta^1{:}\eta^1\text{-}N_2)]$, **2-end-on**, s=0 (open shell singlet)

| Atom labels | Natural charges |
|---|---|
| U1 | 1.46494 |
| U2 | 1.46570 |
| N3 | -0.65857 |
| N4 | -0.65869 |

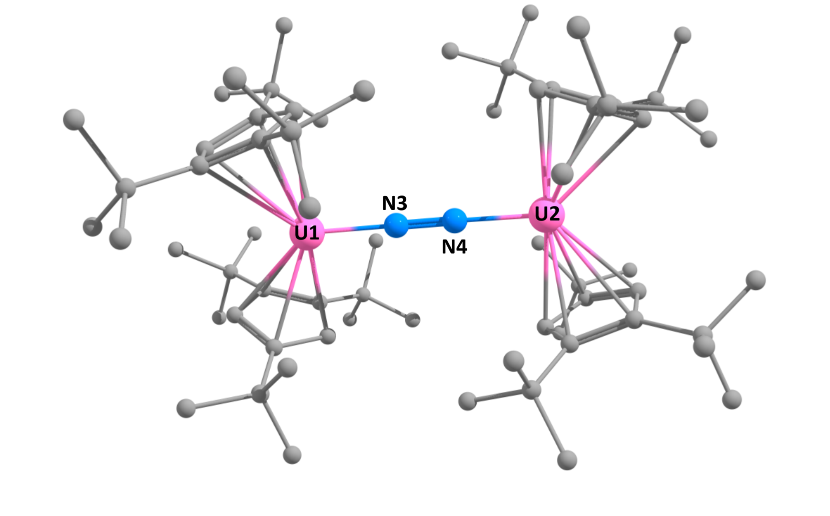

**Table S10.** Computed Wiberg bond index between selected atoms in $[(Cp^{ttt}{}_2U)_2(\mu\text{-}\eta^1\text{:}\eta^1\text{-}N_2)]$, **2-end-on**, s=0 (open shell singlet)

| Atom labels | Wiberg bond index | Atom labels | Wiberg bond index | Atom labels | Wiberg bond index |
|---|---|---|---|---|---|
| U1 | 0.0000 | U2 | 0.0000 | N3 | 0.0000 |
| N3 | 1.5219 | N4 | 1.5218 | N4 | 1.2575 |

**Table S11.** Bonding orbitals (Alpha molecular orbital, AMO) between uranium and nitrogen atoms in $[(Cp^{ttt}{}_2U)_2(\mu\text{-}\eta^1\text{:}\eta^1\text{-}N_2)]$, **2-end-on**, s=0 (open shell singlet)

**(0.99037) BD (1) U(1)-N(3)**
(12.43%) 0.3526* U(1) s(2.57%) p 0.39(0.99%) d 26.34(67.63%) f 11.20(28.76%) g0 .02(0.06%)
(87.57%) 0.9358* N(3) s(64.92%) p 0.54( 35.08%) d 0.00( 0.01%)
**(0.96969) BD (2) U(1)-N(3)**
(40.35%) 0.6352* U(1) s(0.01%) p 1.00(0.03%) d99.99(18.40%) f99.99( 81.55%) g 0.26(0.01%)
(59.65%) 0.7724* N(3) s(0.01%) p 1.00(99.98%) d 0.00(0.01%)
**(0.96217) BD (3) U(1)-N(3)**
(28.11%) 0.5302* U(1) s(0.01%) p 1.00(0.08%) d99.99(59.38%) f99.99(40.52%) g 0.15( 0.01%)
(71.89%) 0.8479* N(3) s(0.03%) p99.99(99.96%) d0.22(0.01%)
**(0.99030) BD (1) U(2)-N(4)**
(11.66%) 0.3415* U(2) s(2.57%) p 0.46(1.17%) d25.79(66.19%) f11.69(30.00%) g 0.03(0.07%)
(88.34%) 0.9399* N(4) s(66.69%) p 0.50(33.31%) d 0.00(0.00%)
**(0.94193) BD (2) U(2)-N(4)**
(17.96%) 0.4238* U(2) s(0.00%) p 1.00(0.07%) d99.99(60.22%) f99.99(39.69%) g 0.36(0.02%)
(82.04%) 0.9058* N(4) s(0.00%) p 1.00(100.00%) d 0.00(0.00%)
**(0.89492) BD (3) U(2)-N(4)**
(15.86%) 0.3982* U(2) s(0.00%) p 1.00(0.03%) d99.99(44.23%) f99.99(55.71%) g0.99(0.03%)
(84.14%) 0.9173* N(4) s(0.00%) p 1.00(100.00%) d 0.00(0.00%)
**(0.99315) BD (1) N(3)-N(4)**
(49.63%) 0.7045* N(3) s( 34.80%) p1.87(65.09%) d 0.00(0.11%)
(50.37%) 0.7097* N(4) s( 33.14%) p 2.01(66.75%) d 0.00(0.10%)

**Table S12.** NBO Second order perturbation analysis (AMO) for $[(Cp^{ttt}{}_2U)_2(\mu\text{-}\eta^1{:}\eta^1\text{-}N_2)]$, **2-end-on**, s=0 (open shell singlet).

| Donor NBO | Acceptor NBO | E(2) kcal/mol |
|---|---|---|
| **(0.99037) BD (1) U(1)-N(3)**<br><br>(12.43%) 0.3526* U(1) s(2.57%) p 0.39(0.99%) d26.34(67.63%) f11.20(28.76%) g 0.02( 0.06%)<br><br>(87.57%) 0.9358* N(3) s( 64.92%) p 0.54(35.08%) d 0.00( 0.01%) | **(0.04580) BD*(1) U(2)- N(4)**<br><br>(88.34%) 0.9399* U(2) s(2.57%) p 0.46(1.17%) d25.79(66.19%) f11.69(30.00%) g 0.03( 0.07%)<br><br>(11.66%) -0.3415* N(4) s( 66.69%) p 0.50(33.31%) d 0.00( 0.00%) | 3.71 |
| **(0.96969) BD (2) U(1)-N(3)**<br><br>(40.35%) 0.6352* U(1) s(0.01%) p 1.00(0.03%) d99.99(18.40%) f99.99(81.55%) g 0.26( 0.01%)<br><br>(59.65%) 0.7724* N(3) s(0.01%) p 1.00(99.98%) d 0.00(0.01%) | **(0.03281) BD*(3) U(2)-N(4)**<br><br>(84.14%) 0.9173* U 2 s( 0.00%)p 1.00( 0.03%)d99.99( 44.23%)f99.99( 55.71%)g 0.99( 0.03%)<br><br>(15.86%) -0.3982* N(4) s(0.00%)p 1.00(100.00%) d 0.00(0.00%) | 3.89 |
| **(0.99030) BD (1) U(2)-N(4)**<br><br>(11.66%) 0.3415* U(2) s(2.57%) p 0.46(1.17%) d25.79(66.19%) f11.69(30.00%) g 0.03( 0.07%)<br><br>(88.34%) 0.9399* N(4) s(66.69%) p 0.50( 33.31%) d 0.00( 0.00%) | **(0.05024) BD*(1) U(1)-N(3)**<br><br>(87.57%) 0.9358* U(1) s( 2.57%) p 0.39(0.99%) d26.34( 67.63%)f 11.20(28.76%) g 0.02(0.06%)<br><br>(12.43%) -0.3526* N(3) s( 64.92%) p 0.54(35.08%) d 0.00( 0.01%) | 4.07 |
| **(0.94193) BD (2) U(2)-N(4)**<br><br>(17.96%) 0.4238* U(2) s( 0.00%) p 1.00(0.07%) d99.99(60.22%) f99.99(39.69%) g 0.36(0.02%)<br><br>(82.04%) 0.9058* N(4) s(0.00%) p1.00(100.00%) d 0.00(0.00%) | **(0.06205) BD*(3) U(1)-N(3)**<br><br>(71.89%) 0.8479* U(1) s(0.01%) p 1.00(0.08%) d99.99( 59.38%) f99.99(40.52%) g0.15(0.01%)<br><br>(28.11%) -0.5302* N(3) s(0.03%) p99.99(99.96%) d 0.22( 0.01%) | 9.35 |

| | | |
|---|---|---|
| **(0.89492) BD (3) U(2)-N(4)**<br><br>(15.86%) 0.3982* U(2) s(0.00%) p 1.00(0.03%) d99.99(44.23%) f99.99(55.71%) g 0.99(0.03%)<br><br>(84.14%) 0.9173* N(4 s(0.00%) p1.00(100.00%) d 0.00( 0.00%) | **(0.12963) BD*(2) U(1)-N(3)**<br><br>(59.65%) 0.7724* U(1) s(0.01%) p 1.00(0.03%) d99.99(18.40%) f99.99(81.55%) g 0.26( 0.01%)<br><br>(40.35%) -0.6352* N(3) s(0.01%) p 1.00(99.98%) d 0.00( 0.01%) | 17.71 |

**Table S13.** Computed MOs for $[(Cp^{ttt}{}_2U)_2(\mu\text{-}\eta^1\text{:}\eta^1\text{-}N_2)]$, **2-end-on**, s=0 (open shell singlet). (a)αMO-HOMO-3 (b) αMO-HOMO-2 (c) αMO-HOMO-1 (d) αMO-HOMO (e) αMO-LUMO (f)βMO-HOMO-3 (g) βMO-HOMO-2 (h) βMO-HOMO-1 (i) βMO-HOMO (j) βMO-LUMO (k)spin density plot

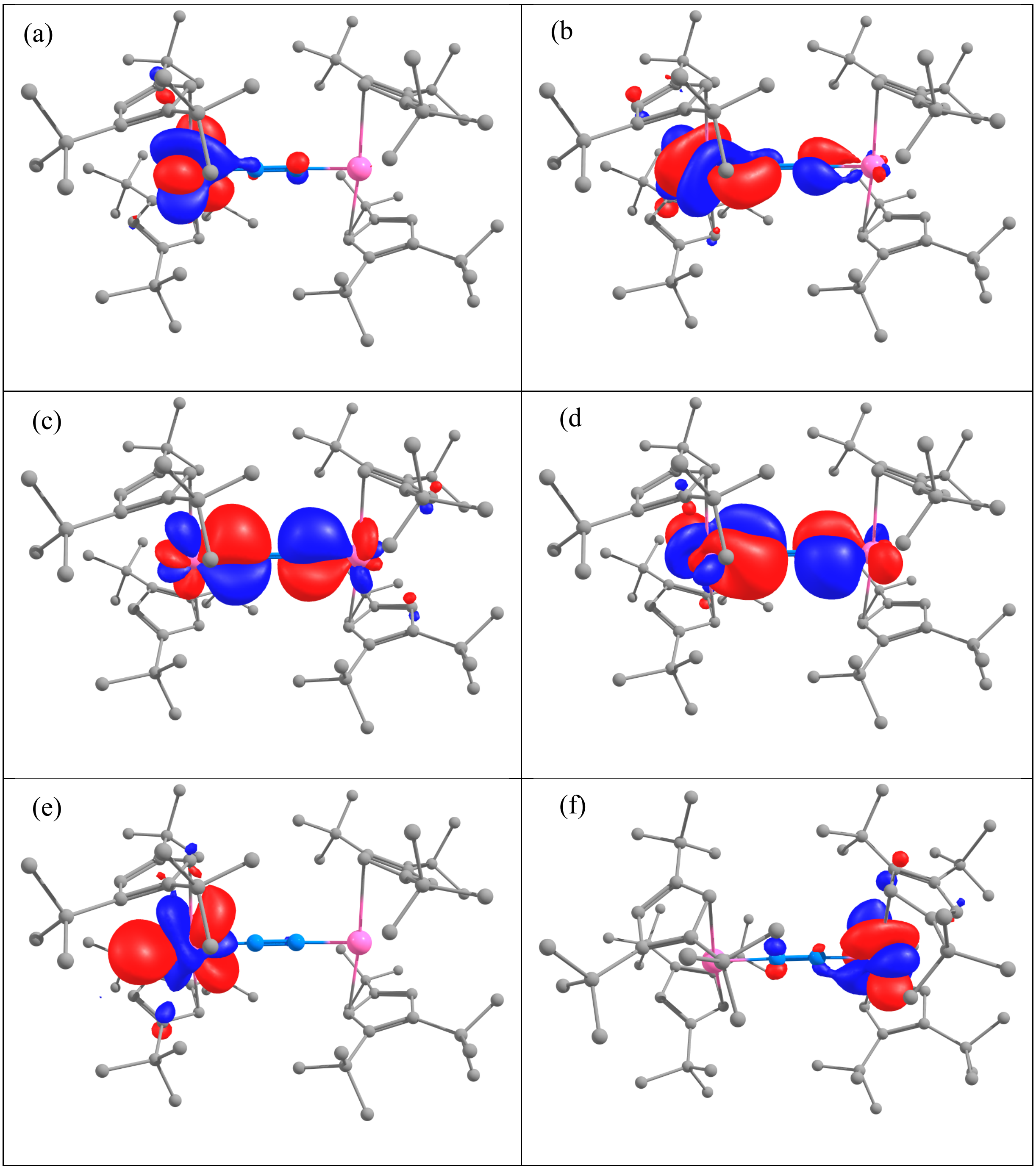

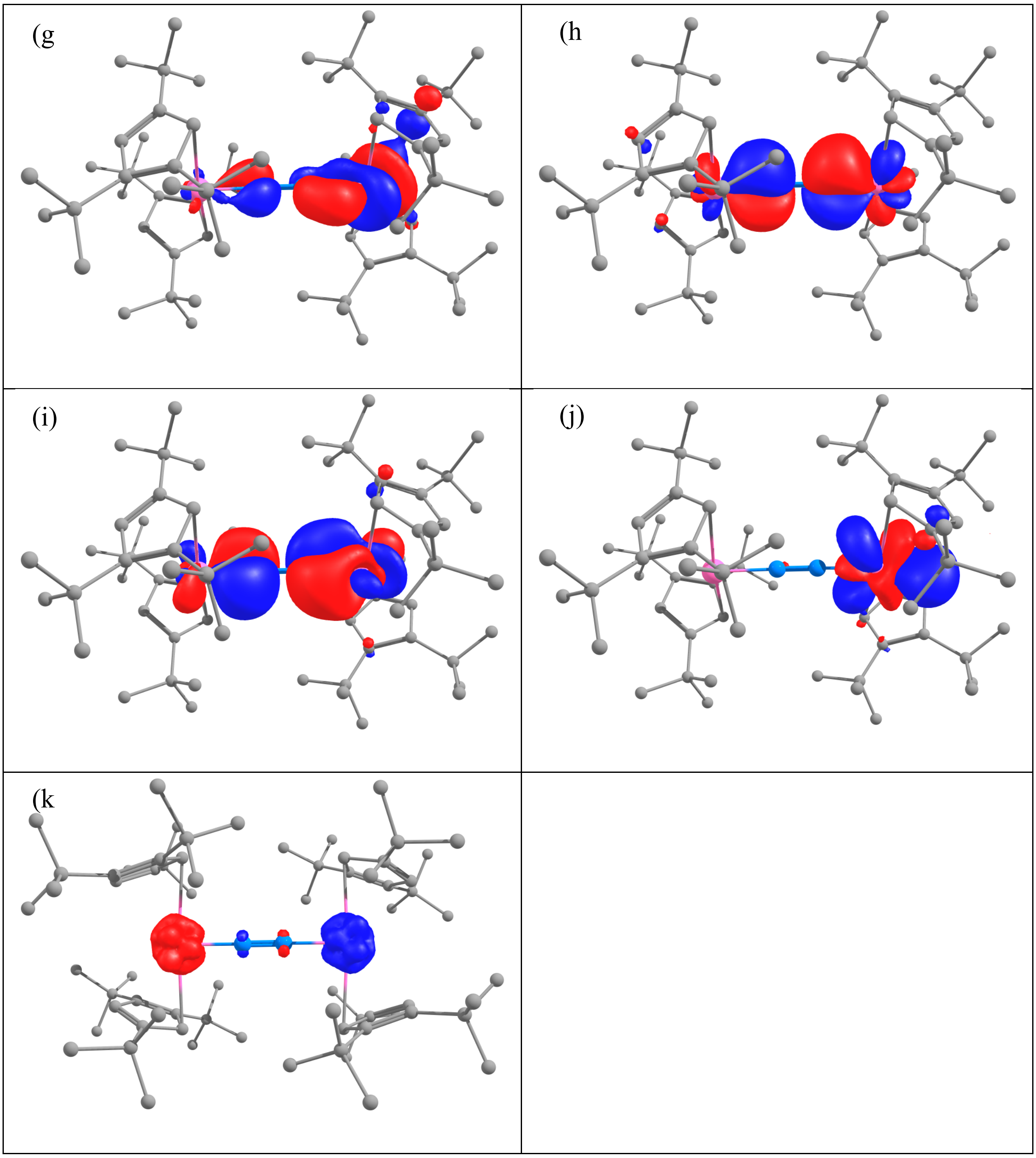
(g
(h
(i)
(j)
(k

**Table S14.** Computed Bond Critical Point (BCP) descriptors for [(Cp$^{ttt}{}_2$U)$_2$(μ-η$^1$:η$^1$-N$_2$)], **2-end-on**, s=0 (open shell singlet).

| | $\rho$(r) | $\nabla^2\rho$(r) | $G$(r) | $V$(r) | $H$(r) | $\varepsilon$ |
|---|---|---|---|---|---|---|
| U(1)-N(3) | 0.14 | 0.51 | 0.19 | -0.26 | -0.07 | 0.25 |
| U(2)-N(4) | 0.14 | 0.51 | 0.19 | -0.26 | -0.07 | 0.25 |
| N(3)-N(4) | 0.39 | -0.82 | 0.22 | -0.64 | -0.42 | 0.01 |

**Figure S66.** Laplacian distribution in U$_2$N$_2$ core of [(Cp$^{ttt}{}_2$U)$_2$(μ-η$^1$:η$^1$-N$_2$)], **2-end-on**, s=0 (open shell singlet).

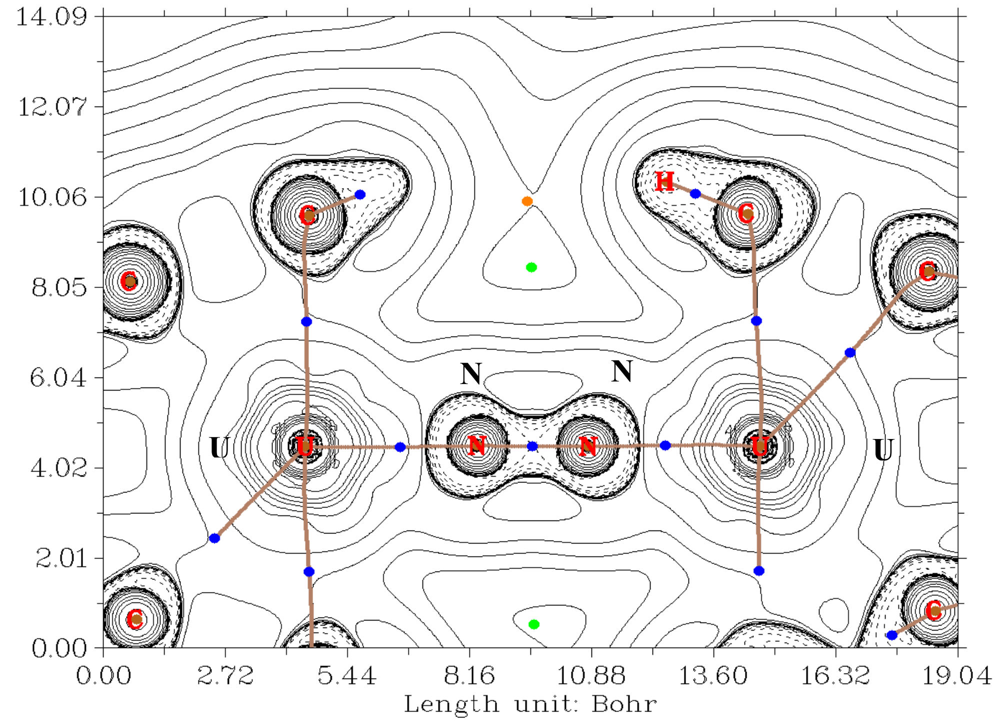

**Table S15.** Computed natural charges for selected atoms in $[(Cp^{ttt}{}_2U)_2(\mu\text{-}\eta^1{:}\eta^1\text{-}N_2)]$, **2-end-on**, s=2

| Atom labels | Natural charges |
|---|---|
| U1 | 1.49093 |
| N2 | -0.69738 |
| N95 | -0.69770 |
| U96 | 1.49292 |
| 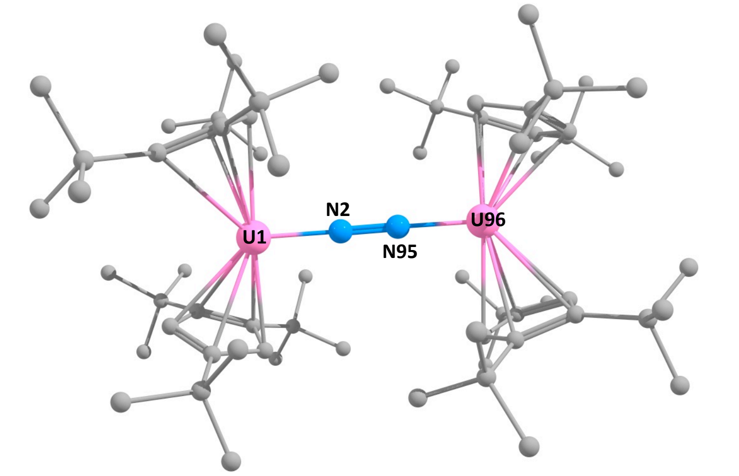 | |



**Table S16.** Computed Wiberg bond index between selected atoms in $[(Cp^{ttt}{}_2U)_2(\mu\text{-}\eta^1{:}\eta^1\text{-}N_2)]$, **2-end-on**, s=2

| Atom labels | Wiberg bond index | Atom labels | Wiberg bond index | Atom labels | Wiberg bond index |
|---|---|---|---|---|---|
| U(1) | 0.0000 | U96 | 0.0000 | N2 | 0.0000 |
| N(2) | 1.6173 | N95 | 1.6170 | N95 | 1.1889 |

**Table S17.** Bonding orbitals (Alpha molecular orbital, AMO) between uranium and nitrogen atoms in $[(Cp^{ttt}{}_2U)_2(\mu\text{-}\eta^1\text{:}\eta^1\text{-}N_2)]$, **2-end-on**, s=2

**(0.99024) BD (1) U(1)-N(2)**

(13.07%) 0.3615* U(1) s( 2.11%) p 0.51( 1.09%) d30.89( 65.33%) f14.85( 31.41%) g 0.03(0.06%)

(86.93%) 0.9324* N(2) s(65.75%) p 0.52(34.24%) d 0.00( 0.01%)

**(0.95336) BD (2) U(1)-N(2)**

(27.68%) 0.5261* U(1) s(0.01%) p 1.00( 0.04%) d99.99( 48.82%) f99.99(51.11%) g 0.33( 0.01%)

(72.32%) 0.8504* N(2) s(0.01%) p 1.00(99.99%) d 0.00( 0.01%)

**(0.93375) BD (3) U(1)-N(2)**

(28.60%) 0.5348* U(1) s(0.00%) p 1.00(0.04%) d99.99(33.75%) f99.99(66.20%) g 0.37(0.01%)

(71.40%) 0.8450* N(2) s(0.00%) p 1.00(99.99%) d 0.00(0.00%)

**(0.99263) BD (1) N(2)- N(95)**

( 50.00%) 0.7071* N 2 s( 34.00%)p 1.94( 65.89%)d 0.00( 0.11%)

( 50.00%) 0.7071* N 95 s( 34.02%)p 1.94( 65.87%)d 0.00( 0.11%)

**(0.99024) BD (1) N(95)-U(96)**

(86.94%) 0.9324* N(95) s( 65.74%) p 0.52( 34.25%) d 0.00( 0.01%)

( 13.06%) 0.3615* U 96 s( 2.09%)p 0.52( 1.09%)d31.19( 65.31%)f15.02( 31.45%)g 0.03( 0.06%)

**(0.95340) BD (2) N(95)-U(96)**

(72.34%) 0.8505* N(95) s(0.01%) p 1.00( 99.99%) d 0.00(0.01%)

(27.66%) 0.5259* U(96) s(0.01%) p 1.00(0.04%) d99.99(48.81%) f99.99(51.13%) g 0.33(0.01%)

**(0.93369) BD (3) N(95)-U(96)**

(71.41%) 0.8450* N(95) s(0.00%) p 1.00( 99.99%) d 0.00(0.00%)

(28.59%) 0.5347* U(96) s(0.00%) p 1.00(0.04%) d99.99(33.69%) f99.99( 66.26%) g 0.37(0.01%)

**Table S18.** NBO Second order perturbation analysis (AMO) for $[(Cp^{ttt}{}_2U)_2(\mu\text{-}\eta^1{:}\eta^1\text{-}N_2)]$, **2-end-on**, s=2.

| Donor NBO | Acceptor NBO | E(2) kcal/mol |
|---|---|---|
| **(0.99024) BD (1) U(1)-N(2)**<br><br>(13.07%) 0.3615* U(1) s(2.11%) p 0.51(1.09%) d30.89(65.33%) f14.85( 31.41%) g 0.03(0.06%)<br><br>(86.93%) 0.9324* N(2) s(65.75%) p0.52(34.24%) d 0.00(0.01%) | **(0.04892) BD*(1) N(95)-U(96)**<br><br>(13.06%) 0.3615* N(95) s(65.74%) p0.52(34.25%) d0.00(0.01%)<br><br>(86.94%) -0.9324* U(96) s(2.09%) p 0.52(1.09%) d31.19(65.31%) f15.02(31.45%) g 0.03(0.06%) | 3.85 |
| **(0.95336) BD (2) U(1)-N(2)**<br><br>( 27.68%) 0.5261* U(1) s(0.01%) p 1.00(0.04%) d99.99(48.82%) f99.99(51.11%) g 0.33(0.01%)<br><br>(72.32%) 0.8504* N(2) s(0.01%) p1.00(99.99%) d0.00(0.01%) | **(0.06136) BD*(2) N(95)- U(96)**<br><br>(27.66%) 0.5259* N(95) s(0.01%) p 1.00(99.99%) d0.00(0.01%)<br><br>(72.34%) -0.8505* U(96) s(0.01%) p1.00(0.04%) d99.99 (48.81%) f99.99(51.13%) g 0.33(0.01%) | 3.84 |
| **(0.95336) BD (2) U(1)-N(2)**<br><br>(27.68%) 0.5261* U(1) s(0.01%) p1.00(0.04%) d99.99( 48.82%) f99.99(51.11%) g 0.33(0.01%)<br><br>(72.32%) 0.8504* N(2) s(0.01%) p 1.00(99.99%) d 0.00(0.01%) | **(0.07281) BD*(3) N(95)-U(96)**<br><br>(28.59%) 0.5347* N(95) s(0.00%) p1.00(99.99%) d 0.00(0.00%)<br><br>(71.41%) -0.8450* U(96) s(0.00%) p 1.00(0.04%) d99.99(33.69%) f99.99(66.26%) g 0.37(0.01%) | 3.92 |
| **(0.93375) BD (3) U(1)-N(2)**<br><br>(28.60%) 0.5348* U(1) s(0.00%) p 1.00(0.04%) d99.99(33.75%) f99.99(66.20%) g 0.37(0.01%)<br><br>(71.40%) 0.8450* N(2) s(0.00%) p 1.00(99.99%) d 0.00(0.00%) | **(0.06136) BD*(2) N(95)-U(96)**<br><br>(27.66%) 0.5259* N(95) s(0.01%) p 1.00(99.99%) d 0.00(0.01%)<br><br>(72.34%) -0.8505* U(96) s(0.01%) p 1.00(0.04%) d99.99(48.81%) f99.99(51.13%) g 0.33(0.01%) | 3.84 |

| | | |
|---|---|---|
| **(0.93375) BD (3) U(1)-N(2)**<br><br>(28.60%) 0.5348* U(1) s(0.00%) p 1.00(0.04%) d99.99(33.75%) f99.99(66.20%) g 0.37(0.01%)<br><br>(71.40%) 0.8450* N(2) s(0.00%) p 1.00(99.99%) d 0.00(0.00%) | **(0.07281) BD*(3) N(95)-U(96)**<br><br>(28.59%) 0.5347* N(95) s(0.00%) p 1.00(99.99%) d 0.00(0.00%)<br><br>(71.41%) -0.8450* U(96) s(0.00%) p 1.00(0.04%) d99.99(33.69%)f 99.99(66.26%) g 0.37(0.01%) | 4.93 |
| **(0.99024) BD (1) N(95)-U(96)**<br><br>(86.94%) 0.9324* N(95) s( 65.74%) p 0.52( 34.25%) d 0.00(0.01%)<br><br>(13.06%) 0.3615* U(96) s(2.09%) p 0.52(1.09%) d31.19(65.31%) f15.02(31.45%) g 0.03(0.06%) | **(0.04899) BD*( 1) U(1)-N(2)**<br><br>(86.93%) 0.9324* U(1) s(2.11%) p 0.51(1.09%) d30.89(65.33%) f14.85(31.41%) g 0.03(0.06%)<br><br>(13.07%) -0.3615* N(2) s(65.75%) p 0.52(34.24%) d 0.00(0.01%) | 3.85 |
| **(0.95340) BD (2) N(95)-U(96)**<br><br>(72.34%) 0.8505* N(95) s(0.01%) p1.00( 99.99%) d 0.00(0.01%)<br><br>(27.66%) 0.5259* U(96) s(0.01%) p1.00(0.04%) d99.99( 48.81%) f99.99(51.13%) g 0.33(0.01%) | **(0.06147) BD*(2) U(1)-N(2)**<br><br>(72.32%) 0.8504* U(1) s(0.01%) p 1.00( 0.04%) d99.99(48.82%) f99.99(51.11%) g 0.33(0.01%)<br><br>(27.68%) -0.5261* N(2) s(0.01%) p 1.00(99.99%) d 0.00(0.01%) | 3.84 |
| **(0.95340) BD (2) N(95)- U(96)**<br><br>(72.34%) 0.8505* N(95) s(0.01%) p1.00(99.99%) d 0.00(0.01%)<br><br>(27.66%) 0.5259* U(96) s(0.01%) p1.00( 0.04%) d99.99(48.81%) f99.99(51.13%) g 0.33(0.01%) | **(0.07283) BD*(3) U(1)-N(2)**<br><br>(71.40%) 0.8450* U(1) s(0.00%) p 1.00(0.04%) d99.99(33.75%) f99.99(66.20%) g0.37 0.01%)<br><br>(28.60%) -0.5348* N(2) s(0.00%) p 1.00(99.99%) d 0.00(0.00%) | 3.91 |
| **(0.93369) BD (3) N(95)-U(96)**<br><br>(71.41%) 0.8450* N(95) s(0.00%) p1.00( 99.99%) d 0.00(0.00%) | **(0.06147) BD*(2) U(1)- N(2)**<br><br>(72.32%) 0.8504* U(1) s(0.01%) p 1.00(0.04%) d99.99(48.82%) f99.9(51.11%) g 0.33(0.01%) | 3.84 |

| | | |
|---|---|---|
| (28.59%) 0.5347* U(96) s(0.00%) p1.00(0.04%) d99.99(33.69%) f99.99(66.26%) g 0.37(0.01%) | (27.68%) -0.5261* N(2) s(0.01%) p 1.00(99.99%) d 0.00(0.01%) | |
| **(0.93369) BD (3) N(95)-U(96)**<br><br>(71.41%) 0.8450* N(95) s(0.00%) p1.00( 99.99%) d 0.00(0.00%)<br><br>(28.59%) 0.5347* U(96) s(0.00%) p1.00(0.04%) d99.99(33.69%)f 99.99(66.26%) g 0.37(0.01%) | **(0.07283) BD*(3) U(1)-N(2)**<br><br>(71.40%) 0.8450* U(1) s(0.00%) p1.00(0.04%) d99.99(33.75%) f99.99(66.20%) g 0.37(0.01%)<br><br>(28.60%) -0.5348* N(2) s(0.00%) p 1.00(99.99%) d 0.00(0.00%) | 4.93 |

**Table S19.** DFT computed MOs for $[(Cp^{ttt}{}_2U)_2(\mu\text{-}\eta^1\text{:}\eta^1\text{-}N_2)]$, **2-end-on**, s=2. (a)αMO-HOMO-5 (b) αMO-HOMO-4 (c) αMO-HOMO-3 (d) αMO-HOMO-2 (e) αMO-HOMO-1 (f) αMO-HOMO (g) αMO-LUMO (h)βMO-HOMO-1 (i) βMO-HOMO (j) βMO-LUMO (k) spin density plot

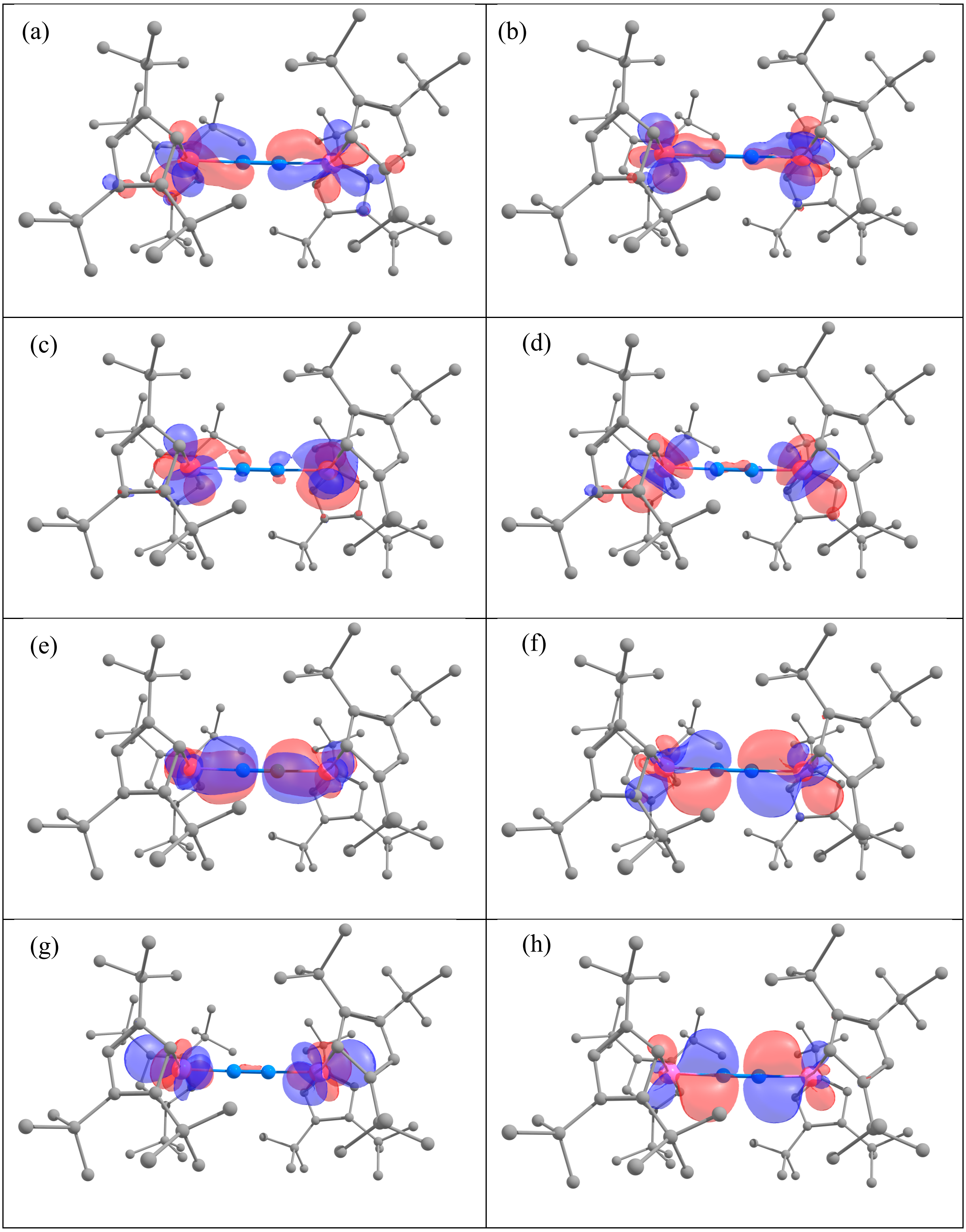

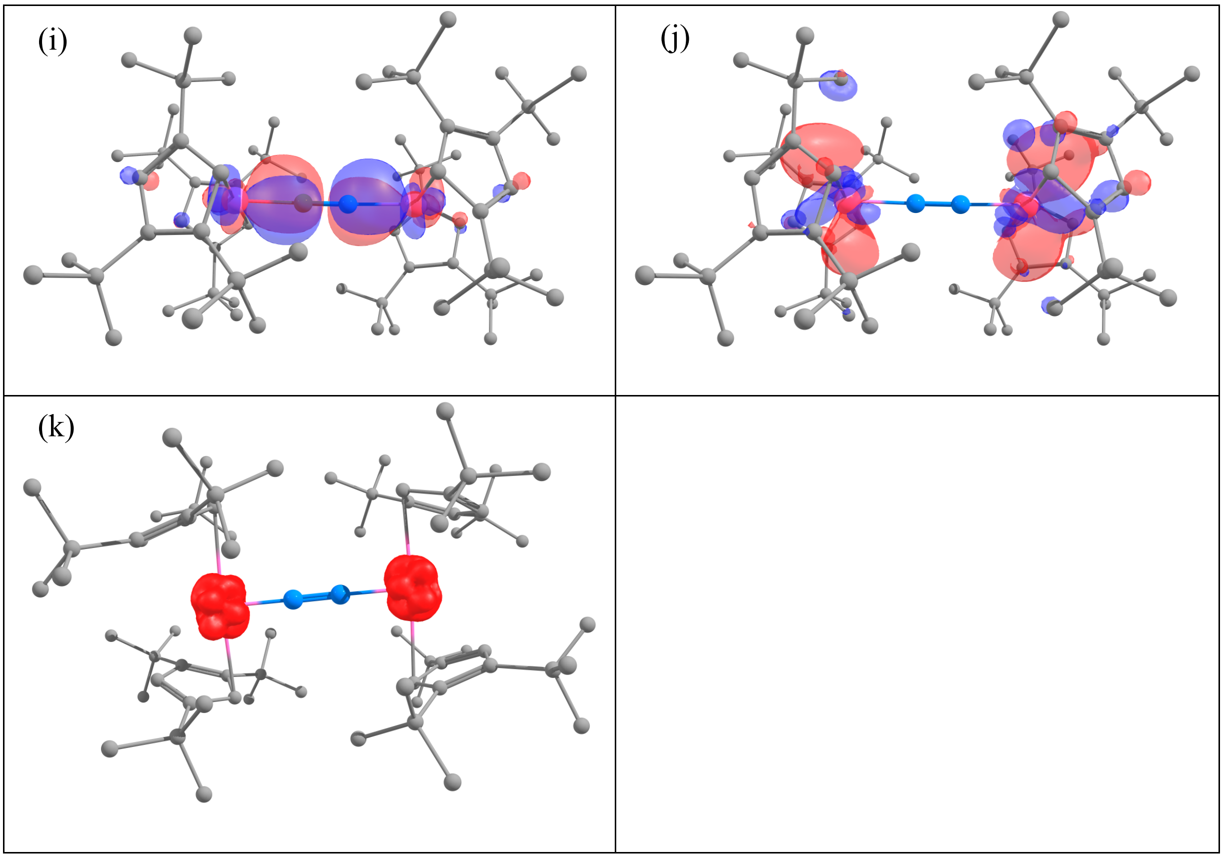
(i)
(j)
(k)

**Table S20.** Computed natural charges for selected atoms in [(Cp$^{ttt}{}_2$U)$_2$(μ-η$^2$:η$^2$-N$_2$)], **2-side-on**, s=2

| Atom labels | Natural charges |
|---|---|
| U1 | 1.39636 |
| U94 | 1.54145 |
| N187 | -0.66834 |
| N188 | -0.66077 |

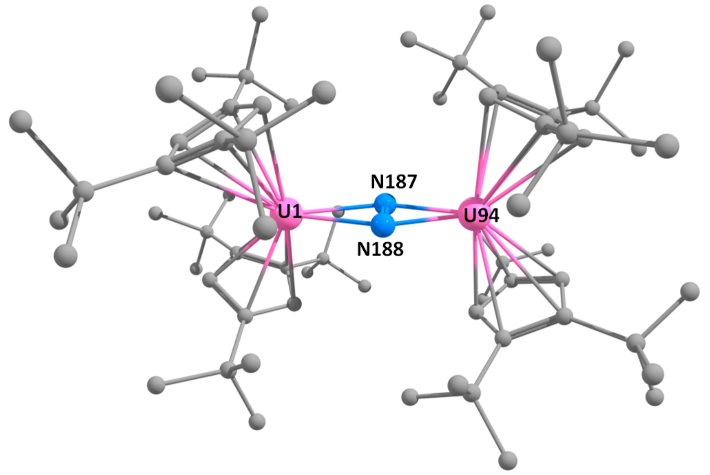


**Table S21.** Computed Wiberg bond index between selected atoms in [(Cp$^{ttt}{}_2$U)$_2$(μ-η$^2$:η$^2$-N$_2$)], **2-side-on**, s=2

| Atom labels | Wiberg bond index | Atom labels | Wiberg bond index | Atom labels | Wiberg bond index |
|---|---|---|---|---|---|
| U1 | 0.0000 | U1 | 0.0000 | N187 | 0.0000 |
| N187 | 0.4993 | N188 | 0.4733 | N188 | 1.3287 |
| Atom labels | Wiberg bond index | Atom labels | Wiberg bond index | | |
| U94 | 0.0000 | U94 | 0.0000 | | |
| N187 | 0.8520 | N188 | 0.8919 | | |

**Table S22.** Bonding orbitals (Alpha molecular orbital, AMO) between uranium and nitrogen atoms in $[(Cp^{ttt}{}_2U)_2(\mu\text{-}\eta^2{:}\eta^2\text{-}N_2)]$, **2-side-on**, s=2

**(0.95906) BD (1) U(94)- N(188)**

(18.67%) 0.4321* U(94) s(0.44%) p1.89(0.84%) d73.47(32.62%) f99.99(66.07%) g 0.06(0.03%)

(81.33%) 0.9018* N(188) s(5.01%) p18.93(94.88%) d0.02(0.10%)

**(0.94771) BD (1) N(187)-N(188)**

(50.18%) 0.7084* N(187) s(25.84%) p2.86(73.86%) d 0.01(0.30%)

(49.82%) 0.7058* N(188) s(23.98%) p 3.16(75.71%) d 0.01(0.31%)

**(0.86440) BD (2) N(187)-N(188)**

(49.31%) 0.7022* N(187) s(0.00%) p1.00(99.67%) d0.00(0.33%)

(50.69%) 0.7119* N(188) s(0.00%) p1.00(99.68%) d0.00(0.32%)

**Table S23.** NBO Second order perturbation analysis (AMO) for [($Cp^{ttt}{}_2U)_2(\mu$-$\eta^2$:$\eta^2$-$N_2$)], **2-side-on**, s=2.

| Donor NBO | Acceptor NBO | E(2) kcal/mol |
|---|---|---|
| (0.88270) LP ( 1) N187<br><br>s( 71.73%)p 0.39( 28.22%)d 0.00( 0.05%) | (0.16481) LV ( 1) U 1<br><br>s( 0.00%)p 1.00( 0.03%)d99.99( 76.02%)f99.99( 23.94%)g 0.23( 0.01%) | 18.49 |
| (0.88270) LP ( 1) N187<br><br>s( 71.73%)p 0.39( 28.22%)d 0.00( 0.05%) | (0.14859) LV ( 2) U 1<br><br>s( 0.57%)p 0.29( 0.17%)d99.99( 90.72%)f15.02( 8.54%)g 0.00( 0.00%) | 4.39 |
| (0.88270) LP ( 1) N187<br><br>s( 71.73%)p 0.39( 28.22%)d 0.00( 0.05%) | (0.13878) LV ( 4) U 1<br><br>s( 0.95%)p 0.44( 0.42%)d97.77( 93.16%)f 5.73( 5.46%)g 0.00( 0.00%) | 15.83 |
| (0.88270) LP ( 1) N187<br><br>s( 71.73%)p 0.39( 28.22%)d 0.00( 0.05%) | (0.06184) LV ( 6) U 1<br><br>s( 63.20%)p 0.01( 0.51%)d 0.42( 26.66%)f 0.15( 9.62%)g 0.00( 0.00%) | 5.58 |
| (0.81017) LP ( 2) N187<br><br>s( 2.63%)p36.94( 97.25%)d 0.04( 0.12%) | (0.16481) LV ( 1) U 1<br><br>s( 0.00%)p 1.00( 0.03%)d99.99( 76.02%)f99.99( 23.94%)g 0.23( 0.01%) | 5.04 |
| (0.88460) LP ( 1) N188<br><br>s( 71.20%)p 0.40( 28.75%)d 0.00( 0.05%) | (0.16481) LV ( 1) U 1<br><br>s( 0.00%)p 1.00( 0.03%)d99.99( 76.02%)f99.99( 23.94%)g 0.23( 0.01%) | 15.57 |
| (0.88460) LP ( 1) N188<br><br>s( 71.20%)p 0.40( 28.75%)d 0.00( 0.05%) | (0.13878) LV ( 4) U 1<br><br>s( 0.95%)p 0.44( 0.42%)d97.77( 93.16%)f 5.73( 5.46%)g 0.00( 0.00%) | 16.02 |
| (0.88460) LP ( 1) N188 | (0.06184) LV ( 6) U 1 | 5.45 |

| | | |
|---|---|---|
| s( 71.20%)p 0.40( 28.75%)d 0.00( 0.05%) | s( 63.20%)p 0.01( 0.51%)d 0.42( 26.66%)f 0.15( 9.62%)g 0.00( 0.00%) | |
| (0.94771) BD ( 1) N187- N188<br>( 50.18%) 0.7084* N187 s( 25.84%)p 2.86( 73.86%)d 0.01( 0.30%)<br>( 49.82%) 0.7058* N188 s( 23.98%)p 3.16( 75.71%)d 0.01( 0.31%) | (0.13878) LV ( 4) U 1<br>s( 0.95%)p 0.44( 0.42%)d97.77( 93.16%)f 5.73( 5.46%)g 0.00( 0.00%) | 14.95 |
| (0.94771) BD ( 1) N187- N188<br>( 50.18%) 0.7084* N187 s( 25.84%)p 2.86( 73.86%)d 0.01( 0.30%)<br>( 49.82%) 0.7058* N188 s( 23.98%)p 3.16( 75.71%)d 0.01( 0.31%) | (0.06184) LV ( 6) U 1<br>s( 63.20%)p 0.01( 0.51%)d 0.42( 26.66%)f 0.15( 9.62%)g 0.00( 0.00%) | 3.40 |
| (0.86440) BD ( 2) N187- N188<br>( 49.31%) 0.7022* N187 s( 0.00%)p 1.00( 99.67%)d 0.00( 0.33%)<br>( 50.69%) 0.7119* N188 s( 0.00%)p 1.00( 99.68%)d 0.00( 0.32%) | (0.07858) LV ( 5) U 1<br>s( 1.06%)p 0.92( 0.98%)d33.09( 34.97%)f59.60( 62.99%)g 0.00( 0.00%) | 7.47 |
| (0.88270) LP ( 1) N187<br>s( 71.73%)p 0.39( 28.22%)d 0.00( 0.05%) | (0.16257) LV ( 3) U 94<br>s( 0.73%)p 0.72( 0.53%)d99.99( 88.30%)f14.38( 10.45%)g 0.00( 0.00%) | 18.71 |
| (0.88270) LP ( 1) N187<br>s( 71.73%)p 0.39( 28.22%)d 0.00( 0.05%) | (0.17171) LV ( 2) U 94<br>s( 0.78%)p 0.40( 0.31%)d99.99( 86.33%)f16.18( 12.58%)g 0.00( 0.00%) | 8.27 |
| (0.88270) LP ( 1) N187<br>s( 71.73%)p 0.39( 28.22%)d 0.00( 0.05%) | (0.02131) LV ( 9) U 94<br>s( 0.75%)p 1.29( 0.96%)d43.97( 32.76%)f87.93( 65.52%)g 0.02( 0.01%) | 5.24 |
| (0.81017) LP ( 2) N187 | (0.17171) LV ( 2) U 94 | 4.96 |

| | | |
|---|---|---|
| s( 2.63%)p36.94( 97.25%)d 0.04( 0.12%) | s( 0.78%)p 0.40( 0.31%)d99.99( 86.33%)f16.18( 12.58%)g 0.00( 0.00%) | |
| (0.81017) LP ( 2) N187<br><br>s( 2.63%)p36.94( 97.25%)d 0.04( 0.12%) | (0.16257) LV ( 3) U 94<br><br>s( 0.73%)p 0.72( 0.53%)d99.99( 88.30%)f14.38( 10.45%)g 0.00( 0.00%) | 9.87 |
| (0.81017) LP ( 2) N187<br><br>s( 2.63%)p36.94( 97.25%)d 0.04( 0.12%) | (0.06006) LV ( 7) U 94<br><br>s( 2.33%)p 1.52( 3.53%)d19.52( 45.46%)f20.87( 48.61%)g 0.03( 0.07%) | 5.93 |
| (0.81017) LP ( 2) N187<br><br>s( 2.63%)p36.94( 97.25%)d 0.04( 0.12%) | (0.11723) BD*( 1) U 94- N188<br><br>( 81.33%) 0.9018* U 94 s( 0.44%)p 1.89( 0.84%)d73.47( 32.62%)f99.99( 66.07%)g 0.06( 0.03%)<br><br>( 18.67%) -0.4321* N188 s( 5.01%)p18.93( 94.88%)d 0.02( 0.10%) | 30.67 |
| (0.88460) LP ( 1) N188<br><br>s( 71.20%)p 0.40( 28.75%)d 0.00( 0.05%) | (0.16257) LV ( 3) U 94<br><br>s( 0.73%)p 0.72( 0.53%)d99.99( 88.30%)f14.38( 10.45%)g 0.00( 0.00%) | 7.15 |
| (0.88460) LP ( 1) N188<br><br>s( 71.20%)p 0.40( 28.75%)d 0.00( 0.05%) | (0.06548) LV ( 6) U 94<br><br>s( 87.40%)p 0.01( 1.01%)d 0.07( 5.74%)f 0.07( 5.85%)g 0.00( 0.01%) | 4.69 |
| (0.88460) LP ( 1) N188<br><br>s( 71.20%)p 0.40( 28.75%)d 0.00( 0.05%) | (0.02131) LV ( 9) U 94<br><br>s( 0.75%)p 1.29( 0.96%)d43.97( 32.76%)f87.93( 65.52%)g 0.02( 0.01%) | 8.56 |
| (0.94771) BD ( 1) N187- N188<br><br>( 50.18%) 0.7084* N187 s( 25.84%)p 2.86( 73.86%)d 0.01( 0.30%) | (0.17171) LV ( 2) U 94<br><br>s( 0.78%)p 0.40( 0.31%)d99.99( 86.33%)f16.18( 12.58%)g 0.00( 0.00%) | 4.77 |

| | | |
|---|---|---|
| ( 49.82%) 0.7058* N188 s( 23.98%)p 3.16( 75.71%)d 0.01( 0.31%) | | |
| (0.94771) BD ( 1) N187- N188<br><br>( 50.18%) 0.7084* N187 s( 25.84%)p 2.86( 73.86%)d 0.01( 0.30%)<br><br>( 49.82%) 0.7058* N188 s( 23.98%)p 3.16( 75.71%)d 0.01( 0.31%) | (0.16257) LV ( 3) U 94<br><br>s( 0.73%)p 0.72( 0.53%)d99.99( 88.30%)f14.38( 10.45%)g 0.00( 0.00%) | 14.22 |
| (0.94771) BD ( 1) N187- N188<br><br>( 50.18%) 0.7084* N187 s( 25.84%)p 2.86( 73.86%)d 0.01( 0.30%)<br><br>( 49.82%) 0.7058* N188 s( 23.98%)p 3.16( 75.71%)d 0.01( 0.31%) | (0.06006) LV ( 7) U 94<br><br>s( 2.33%)p 1.52( 3.53%)d19.52( 45.46%)f20.87( 48.61%)g 0.03( 0.07%) | 13.72 |
| (0.86440) BD ( 2) N187- N188<br><br>( 49.31%) 0.7022* N187 s( 0.00%)p 1.00( 99.67%)d 0.00( 0.33%)<br><br>( 50.69%) 0.7119* N188 s( 0.00%)p 1.00( 99.68%)d 0.00( 0.32%) | (0.11505) LV ( 4) U 94<br><br>s( 0.01%)p 1.00( 0.29%)d99.99( 73.89%)f88.71( 25.81%)g 0.03( 0.01%) | 23.35 |

**Table S24.** DFT computed MOs for $[(Cp^{ttt}{}_2U)_2(\mu\text{-}\eta^2{:}\eta^2\text{-}N_2)]$, **2-side-on**, s=2. (a) αMO-HOMO-13 (b) αMO-HOMO-5 (c) αMO-HOMO-4 (d) αMO-HOMO-3 (e) αMO-HOMO-2 (f) αMO-HOMO-1 (g) αMO-HOMO (h) αMO-LUMO (i) βMO-LUMO-8 (j) β βMO-HOMO (k) βMO-LUMO (l) spin density plot

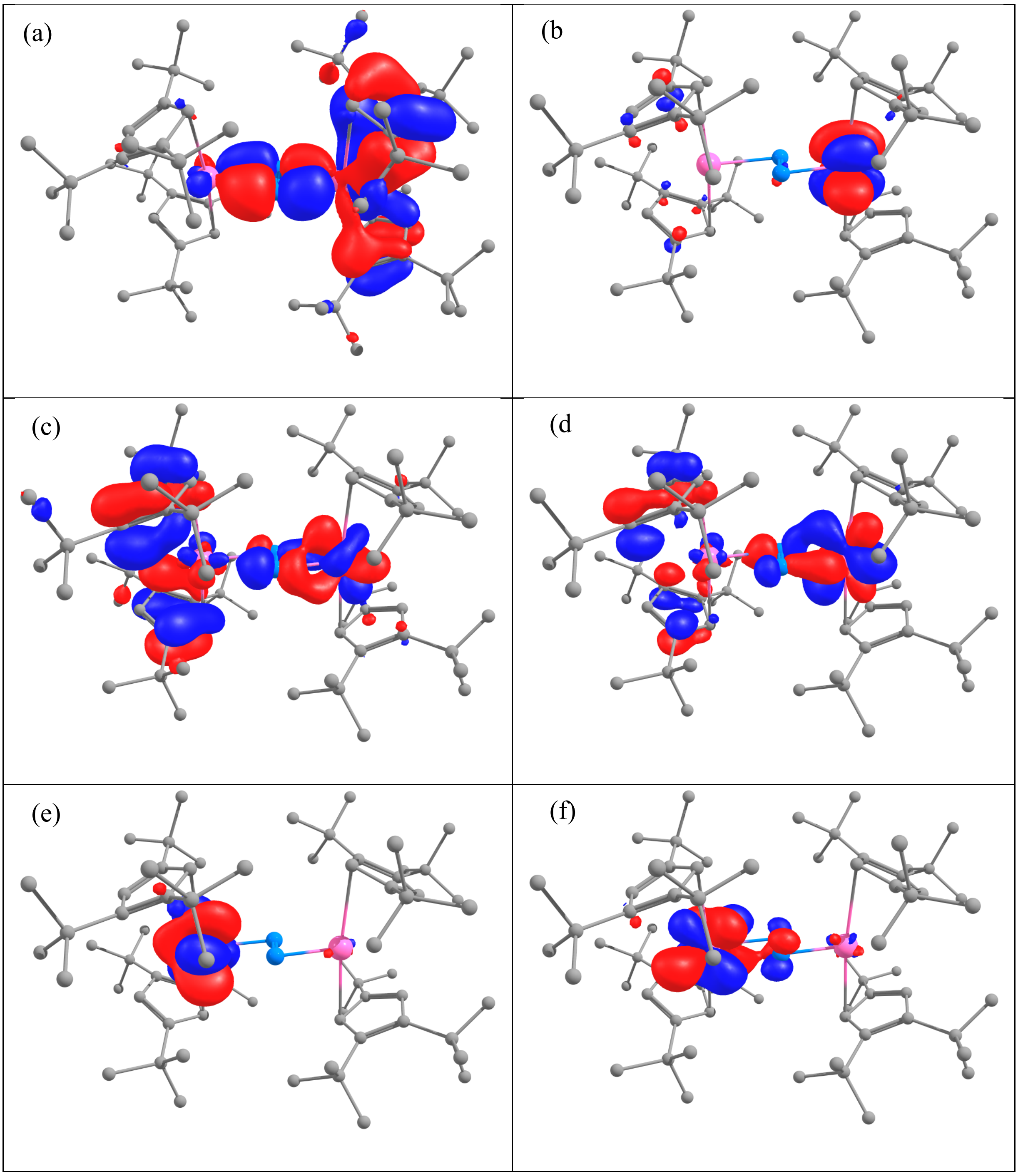

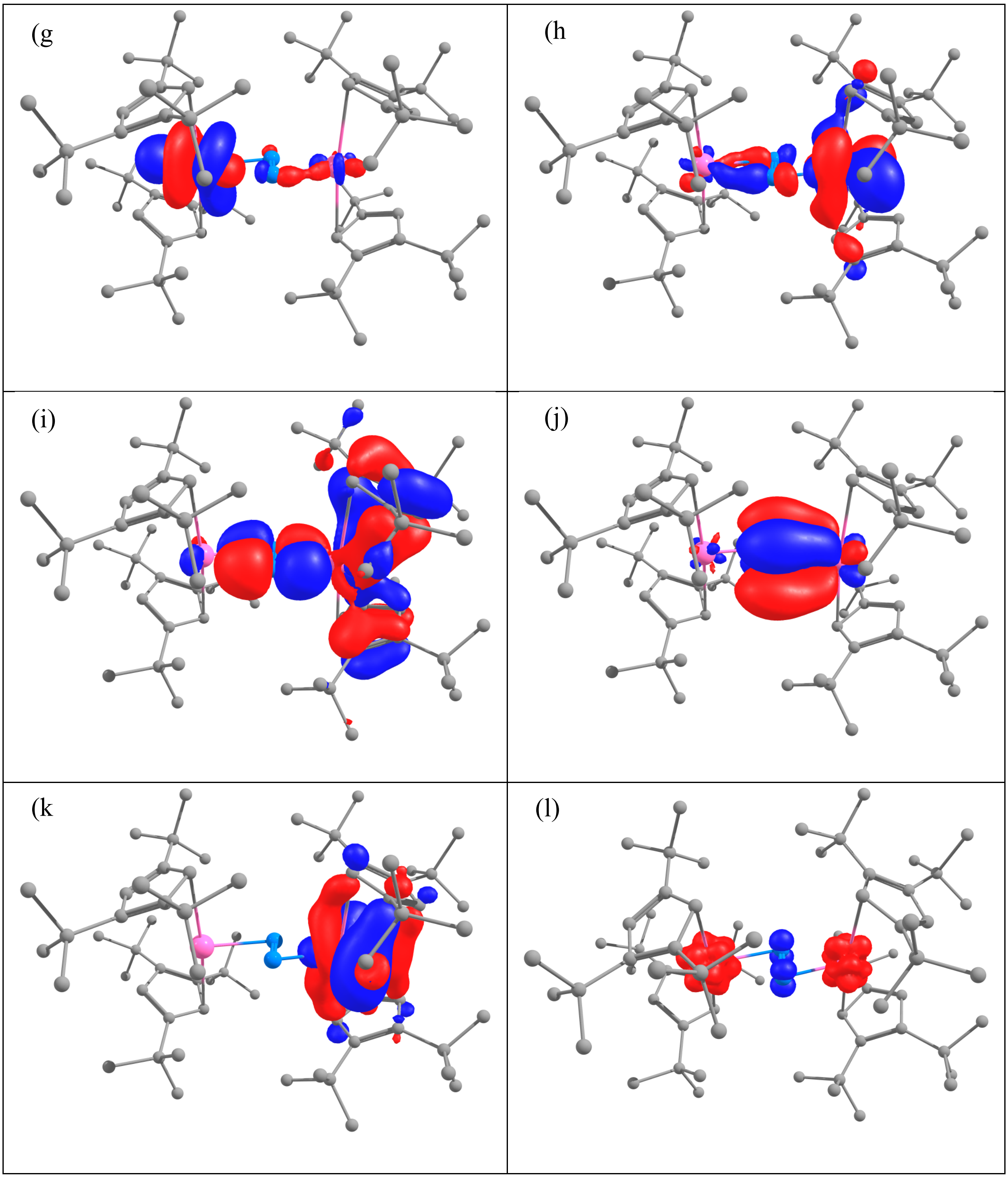
(g
(h
(i)
(j)
(k
(l)

**Table S25.** Computed BCP descriptors for [(Cp$^{ttt}$$_2$U)$_2$(μ-η$^2$:η$^2$-N$_2$)], **2-side-on**, s=2.

| | $\rho$(r) | $\nabla^2\rho$(r) | $G$(r) | $V$(r) | $H$(r) | $\varepsilon$ |
|---|---|---|---|---|---|---|
| U1-N187 | 0.07 | 0.21 | 0.06 | -0.07 | -0.01 | 0.37 |
| U1-N188 | 0.07 | 0.20 | 0.06 | -0.07 | -0.01 | 0.37 |
| U94-N187 | 0.11 | 0.24 | 0.10 | -0.13 | -0.04 | 0.05 |
| U94-N188 | 0.12 | 0.23 | 0.10 | -0.14 | -0.04 | 0.02 |
| N187-N188 | 0.36 | -0.54 | 0.23 | -0.59 | -0.36 | 0.07 |

**Figure S67.** Laplacian distribution in $U_2N_2$ core of [(Cp$^{ttt}$$_2$U)$_2$(μ-η$^2$:η$^2$-N$_2$)], **2-side-on**, s=2.

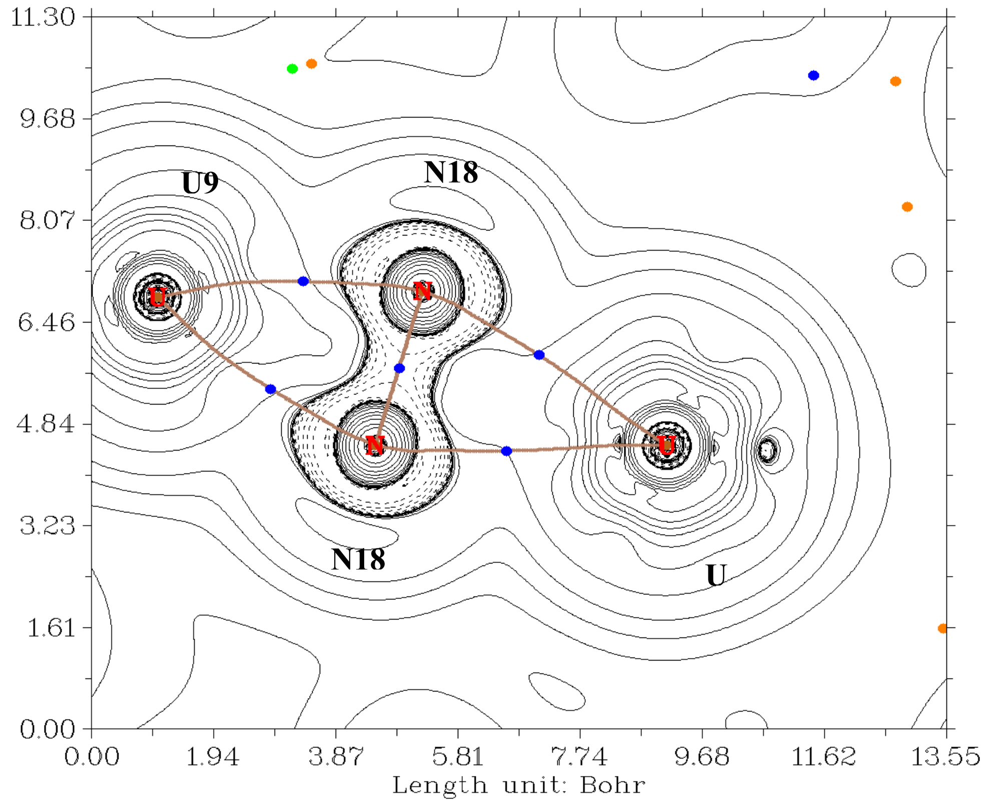

**Table S26.** Spin energetics for two spin states computed for $[Cp^{ttt}{}_2U(\mu\text{-}N)_2\{U(Cp^{ttt})(OEt_2)\}]$, **4**.

| spin states | ΔH(ΔG), kcal/mol |
|---|---|
| s=1/2 | 0.6 (0.3) |
| s=3/2 | 0.0 |

**Table S27.** Computed natural charges for selected atoms in $[Cp^{ttt}{}_2U(\mu\text{-}N)_2\{U(Cp^{ttt})(OEt_2)\}]$, **4,** s=3/2.

| Atom labels | Natural charges |
|---|---|
| U1 | 1.42943 |
| U2 | 1.66571 |
| N3 | -1.03791 |
| N4 | -1.04547 |

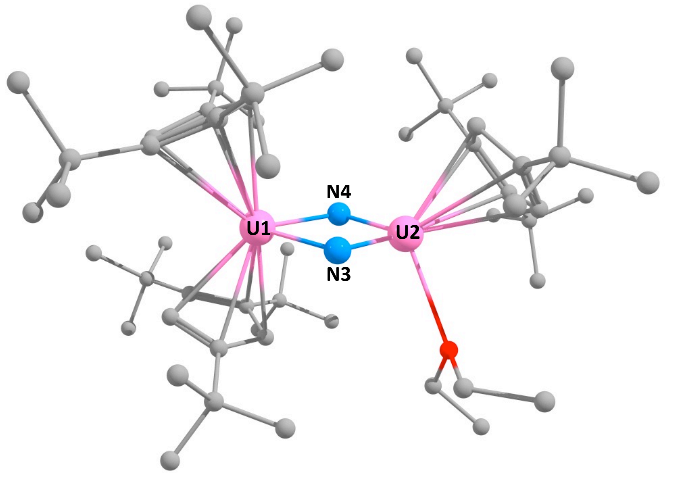

**Table S28.** Computed Wiberg bond index between selected atoms in [$Cp^{ttt}{}_2U(\mu\text{-}N)_2\{U(Cp^{ttt})(OEt_2)\}$], **4**, s=3/2.

| Atom labels | Wiberg bond index | Atom labels | Wiberg bond index | Atom labels | Wiberg bond index |
|---|---|---|---|---|---|
| U1 | 0.0000 | U1 | 0.0000 | N3 | 0.0000 |
| N3 | 0.9667 | N4 | 1.0096 | N4 | 0.0242 |
| Atom labels | Wiberg bond index | Atom labels | Wiberg bond index | | |
| U2 | 0.0000 | U2 | 0.0000 | | |
| N3 | 1.7786 | N4 | 1.7273 | | |

**Table S29.** Bonding orbitals (Alpha molecular orbital, AMO) between uranium and nitrogen atoms in [$Cp^{ttt}{}_2U(\mu\text{-}N)_2\{U(Cp^{ttt})(OEt_2)\}$], **4**, s=3/2.

(0.92579) BD ( 1) U 2- N 3
( 20.49%) 0.4526* U 2 s( 0.14%)p 6.12( 0.88%)d99.99( 36.69%)f99.99( 62.24%)g 0.29( 0.04%)
( 79.51%) 0.8917* N 3 s( 2.89%)p33.51( 96.92%)d 0.06( 0.19%)
(0.89418) BD ( 2) U 2- N 3
( 25.76%) 0.5075* U 2 s( 0.04%)p 2.97( 0.12%)d99.99( 46.30%)f99.99( 53.52%)g 0.54( 0.02%)
( 74.24%) 0.8617* N 3 s( 1.61%)p60.88( 98.23%)d 0.10( 0.16%)
(0.88328) BD ( 3) U 2- N 3
( 23.80%) 0.4879* U 2 s( 0.03%)p 9.63( 0.27%)d99.99( 39.06%)f99.99( 60.61%)g 1.05( 0.03%)
( 76.20%) 0.8729* N 3 s( 5.00%)p18.98( 94.84%)d 0.03( 0.16%)
(0.92666) BD ( 1) U 2- N 4
( 19.11%) 0.4371* U 2 s( 0.16%)p 6.46( 1.02%)d99.99( 51.61%)f99.99( 47.16%)g 0.29( 0.05%)
( 80.89%) 0.8994* N 4 s( 3.51%)p27.42( 96.31%)d 0.05( 0.17%)
(0.88731) BD ( 2) U 2- N 4
( 24.47%) 0.4946* U 2 s( 0.03%)p 8.49( 0.24%)d99.99( 43.89%)f99.99( 55.82%)g 0.92( 0.03%)
( 75.53%) 0.8691* N 4 s( 3.20%)p30.23( 96.64%)d 0.05( 0.16%)
(0.87891) BD ( 3) U 2- N 4
( 24.71%) 0.4971* U 2 s( 0.06%)p 2.76( 0.18%)d99.99( 36.02%)f99.99( 63.71%)g 0.41( 0.03%)
( 75.29%) 0.8677* N 4 s( 2.86%)p33.88( 96.98%)d 0.06( 0.16%)

**Table S30.** NBO Second order perturbation analysis (AMO) for $[Cp^{ttt}_2U(\mu\text{-}N)_2\{U(Cp^{ttt})(OEt_2)\}]$, **4**, s=3/2.

| Donor NBO | Acceptor NBO | E(2) kcal/mol |
|---|---|---|
| (0.85454) LP ( 1) N 3<br><br>s( 90.50%)p 0.10( 9.47%)d 0.00( 0.03%) | (0.21719) LV ( 1) U 1<br><br>s( 0.01%)p 1.00( 0.70%)d88.56( 61.94%)f53.37( 37.33%)g 0.02( 0.02%) | 40.86 |
| (0.85454) LP ( 1) N 3<br><br>s( 90.50%)p 0.10( 9.47%)d 0.00( 0.03%) | (0.18860) LV ( 2) U 1<br><br>s( 5.38%)p 0.04( 0.20%)d13.02( 70.09%)f 4.52( 24.32%)g 0.00( 0.01%) | 19.88 |
| (0.85454) LP ( 1) N 3<br><br>s( 90.50%)p 0.10( 9.47%)d 0.00( 0.03%) | (0.16650) LV ( 4) U 1<br><br>s( 0.31%)p 1.78( 0.56%)d99.99( 81.31%)f56.76( 17.81%)g 0.01( 0.00%) | 5.95 |
| (0.85454) LP ( 1) N 3<br><br>s( 90.50%)p 0.10( 9.47%)d 0.00( 0.03%) | (0.06898) LV ( 9) U 1<br><br>s( 65.02%)p 0.00( 0.26%)d 0.16( 10.39%)f 0.37( 24.32%)g 0.00( 0.01%) | 7.18 |
| (0.85454) LP ( 1) N 3<br><br>s( 90.50%)p 0.10( 9.47%)d 0.00( 0.03%) | (0.02355) LV (10) U 1<br><br>s( 0.05%)p70.98( 3.37%)d99.99( 31.95%)f99.99( 64.62%)g 0.30( 0.01%) | 11.91 |
| (0.85211) LP ( 1) N 4<br><br>s( 90.43%)p 0.11( 9.54%)d 0.00( 0.03%) | (0.21719) LV ( 1) U 1<br><br>s( 0.01%)p 1.00( 0.70%)d88.56( 61.94%)f53.37( 37.33%)g 0.02( 0.02%) | 47.78 |
| (0.85211) LP ( 1) N 4<br><br>s( 90.43%)p 0.11( 9.54%)d 0.00( 0.03%) | (0.18860) LV ( 2) U 1<br><br>s( 5.38%)p 0.04( 0.20%)d13.02( 70.09%)f 4.52( 24.32%)g 0.00( 0.01%) | 19.16 |
| (0.85211) LP ( 1) N 4 | (0.06898) LV ( 9) U 1 | 6.65 |

| | | |
|---|---|---|
| s( 90.43%)p 0.11( 9.54%)d 0.00( 0.03%) | s( 65.02%)p 0.00( 0.26%)d 0.16( 10.39%)f 0.37( 24.32%)g 0.00( 0.01%) | |
| (0.85211) LP ( 1) N 4<br><br>s( 90.43%)p 0.11( 9.54%)d 0.00( 0.03%) | (0.02355) LV (10) U 1<br><br>s( 0.05%)p70.98( 3.37%)d99.99( 31.95%)f99.99( 64.62%)g 0.30( 0.01%) | 11.35 |
| (0.92579) BD ( 1) U 2- N 3<br><br>( 20.49%) 0.4526* U 2 s( 0.14%)p 6.12( 0.88%)d99.99( 36.69%)f99.99( 62.24%)g 0.29( 0.04%)<br><br>( 79.51%) 0.8917* N 3 s( 2.89%)p33.51( 96.92%)d 0.06( 0.19%) | (0.16650) LV ( 4) U 1<br><br>s( 0.31%)p 1.78( 0.56%)d99.99( 81.31%)f56.76( 17.81%)g 0.01( 0.00%) | 6.24 |
| (0.89418) BD ( 2) U 2- N 3<br><br>( 25.76%) 0.5075* U 2 s( 0.04%)p 2.97( 0.12%)d99.99( 46.30%)f99.99( 53.52%)g 0.54( 0.02%)<br><br>( 74.24%) 0.8617* N 3 s( 1.61%)p60.88( 98.23%)d 0.10( 0.16%) | (0.21719) LV ( 1) U 1<br><br>s( 0.01%)p 1.00( 0.70%)d88.56( 61.94%)f53.37( 37.33%)g 0.02( 0.02%) | 5.21 |
| (0.89418) BD ( 2) U 2- N 3<br><br>( 25.76%) 0.5075* U 2 s( 0.04%)p 2.97( 0.12%)d99.99( 46.30%)f99.99( 53.52%)g 0.54( 0.02%)<br><br>( 74.24%) 0.8617* N 3 s( 1.61%)p60.88( 98.23%)d 0.10( 0.16%) | (0.16650) LV ( 4) U 1<br><br>s( 0.31%)p 1.78( 0.56%)d99.99( 81.31%)f56.76( 17.81%)g 0.01( 0.00%) | 6.17 |
| (0.89418) BD ( 2) U 2- N 3<br><br>( 25.76%) 0.5075* U 2 s( 0.04%)p 2.97( 0.12%)d99.99( 46.30%)f99.99( 53.52%)g 0.54( 0.02%) | (0.14513) LV ( 6) U 1<br><br>s( 0.06%)p 0.94( 0.06%)d99.99( 89.44%)f99.99( 10.44%)g 0.05( 0.00%) | 9.05 |

| | | |
|---|---|---|
| ( 74.24%) 0.8617* N 3 s( 1.61%)p60.88( 98.23%)d 0.10( 0.16%) | | |
| (0.88328) BD ( 3) U 2- N 3<br><br>( 23.80%) 0.4879* U 2 s( 0.03%)p 9.63( 0.27%)d99.99( 39.06%)f99.99( 60.61%)g 1.05( 0.03%)<br><br>( 76.20%) 0.8729* N 3 s( 5.00%)p18.98( 94.84%)d 0.03( 0.16%) | (0.21719) LV ( 1) U 1<br><br>s( 0.01%)p 1.00( 0.70%)d88.56( 61.94%)f53.37( 37.33%)g 0.02( 0.02%) | 13.40 |
| (0.88328) BD ( 3) U 2- N 3<br><br>( 23.80%) 0.4879* U 2 s( 0.03%)p 9.63( 0.27%)d99.99( 39.06%)f99.99( 60.61%)g 1.05( 0.03%)<br><br>( 76.20%) 0.8729* N 3 s( 5.00%)p18.98( 94.84%)d 0.03( 0.16%) | (0.16650) LV ( 4) U 1<br><br>s( 0.31%)p 1.78( 0.56%)d99.99( 81.31%)f56.76( 17.81%)g 0.01( 0.00%) | 19.85 |
| (0.88328) BD ( 3) U 2- N 3<br><br>( 23.80%) 0.4879* U 2 s( 0.03%)p 9.63( 0.27%)d99.99( 39.06%)f99.99( 60.61%)g 1.05( 0.03%)<br><br>( 76.20%) 0.8729* N 3 s( 5.00%)p18.98( 94.84%)d 0.03( 0.16%) | (0.08266) LV ( 8) U 1<br><br>s( 18.39%)p 0.01( 0.17%)d 1.09( 19.99%)f 3.34( 61.44%)g 0.00( 0.02%) | 6.04 |
| (0.92666) BD ( 1) U 2- N 4<br><br>( 19.11%) 0.4371* U 2 s( 0.16%)p 6.46( 1.02%)d99.99( 51.61%)f99.99( 47.16%)g 0.29( 0.05%)<br><br>( 80.89%) 0.8994* N 4 s( 3.51%)p27.42( 96.31%)d 0.05( 0.17%) | (0.16650) LV ( 4) U 1<br><br>s( 0.31%)p 1.78( 0.56%)d99.99( 81.31%)f56.76( 17.81%)g 0.01( 0.00%) | 5.74 |
| (0.88731) BD ( 2) U 2- N 4 | (0.16650) LV ( 4) U 1 | 17.28 |

| | | |
|---|---|---|
| ( 24.47%) 0.4946* U 2 s( 0.03%)p 8.49( 0.24%)d99.99( 43.89%)f99.99( 55.82%)g 0.92( 0.03%)<br><br>( 75.53%) 0.8691* N 4 s( 3.20%)p30.23( 96.64%)d 0.05( 0.16%) | s( 0.31%)p 1.78( 0.56%)d99.99( 81.31%)f56.76( 17.81%)g 0.01( 0.00%) | |
| (0.87891) BD ( 3) U 2- N 4<br><br>( 24.71%) 0.4971* U 2 s( 0.06%)p 2.76( 0.18%)d99.99( 36.02%)f99.99( 63.71%)g 0.41( 0.03%)<br><br>( 75.29%) 0.8677* N 4 s( 2.86%)p33.88( 96.98%)d 0.06( 0.16%) | (0.21719) LV ( 1) U 1<br><br>s( 0.01%)p 1.00( 0.70%)d88.56( 61.94%)f53.37( 37.33%)g 0.02( 0.02%) | 10.09 |
| (0.87891) BD ( 3) U 2- N 4<br><br>( 24.71%) 0.4971* U 2 s( 0.06%)p 2.76( 0.18%)d99.99( 36.02%)f99.99( 63.71%)g 0.41( 0.03%)<br><br>( 75.29%) 0.8677* N 4 s( 2.86%)p33.88( 96.98%)d 0.06( 0.16%) | (0.16650) LV ( 4) U 1<br><br>s( 0.31%)p 1.78( 0.56%)d99.99( 81.31%)f56.76( 17.81%)g 0.01( 0.00%) | 10.72 |
| (0.87891) BD ( 3) U 2- N 4<br><br>( 24.71%) 0.4971* U 2 s( 0.06%)p 2.76( 0.18%)d99.99( 36.02%)f99.99( 63.71%)g 0.41( 0.03%)<br><br>( 75.29%) 0.8677* N 4 s( 2.86%)p33.88( 96.98%)d 0.06( 0.16%) | (0.14513) LV ( 6) U 1<br><br>s( 0.06%)p 0.94( 0.06%)d99.99( 89.44%)f99.99( 10.44%)g 0.05( 0.00%) | 9.18 |
| (0.85454) LP ( 1) N 3<br><br>s( 90.50%)p 0.10( 9.47%)d 0.00( 0.03%) | (0.06901) BD*( 1) U 2- N 3<br><br>( 79.51%) 0.8917* U 2 s( 0.14%)p 6.12( 0.88%)d99.99( 36.69%)f99.99( 62.24%)g 0.29( 0.04%)<br><br>( 20.49%) -0.4526* N 3 s( 2.89%)p33.51( 96.92%)d 0.06( 0.19%) | 10.62 |

| | | |
|---|---|---|
| (0.85211) LP ( 1) N 4<br><br>s( 90.43%)p 0.11( 9.54%)d 0.00( 0.03%) | (0.02323) LV ( 4) U 2<br><br>s( 2.40%)p 0.91( 2.19%)d20.19( 48.52%)f19.52( 46.89%)g 0.00( 0.00%) | 11.93 |
| (0.85211) LP ( 1) N 4<br><br>s( 90.43%)p 0.11( 9.54%)d 0.00( 0.03%) | (0.07391) BD*( 1) U 2- N 4<br><br>( 80.89%) 0.8994* U 2 s( 0.16%)p 6.46( 1.02%)d99.99( 51.61%)f99.99( 47.16%)g 0.29( 0.05%)<br><br>( 19.11%) -0.4371* N 4 s( 3.51%)p27.42( 96.31%)d 0.05( 0.17%) | 12.30 |

**Table S31.** DFT computed MOs for [$Cp^{ttt}{}_2U(\mu\text{-}N)_2\{U(Cp^{ttt})(OEt_2)\}$], **4**, s=3/2. (a) αMO-HOMO-3 (b) αMO-HOMO-2 (c) αMO-HOMO-1 (d) αMO-HOMO (e) αMO-LUMO (f) βMO-LUMO (g) spin density plot

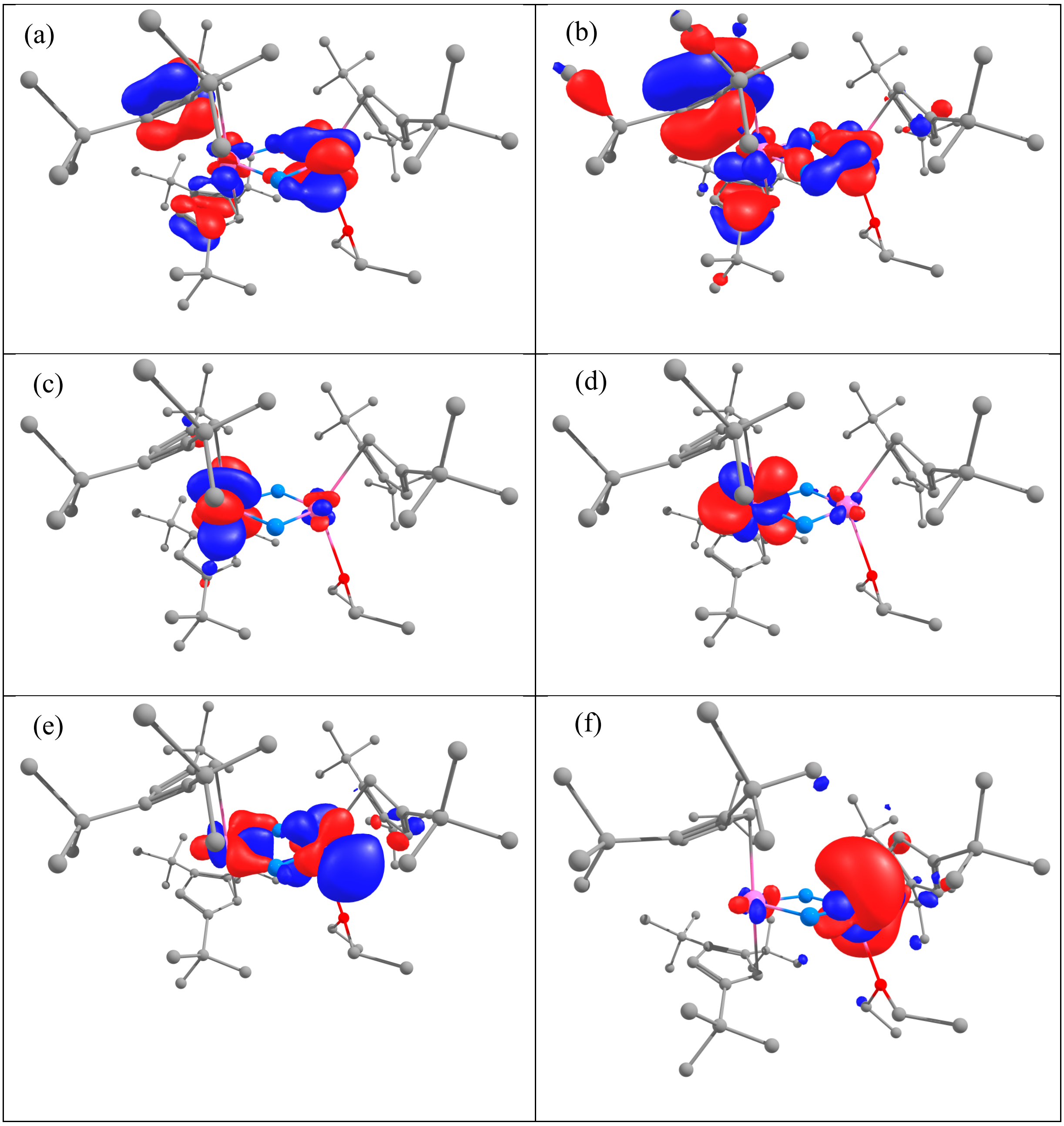

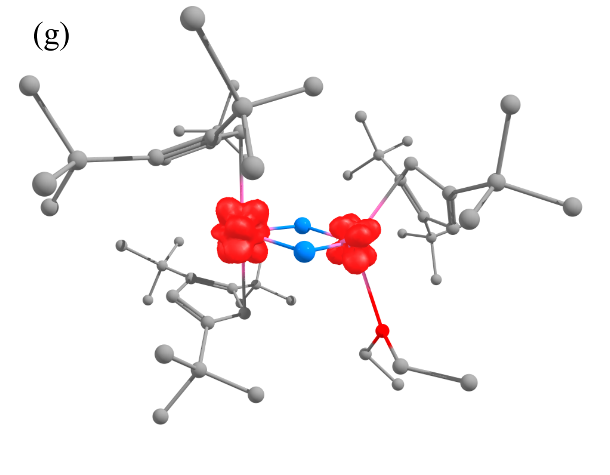


**Table S32.** Computed BCP descriptors for [Cp$^{ttt}{}_2$U(μ-N)$_2${U(Cp$^{ttt}$)(OEt$_2$)}], **4**, s=3/2.

| | $\rho$(r) | $\nabla^2\rho$(r) | *G*(r) | *V*(r) | *H*(r) | $\varepsilon$ |
|---|---|---|---|---|---|---|
| U1-N3 | 0.12 | 0.25 | 0.11 | -0.16 | -0.05 | 0.06 |
| U1-N4 | 0.13 | 0.25 | 0.12 | -0.17 | -0.05 | 0.06 |
| U2-N3 | 0.22 | 0.17 | 0.21 | -0.37 | -0.17 | 0.06 |
| U2-N4 | 0.22 | 0.19 | 0.21 | -0.37 | -0.16 | 0.05 |

**Figure S68.** Laplacian distribution in $U_2N_2$ core of [Cp$^{ttt}{}_2$U(μ-N)$_2${U(Cp$^{ttt}$)(OEt$_2$)}], **4**, s=3/2.

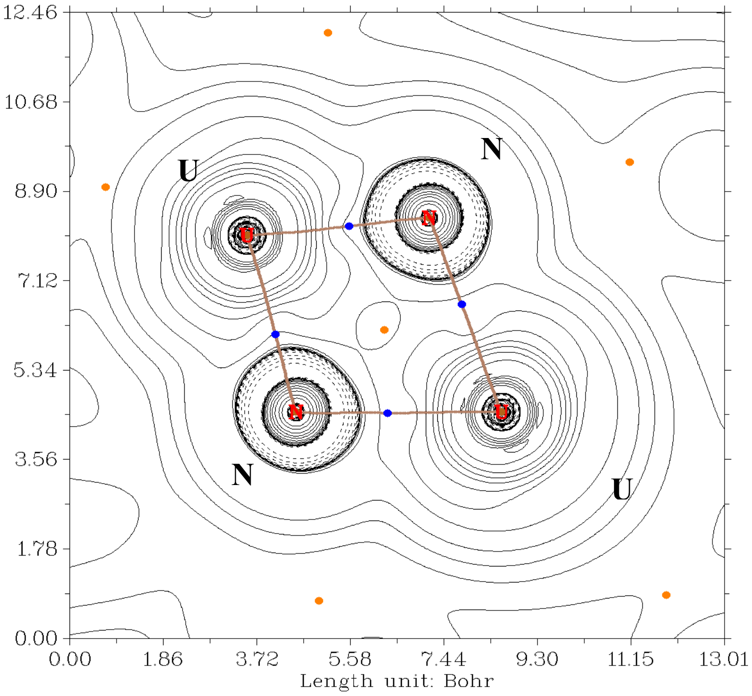

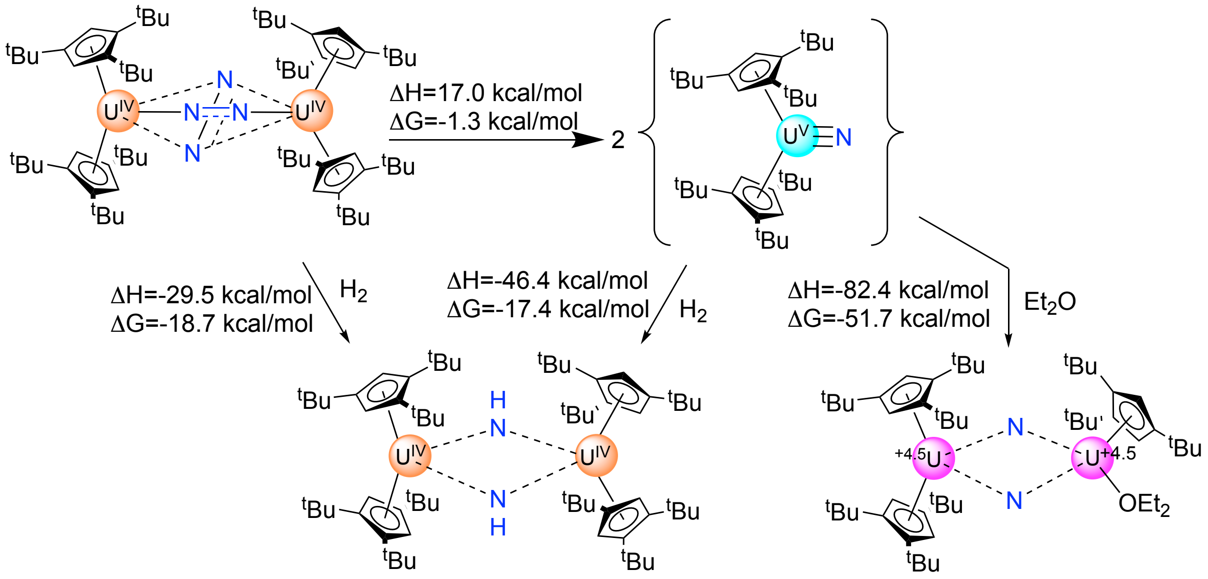


**Scheme S1.** Thermochemical data highlighting the viability of the transformation of **2** into **A** and **4**.

## 9. References


1. M. Reiners, N. Ehrlich, M. D. Walter, *Inorganic Syntheses*, 2018, **37**, 199-204.

2. D. E. Bergbreiterand, J. M. Killough, *J. Am. Chem. Soc.* 1978, **100**, 2126-2134.

3. M. J. Monreal, R. K. Thomson, T. Cantat. N. E. Travia, B. L. Scott, J. K. Kiplinger, *Organometallics*, 2011, **30**, 2031-2038.

4. T. Li, D. Wang, Y. Heng, G. Hou, G. Zi, M. D. Walter, *Organometallics* 2023, **42**, 392-406.

5. E. V. Dehmlow, C. Bollmann, *Liebigs Ann*. 1995, 837-839.

6. G. Sheldrick, A short history of SHELX. *Acta Crystallogr*. 2008, **64**, 112-122.

7. G. Sheldrick, SHELXT - Integrated space-group and crystal-structure determination. *Acta Crystallogr*. 2015, Sect. A: Found. Adv. **71**, 3-8.

8. G. Sheldrick, Crystal structure refinement with SHELXL. *Acta Crystallogr.* 2015, Sect. C: Struct. Chem. **71**, 3-8.

9. O. V. Dolomanov, L. J. Bourhis, R. J. Gildea, J. A. K. Howard, H. Puschmann, OLEX2: a complete structure solution, refinement and analysis program. *J. Appl. Crystallogr*. 2009, **42**, 339-341.

10. I. J. Bruno, J. C. Cole, P. R. Edgington, M. Kessler, C. F. Macrae, P. McCabe, J. Pearson, R. Taylor, *Acta Cryst*. 2002, **B58**, 389-397.

11. a) A. D. Becke, *J. Chem. Phys*. 1993, **98**, 5648; b) K. Burke, J. P. Perdew, W. Yang, in Electronic Density Functional Theory: Recent Progress and New Directions, Eds: J. F. Dobson, G. Vignale, M. P. Das, Plenum, New York, 1998.

12. a) M. Dolg, U. Wedig, H. Stoll, H. Preuss, *J. Chem. Phys*. 1987, **86**, 866; b) A. Hollwarth, M. Bohme, S. Dapprich, A.W. Ehlers, A. Gobbi, V. Jonas, K.F. Kohler, R. Stegmann, A. Veldkamp, G. Frenking, *J. Chem. Phys*. 1993, **208**, 237; c) W. Kuechle, M. Dolg, H. Stoll, H. Preuss, *J. Chem. Phys*. **1994**, *100*, 7535; d) X. Cao, M. Dolg, H. Stoll, *J. Chem. Phys*. 2023, **118**, 487 cf. also X. Cao, M. Dolg, *J. Molec. Struct*. (Theochem) 2004, **673**, 203.

13. a) P. C. Hariharan, J. A. Pople, *Theor. Chim. Acta* 1973, **28**, 213; b) W. J. Hehre, R. Ditchfield, J. A. Pople, *J. Chem. Phys*. 1972, **56**, 2257.

14. Gaussian 09, Revision D.01: M. J. Frisch, G. W. Trucks, H. B. Schlegel, G. E. Scuseria, M. A. Robb, J. R. Cheesman, G. Scalmani, V. Barone, B. Mennucci, G. A. Petersson, H. Nakatsuji, M. Caricato, X. Li, H. P. Hratchian, A. F. Izmaylov, J. Bloino, G. Zheng, J. L. Sonnenberg,

M. Hada, M. Ehara, K. Toyota, R. Fukuda, J. Hasegawa, M. Ishida, T. Nakajima, Y. Honda, O. Kitao, H. Nakai, T. Vreven, J. A., Jr. Montgomery, J. E. Peralta, F. Ogliaro, M. Bearpark, J. J. Heyd, E. Brothers, K. N. Kudin, V. N. Staroverov, R. Kobayashi, J. Normand, K. Raghavachari, J. C. Burant, S. S. Iyengar, J. Tomasi, M. Cossi, N. Rega, M. J. Millam, M. Klene, J. E. Knox, J. B. Cross, V. Bakken, C. Adamo, J. Jaramillo, R. Gomperts, R. E. Stratmann, O. Yazyev, A. J. Austin, R. Cammi, C. Pomelli, J. W. Ochterski, R. L. Martin, K. Morokuma, V. G. Zakrzewski, G. A. Voth, P. Salvador, J. J. Dannenberg, S. Dapprich, A. D. Daniels, O. Farkas, J. B. Foresman, J. V. Ortiz, J. Cioslowski and D. J. Fox, Gaussian Inc., 2009, Wallingford CT.
